# Nuclear lipid droplets and nuclear damage in *Caenorhabditis elegans*

**DOI:** 10.1371/journal.pgen.1009602

**Published:** 2021-06-16

**Authors:** Jose Verdezoto Mosquera, Meghan C. Bacher, James R. Priess

**Affiliations:** 1 Fred Hutchinson Cancer Research Center, Seattle, Washington, United States of America; 2 Molecular and Cellular Biology Program, University of Washington, Seattle, Washington, United States of America; 3 Department of Biology, University of Washington, Seattle, Washington, United States of America; Washington State University, UNITED STATES

## Abstract

Fat stored in the form of lipid droplets has long been considered a defining characteristic of cytoplasm. However, recent studies have shown that nuclear lipid droplets occur in multiple cells and tissues, including in human patients with fatty liver disease. The function(s) of stored fat in the nucleus has not been determined, and it is possible that nuclear fat is beneficial in some situations. Conversely, nuclear lipid droplets might instead be deleterious by disrupting nuclear organization or triggering aggregation of hydrophobic proteins. We show here that nuclear lipid droplets occur normally in *C*. *elegans* intestinal cells and germ cells, but appear to be associated with damage only in the intestine. Lipid droplets in intestinal nuclei can be associated with novel bundles of microfilaments (nuclear actin) and membrane tubules that might have roles in damage repair. To increase the normal, low frequency of nuclear lipid droplets in wild-type animals, we used a forward genetic screen to isolate mutants with abnormally large or abundant nuclear lipid droplets. Genetic analysis and cloning of three such mutants showed that the genes encode the lipid regulator SEIP-1/seipin, the inner nuclear membrane protein NEMP-1/Nemp1/TMEM194A, and a component of COPI vesicles called COPA-1/α-COP. We present several lines of evidence that the nuclear lipid droplet phenotype of *copa-1* mutants results from a defect in retrieving mislocalized membrane proteins that normally reside in the endoplasmic reticulum. The *seip-1* mutant causes most germ cells to have nuclear lipid droplets, the largest of which occupy more than a third of the nuclear volume. Nevertheless, the nuclear lipid droplets do not trigger apoptosis, and the germ cells differentiate into gametes that produce viable, healthy progeny. Thus, our results suggest that nuclear lipid droplets are detrimental to intestinal nuclei, but have no obvious deleterious effect on germ nuclei.

## Author summary

Several human disorders such as obesity are associated with abnormal fat storage. Cells normally store fat in cytoplasmic organelles called lipid droplets. However, recent studies have shown that fat can also form inside of the cell nucleus, and the effects of nuclear fat are not known. Here we use the cell biology and genetics of the model organism *C*. *elegans* to study the causes and consequences of nuclear fat. We show that intestinal cells can contain nuclear fat, particularly during high-low-high changes in cytoplasmic fat that involve *de novo* fat synthesis. Nuclear fat is associated with multiple changes in intestinal nuclei that appear to represent damage and repair. Germ nuclei that normally differentiate into oocytes can also contain nuclear fat. In germ cells, however, even high levels of nuclear fat appear to cause little or no damage. Our results suggest that intestinal nuclei and germ cell nuclei might have different responses to nuclear fat in part because they differ in chromosomal organization at the nuclear envelope.

## Introduction

Lipid droplets (LDs) are the major form of fat storage, and provide a reservoir of energy and building blocks for membrane growth and repair. LDs can have additional, important functions in cell signaling, development, and in stress responses [1,2]. For example, lipid droplets can serve as storage sites for histones in *Drosophila* embryos, and can contain polyubiquitinated proteins in yeast cells that experience acute lipid stress [3,4]. Excess lipid accumulation is associated with a wide range of human pathologies from atherosclerosis to obesity [5]. At the cellular level, dysfunction in lipid storage or metabolism can lead to free fatty acid-induced lipotoxicity, with cascading trauma to cell membranes and other components [6]. Many types of cells contain LDs, although the numbers and sizes of LDs can vary enormously depending on physiological state or culture conditions. For example, the volume of stored lipid in cultured cells can rapidly change severalfold upon fatty acid supplementation or withdrawal [7].

LDs consist of neutral lipids, largely triacylglycerides and sterol esters, that are surrounded by a surface monolayer of polar phospholipids. LDs can grow by fusing with other LDs, but *de novo* formation occurs in the endoplasmic reticulum (ER) [8,9]. Lipid appears to accumulate among the acyl chains on the lipid bilayer of the ER membrane. The lipid coalesces and expands into a stable "lens" that separates the two leaflets of the bilayer, eventually budding into the cytoplasm as a small LD. Homooligomers of the protein seipin appear to form toroids around ER-LD contact sites [10–12], and function in stabilizing the lipid lens and in trafficking protein/lipid into the developing LD [9,13]. Loss-of-function mutations in human seipin cause BSCL type 2, one of the most severe lipodystrophies, while gain-of-function mutations in seipin cause pathologies of the nervous system [14,15]. Among the many known regulators of lipid droplets, COPI (coat protein complex I) components appear to have a role in LD morphology, protein composition, and lipolysis [16–18]. For example, COPI-dependent membrane bridges appear to link LDs with the ER, allowing ER-localized regulatory proteins to load onto the LD surface [19]. Some ER-localized enzymes involved in triglyceride synthesis appear to remain anchored in the phospholipid monolayer of the LD surface after budding, and drive the continued growth of the LD [20].

LDs are often found in the cytoplasm adjacent to the outer nuclear membrane, which is continuous with ER membranes [21]. However, several recent studies have shown that LDs also occur inside nuclei [22]. The function(s) of nuclear lipid droplets (nLDs) are not known, but possibilities include providing lipid for the growth of nuclear membranes, storage sites for normal proteins or for unfolded, hydrophobic proteins, and as sites for the detoxification of hydrophobic substances [23]. Few or no nLDs have been observed in some types of cells that contain abundant cLDs, such as adipocytes, or stimulated fibroblasts, but nLDs have been found in multiple types of liver-derived cell lines [24] and in human patients with fatty-liver disease, or hepatic steatosis [22]. Obesity-related hepatic steatosis is estimated to affect 20–30% of the population of North America, including very high percentages of patients with morbid obesity or type 2 diabetes (reviewed in [20]). Although the normal mammalian liver has few cytoplasmic LDs (hereafter cLDs) in the fed state, even short periods of fasting cause an enormous accumulation of cLDs, as lipids are mobilized from adipose tissue and stored in liver cells to reserve energy for vital functions.

nLDs that form at the envelope presumably enter the nucleoplasm by penetrating the nuclear lamina and associated peripheral heterochromatin [24,25]. Thus, nLDs have the potential to impact chromatin organization or the integrity of the lamina. Lamins are major components of the lamina, and interact directly or indirectly with proteins that have diverse roles in nuclear biology, including nuclear architecture and chromatin organization. *C*. *elegans* has a single lamin, LMN-1, which is most similar to B-type lamins in other systems [26,27]. Mutations in lamin and other lamina components cause severe developmental defects and shortened lifespan in *C*. *elegans*, and are responsible for numerous human diseases including muscle dystrophies, lipodystrophies and premature aging (reviewed in [28]).

The goals of the present study were to address whether nLDs are (1) present in selected *C*. *elegans* tissues, and (2) drive nuclear damage. In particular, we hoped to determine whether nLDs might be a factor in the extraordinary number of apoptotic deaths that occur in normal germ cell development [29]. Most studies on nLDs in cultured cells induce nLD formation with oleic acid-supplemented media. Here, we analyzed two fatty tissues, the intestine and the gonad, in animals fed the standard laboratory diet of *E*. *coli* strain OP50. The intestine is the major fat-storage tissue in *C*. *elegans*; it has been studied extensively as a genetic model of fat storage and obesity, and compared with both liver and adipose tissue in higher animals [30–32]. We focused on the self-fertile reproductive period when adult hermaphrodites naturally undergo large changes in fat; these changes involve yolk production by the intestine, and egg production by the gonad. Larval hermaphrodites produce only low-fat sperm, but adult hermaphrodites switch to producing high-fat oocytes. The spermatogenesis/oogenesis switch is associated with a massive reallocation or transfer of metabolic resources, such as fat precursors and yolk lipoproteins, from the intestine to the gonad. Those resources are in turn lost from the gonad as eggs are laid. Remarkably, the peak amount of eggs laid in one day is equivalent to the entire body weight of the adult, and just one yolk protein, YP170, accounts for about 25% of protein synthesis in the intestine [33,34].

During the self-fertile period, unmated hermaphrodites use their stored sperm to produce limited numbers of self-progeny, and egg production ceases once those sperm are depleted. However, hermaphrodites maintain the potential for producing far larger numbers of cross-progeny if they encounter males and mate after the self-fertile period. Thus, any nuclear defects in the self-fertile period would likely result from specific trauma, and be repaired, rather than from a generalized deterioration associated with post-reproductive senescence. The hermaphrodite gonad provides a sensitive system for addressing whether physiological or cellular changes are deleterious, because germ cells can be triggered to undergo apoptosis by a large variety of stresses that include DNA damage, infection, heat, and starvation [35]. Indeed, more than 50% of germ cells undergo apoptosis in wild-type animals grown under standard culture conditions; the mechanisms that trigger this natural or "physiological" apoptosis are largely unknown [29,36–38]. Importantly, mutations that block germ cell apoptosis are homozygous viable, allowing possible defects to be analyzed in the surviving progeny. Although intestinal cells lack an apoptosis pathway, they undergo necrosis in response to various traumas such as bacterial infection or hypo-osmotic shock [39,40].

We show that nLDs occur in normal intestinal nuclei, and are associated with complex changes in nuclei during the self-fertile period. These nuclear changes include the development of bundled microfilaments, the formation of a type I nucleoplasmic reticulum, and the generation of lamin-coated vesicles. We show that a few intestinal nuclei appear to rupture at sites adjacent to large nLDs, and that some nLDs appear to initiate a series of events that culminate in the formation of large nuclear cysts and the removal of aberrant nucleoplasm. These nuclear changes occur predominantly in a subset of adult intestinal cells that have the greatest fluctuation in stored fat during the self-fertile reproductive period. We show that germ cell nuclei also form nLDs, but the nuclei appear relatively unchanged. Because of the simplicity of nLD formation in germ cells, we used the gonad in a forward genetic screen for mutants with abnormal numbers or sizes of nLDs. Molecular analysis of four mutations showed that they affect the inner nuclear membrane protein NEMP-1/NEMP1, the lipid droplet regulator SEIP-1/seipin, and the COPI proteins COPA-1/α-COP and COPB-2/β’-COP. Surprisingly, these mutants suggest that a large volume of a meiotic germ nucleus can be filled with fat without triggering apoptosis, and that embryos derived from such germ cells are viable and healthy. Our results suggest that the nLD-associated defects in intestinal nuclei, and the lack of similar defects in germ cells, result from structural differences in the two types of nuclei.

## Results

### Background

**Fig 1:**
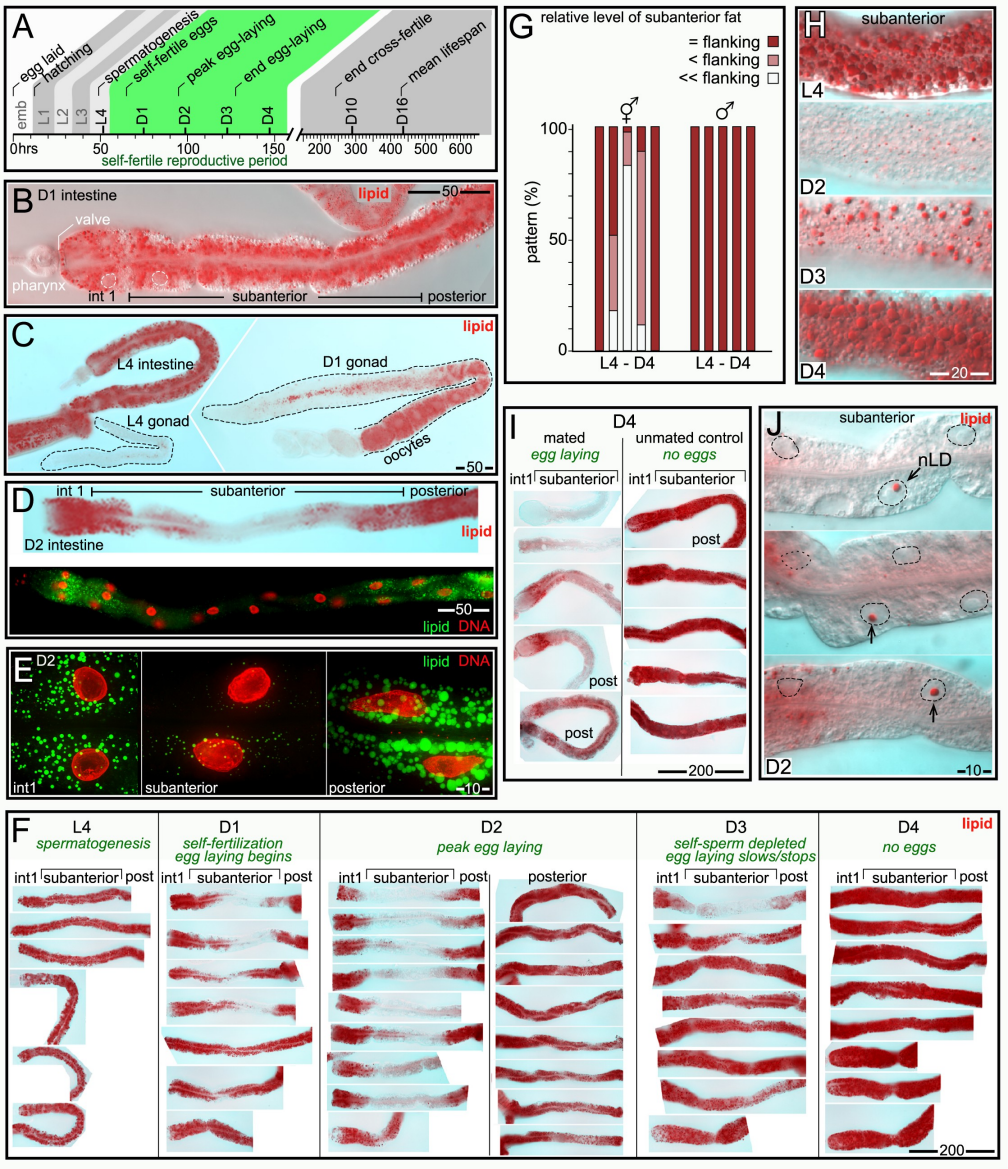
Changes in intestinal fat during the self-fertile reproductive period. (A) Timeline of *C*. *elegans* development at 20°C, showing key events (adapted from [111]). (B) Anterior half of a D1 intestine imaged by Differential Interference Contrast (DIC) microscopy after staining for fat (red, oil red O); two intestinal nuclei are outlined in white. Note that the intestine has a much higher level of fat than the pharynx. The intestine in a newly hatched larva consists of groups of cells called int1-int9, and additional cells are added during post-embryonic development. This report defines the term subanterior to refer collectively to cells in the anterior half of the intestine, but excluding the anteriormost, int1 group. (C) Comparison of fat levels in hermaphrodite tissues. Note the large difference in fat between the L4 and adult gonads, which produce sperm and oocytes, respectively. (D) Images of D2 intestines stained for fat with oil red O (red, top panel) or with BODIPY (green, bottom panel). Both stains show a relative reduction of fat in the subanterior region, with the greatest variation in the int2 cell group. (E) High magnification of cells in a D2 intestine, comparing fat (green, BODIPY) in cells in the int1 group, cells in the subanterior region, and cells in the posterior region. For contrast, the DAPI-stained nuclei are shown in red. Each image is a projection of a 5 μm optical z-stack. (F) Comparison of intestinal fat in animals at the indicated stages. Approximately equal numbers of anterior and posterior dissections were performed as quantified in **Fig 1G**, but posterior dissections are only shown for the D2 timepoint. Note that fat is depleted, then replenished, in the subanterior region. (G) The chart shows the level of fat in the subanterior region compared with the flanking regions (the int1 group and the posterior cells); data is shown for both hermaphrodites and males. The total numbers of hermaphrodite/male intestines examined were L4 (48/17), D1 (37/21), D2 (52/14), D3 (41/22) and D4 (47/16). (H) Comparison of fat in the subanterior region of staged intestines as indicated. (I) Comparison of fat in D4 mated animals (left) with synchronous, unmated D4 controls (right). For this experiment, males were mated with D2 hermaphrodites for 24 hours and then removed. Mated D4 hermaphrodites were identified as containing fertilized eggs. (J) Candidate nLDs (arrows) in the subanterior region of D2 intestines. Note that the candidate nLDs are far larger than cLDs in the surrounding cytoplasm. Nuclei are indicated by dashed lines. Scale bar sizes are shown in microns for all panels.

*C*. *elegans* undergoes four larval stages, called L1-L4, before becoming an adult. The nomenclature used to indicate adult ages varies in the literature; here, a Day 1 (D1) adult was picked as a mid-L4 larva and allowed to develop for 24 hours (Fig 1A). This study focuses on unmated hermaphrodites during the self-fertile reproductive period, between L4 and D4 (Fig 1A). The mean lifespan is D16 with the standard diet and culture conditions used here, so D1-D4 adults represent the first quarter of adult life [41].

During feeding, ingested bacteria are crushed by a muscular pharynx, then passed through a small epithelial valve and into the intestine for digestion (Fig 1B). The 30–34 nuclei in the adult intestine undergo endoreduplication to reach a ploidy of 32C, and are much larger than most other *C*. *elegans* nuclei [42]. Cells in the intestine form natural groupings; the anteriormost four cells are called the int1 group, and more posterior groups consist of two cells each.

### The intestine undergoes region-specific changes in fat during the self-fertile period

We examined fat in aldehyde-fixed intestines and gonads stained with the lipid dyes oil red O or BODIPY. L4 gonads, which produce low-fat sperm, had very low levels of fat, as expected (Fig 1C). D1 adult hermaphrodite gonads, which produce high-fat oocytes, were larger and contained a much higher level of fat (Fig 1C). All L4 intestines had a high, uniform level of fat (Fig 1C), as did about half of D1 intestines (Fig 1B). Unexpectedly, several D1 intestines and most D2 intestines had a non-uniform distribution of fat: Fat levels were high in the anteriormost cells (int1 group), and in all cells in the posterior half of the intestine (Fig 1D). However, fat levels were much lower in the region between the anterior and posterior cell groups, here termed the subanterior region (Fig 1D). Fat depletion in the subanterior region was associated with a large, relative decrease in the sizes and numbers of cLDs (Fig 1E). We next examined fat in synchronous populations of L4-D4 animals, covering the self-fertile period. Animals were dissected near the head to release the anterior half of the intestine (int1 cells plus the subanterior region), or near the tail to release the posterior half of the intestine (Fig 1F, quantified in 1G). We found that fat depleted in the subanterior region of D2 intestines appeared to be restored to high levels between D3 and D4 (Fig 1F–1H), as the self-sperm of unmated hermaphrodites are depleted and egg production ends. We next examined mated hermaphrodites that continue egg production beyond D4. The mated D4 hermaphrodites did not restore fat in the subanterior region, and showed additional depletion of fat in the flanking regions (Fig 1I).

We next used transmission electron microscopy (TEM) to examine intestinal cell cytoplasm in L4, D2, and D3 hermaphrodites (S1 Fig). As expected, only the subanterior cells showed a large loss of cLDs between L4 and D2, followed by an increase at D3. However, all intestinal cells showed major changes in cytoplasmic components after the L4 stage, including a loss of glycogen and a decrease in the sizes and numbers of yolk granules. Thus, all intestinal cells appear to mobilize resources to support egg production, but only the subanterior cells undergo large, high-low-high changes in fat.

### nLDs occur in L4 and adult intestinal nuclei, and can be associated with nuclear ruptures

**Fig 2:**
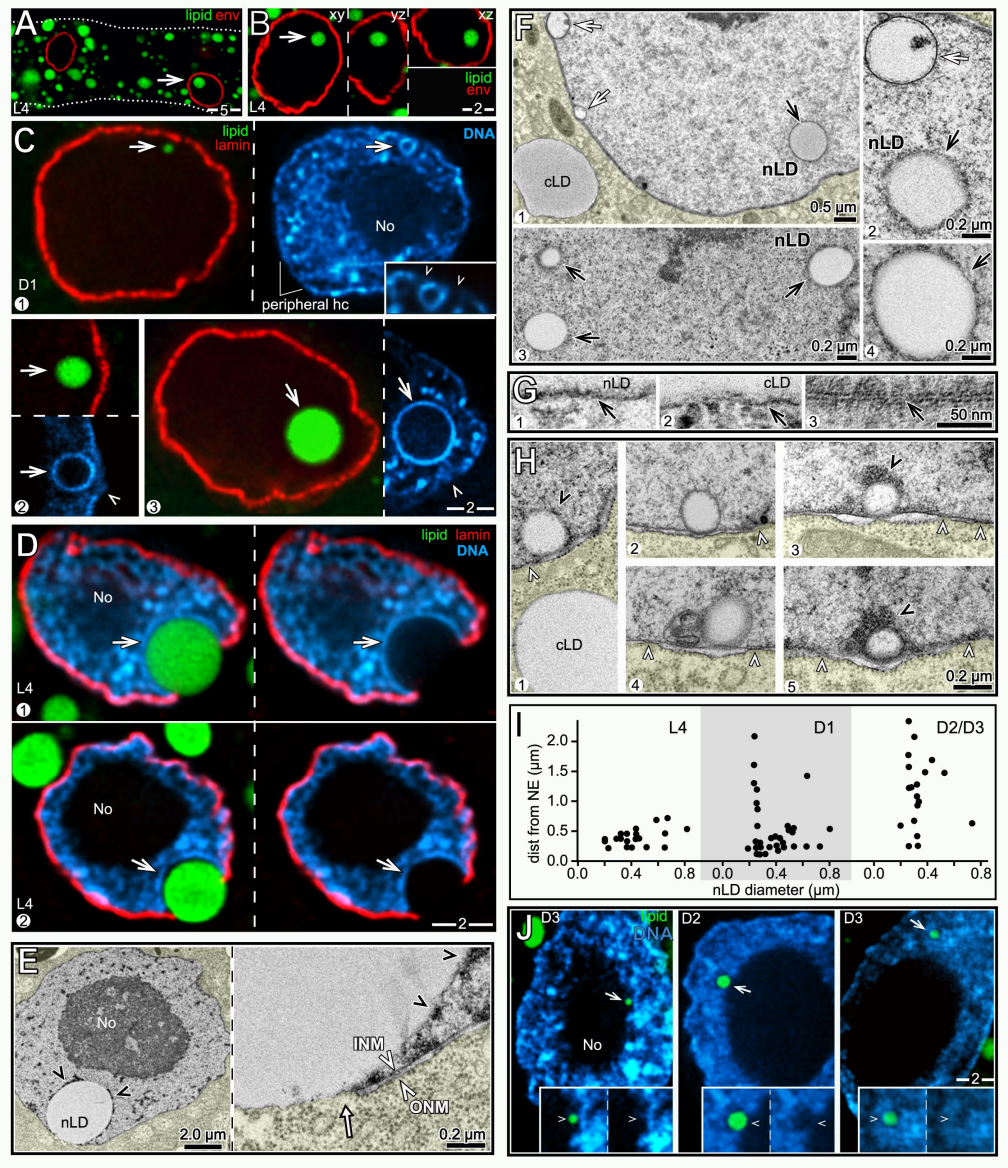
nLDs in hermaphrodite intestinal cells. (A) Image of two nuclei in an L4 intestine stained for lipid (green, BODIPY) and for the nuclear envelope (red, NPP-9/RanBP2). The nucleus at right has a candidate nLD (arrow). (B) Single nLD (green, BODIPY) viewed in three, orthogonal optical planes from a z stack through the entire nucleus (red, NPP-9/RanBP2). (C) Three examples of D1 nuclei with nLDs; nuclei are stained for lipid (green, BODIPY) and lamin (red, LMN-1). Each nLD is surrounded by a ring of condensed chromatin, or heterochromatin (blue, DNA), and is adjacent to a region of the envelope that appears relatively deficient in peripheral heterochromatin (arrowheads). No = nucleolus in this and all Figures. (D) L4 intestinal nuclei showing examples of giant nLDs (arrows) associated with apparent ruptures of the nuclear envelope (red, LMN-1/lamin). Other giant nLDs were adjacent to lamin-deficient regions of the envelope (see S2 Fig). Several observations suggest the ruptures are not artifacts of sample preparation. First, the positions of ruptured nuclei were not, in general, near dissection sites. Second, sample compression was minimized by supporting coverslips with precision-sized glass beads only slightly smaller than the diameter of the intestine; ruptures also were present in samples prepared for TEM (see **Fig 2E**) that were never compressed. Finally, intentional compression of fixed intestines caused widespread fusion, and highly abnormal shapes, of cLDs that did not resemble the images presented here. (E) TEM of a D1 intestinal nucleus showing a giant nLD at a nuclear rupture; the inset show both the INM and ONM are broken (arrow). The nLD is partially surrounded by electron-dense material (black arrowheads) resembling heterochromatin at the nuclear periphery and elsewhere. For clarity, the cytoplasm is tinted yellow here and in other micrographs below. (F) TEM showing nLDs (black arrows) in D1 intestinal nuclei. The black arrow in panel 4 indicates the exposed surface of an nLD at a gap in the electron-dense coat. The white arrows in panels 1 and 2 indicate distinct types of nuclear bodies called kernel vesicles (see below). The kernel vesicles are surrounded by conventional lipid bilayer membranes, which stain more intensely than the lipid monolayer surfaces of lipid droplets. (G) High magnification TEM images comparing the exposed surface of an nLD (panel 1) with the lipid monolayer surface of a cLD (panel 2) and with a typical lipid bilayer membrane (panel 3; taken from the plasma membrane around an intestinal microvillus). (H) TEM images of small nLDs by the nuclear envelope, showing variable electron-dense coatings (black arrowheads); white arrowheads indicate nuclear pores. (I) Plot showing the distances of submicron nLDs from the nuclear envelope; data are from optical z-stacks of stained nuclei as in Fig 2C. The smallest nLDs detectable by this technique were about 0.2 μm from the lamina, and appeared to be separated from the lamina by the thickness of the heterochromatin coating (see panel 1 in Fig 2C). Note that by the D2 and D3 stages most of the smallest nLDs are not adjacent to the envelope. (J) Examples of submicron nLDs (arrows) in D2 and D3 nuclei that are not adjacent to the envelope. The insets show that these nLDs do not appear to have distinct heterochromatin coats. Scale bars in microns as indicated.

In the above experiments we observed candidate nLDs in the subanterior region of some D1 and D2 intestines, where the nLDs were prominent due to the low level of fat in the surrounding cytoplasm (arrows in Fig 1J, n = 7/104 intestines with nLDs). To confirm the candidate nLDs were not instead cLDs embedded in invaginations or inpocketings of the nuclear envelope, we stained intestines with antibodies that recognize either lamin or nuclear pores (Fig 2A). Orthogonal planes though optical z-stacks confirmed that the vast majority of candidate nLDs were entirely within nuclei (Fig 2B). The frequency of nLDs varied considerably between intestines of different ages, and between different preparations of intestines at the same age. For example, the frequency of D2 intestinal nuclei with nLDs ranged from 5.6 to 22.4% in separate experiments (Table 1). Single intestines typically contained no more than one or two nuclei with nLDs, but a few exceptional L4 intestines contained 5–8 nuclei with large nLDs (> 2 μm).

We considered whether the variation in nLDs might be due to lipid extraction during sample preparation. Our immunostaining protocol uses formaldehyde fixation followed by brief detergent permeabilization; the detergent is essential for antibody penetration of the nuclear membrane, but is not essential to stain either lipid or DNA. Thus, we split a set of fixed intestines into two groups, and treated only one group with detergent before staining for lipid and DNA. D1 and D2 intestines that were not treated with detergent had about 2–3 fold more nuclei with nLDs than detergent-treated intestines, with the major difference being small nLDs below 0.5 μm (Table 1 and see S2 Fig for scoring protocol). The untreated intestines also appeared to have more small nLDs per nucleus. For example, detergent-treated nuclei typically had no more than one or two nLDs, but untreated nuclei often had three or four nLDs, with a maximum of eight (S2 Fig). These results and our TEM analysis of glutaraldehyde-fixed intestines (see below) suggest that our immunostaining protocol preserves most large nLDs, but underestimates the number of small nLDs.

**Table 1:** nLDs in intestinal nuclei.

genotype	Age	sex	group*	detergent	nLD (% nuclei)	rupture (%)	total nuclei
WT	L4	herm	single	Triton X100	9.3	NA	525
WT	L4	herm	single	Triton X100	8.2	0.3	476
WT	L4	herm	single	Triton X100	1.5	0.0	129
WT	L4	herm	single	Triton X100	1.6	0.9	115
WT	L4	herm	single	Triton X100	9.5	0.0	95
WT	L4	herm	single	Triton X100	4.2	0.0	94
WT	L4	herm	single	Triton X100	4.4	1.1	90
WT	D1	herm	single	Triton X100	5.3	0.0	169
WT	D1	herm	single	Triton X100	5.6	NA	162
WT	D2	herm	single	Triton X100	5.6	NA	902
WT	D2	herm	single	Triton X100	5.9	NA	387
WT	D2	herm	single	Triton X100	22.4	NA	161
WT	D3	herm	single	Triton X100	5.4	NA	353
WT	D3	herm	single	Triton X100	2.1	0.0	145
WT	D3	herm	paired	Triton X100	11.0	NA	101
WT	D3	herm		Triton X100	14.7	NA	61
WT	D4	herm	single	Triton X100	6.8	0.0	88
WT	L4	male	single	Triton X100	0.5	NA	388
WT	L4	male	single	Triton X100	0.0	0.0	415
WT	D1	male	single	Triton X100	0.8	0.0	118
WT	D1	male	single	Triton X100	0.8	0.0	370
WT	D2	male	single	Triton X100	0.0	0.0	90
WT	D2	male	single	Triton X100	0.7	0.0	134
WT	D3	male	single	Triton X100	0.0	0.0	110
WT	D1	herm	paired	Triton X100	9.6	NA	115
WT	D1	herm		Tween20	20.8	NA	120
WT	D1	herm		none	31.0	NA	29
WT	D2	herm	paired	Triton X100	9.4	NA	53
WT	D2	herm		Tween 20	14.6	NA	144
WT	D2	herm		none	25.0	NA	44
*nemp-1(zu501)*	D2	herm	single	Triton X100	4.1	0.0	147
*copa-1(zu482)*	D1	herm	single	Triton X100	0.7	0.0	272
*copa-1(zu482)*	D2	herm	single	Triton X100	0.2	0.0	411
*seip-1(zu483)*	D2	herm	single	Triton X100	6.3	0.9	316

* paired groups were grown synchronously on identical plates, marked after fixation, then mixed and processed together.

To compare nLDs and nuclei in animals of different ages, we grew parallel sets of L4-D4 animals simultaneously and under identical culture conditions. Part of each set was processed for TEM, and the remainder was processed for immunostaining. Quantitative comparisons in this report of nuclei at different stages refer to these parallel sets of animals (Table 2), although all of the basic results were confirmed in additional experiments on separate populations.

**Table 2:** Structures in intestinal nuclei.

sex	stage	structure scored	int1% (n)	subanterior % (n)	posterior % (n)
herm	L4	nLD	0.0 (12)	1.5 (64)	1.9 (53)
		lamin line	16.7 (12)	6.3 (64)	9.4 (53)
		lamin sacs >5	0.0 (12)	0.0 (64)	0.0 (53)
		cysts	0.0 (12)	0.0 (64)	0.0 (53)
	D1	nLD	0.0 (11)	8.3 (72)	5.8 (68)
		lamin line	72.7 (11)	50.0 (72)	44.1 (68)
		lamin sacs >5	0.0 (11)	13.9 (72)	2.9 (68)
		cysts	0.0 (11)	5.5 (72)	2.9 (68)
	D2	nLDs	13.3 (15)	39.7 (73)	6.8 (73)
		lamin line	86.7 (15)	69.6 (73)	72.6 (73)
		lamin sacs >5	6.7 (15)	68.5 (73)	8.2 (73)
		cysts	6.7 (15)	61.6 (73)	10.9 (73)
	D3	nLDs	0.0 (10)	17.6 (51)	5.0 (40)
		lamin line	70.0 (10)	82.3 (51)	80.0 (40)
		lamin sacs >5	10.0 (10)	82.3 (51)	27.5 (40)
		cysts	10.0 (10)	82.3 (51)	17.5 (40)
	D4	nLD	0.0 (13)	7.1 (56)	2.0 (50)
		lamin line	38.4 (13)	83.3 (56)	62.0 (50)
		lamin sacs >5	15.4 (13)	65.0 (56)	16.0 (50)
		cysts	0.0 (13)	48.2 (56)	18.0 (50)

We found that nLDs appeared to be distributed randomly in L4 intestines, but occurred most frequently in the subanterior region of adult intestines (Table 2). For example, nearly 40% of D2 subanterior nuclei contained an nLD, compared with 7–13% of flanking nuclei. The nLDs varied considerably in size, ranging from about 0.3 μm to giant nLDs that were nearly 4 μm, or about 1/3 the diameter of the nucleus (Figs 2C and S2). Some giant nLDs in L4 and D1 nuclei were adjacent to regions of the envelope that appeared deficient in lamin (see below), or were next to apparent gaps or ruptures in the nuclear envelope (Fig 2D and Table 1). The set of L4 intestines examined by TEM contained three examples of nuclear ruptures next to giant nLDs, and in each case both the inner nuclear membrane (INM) and the outer nuclear membrane (ONM) were broken (Fig 2E). Similar ruptures were not observed in immunostained nuclei that lacked nLDs, or that contained only small nLDs; additional features suggested that the ruptures were not simply artifacts of sample preparation (see Legend to Fig 2D).

### Intestinal nLDs are often surrounded by heterochromatin

In overview, our results suggest that nLDs forming at the nuclear envelope have two major developmental trajectories, depending on whether they remain under the nuclear lamina (see below), or breech the lamina and expand beneath the peripheral heterochromatin (this section). Intestinal nuclei, similar to other somatic nuclei, have an inner lining of compacted chromatin (peripheral heterochromatin), as well as small, irregular patches of heterochromatin throughout the nucleoplasm (Fig 2C). Surprisingly, we found that all of the nLDs in L4 nuclei, most nLDs in D1 nuclei, and a variable subset of nLDs in older nuclei, appeared to be coated with heterochromatin (Figs 2C and 2D and S2). Importantly, many of these nLDs were not coated with lamin (Fig 2C and 2D), indicating that they are not simply inpocketings of the nuclear lamina. The nLDs near the nuclear lamina were adjacent to apparent gaps in the peripheral heterochromatin (arrowheads, Fig 2C), suggesting that their heterochromatin coat originated from peripheral heterochromatin. Consistent with this view, all of the giant nLDs at nuclear ruptures had asymmetrical, hemispherical coats of heterochromatin, with the gap at the apparent site of nuclear rupture (Fig 2D and 2E and see Discussion).

By TEM, nLDs closely resembled cLDs in having a homogenous interior of intermediate electron density (Fig 2F), although some variation was observed for both nLDs and cLDs (S2 Fig). By contrast with cLDs, most nLDs were coated with irregular, electron-dense clumps of material that was similar in appearance to heterochromatin (black arrowheads in Fig 2E and 2F, also see S2 Fig). The actual surface of the nLD was largely obscured by the coating, but the surface was visible at occasional gaps in the coat (arrow in panel 4, Fig 2F). At high magnification, the nLD surface was a single, electron-dense line resembling the phospholipid monolayer surface of a cLD (Fig 2G). By contrast, phospholipid bilayer membranes, such as the plasma membrane and both nuclear membranes, have a characteristic "sandwich" appearance consisting of parallel, electron-dense lines (panel 3, Fig 2G). The above TEM features allowed most nLDs to be readily distinguished from other membrane vesicles that occur in intestinal nuclei and that are described below (white arrows, Fig 2F).

By both immunostaining and TEM, the smallest nLDs in L4 and D1 nuclei were close to the nuclear periphery, suggesting an origin at the INM (Fig 2H, quantified in Fig 2I). The small, peripheral nLDs had variable coatings of electron-dense material (Fig 2H), similar to the larger nLDs. In D2 and later nuclei, however, many of the smallest nLDs were not at the nuclear periphery, and instead appeared to be distributed throughout the nuclear interior (Fig 2J, quantified in Fig 2I). Most of the small, interior nLDs appeared to lack heterochromatin coats (insets in Fig 2J), and did not have an electron-dense coating when imaged by TEM (see below). Thus, nLDs in young nuclei likely form at the envelope, displacing peripheral heterochromatin as they grow into the interior, but many nLDs in older nuclei appear to form in the interior.

### nLDs can be associated with nuclear microfilament bundles

**Fig 3:**
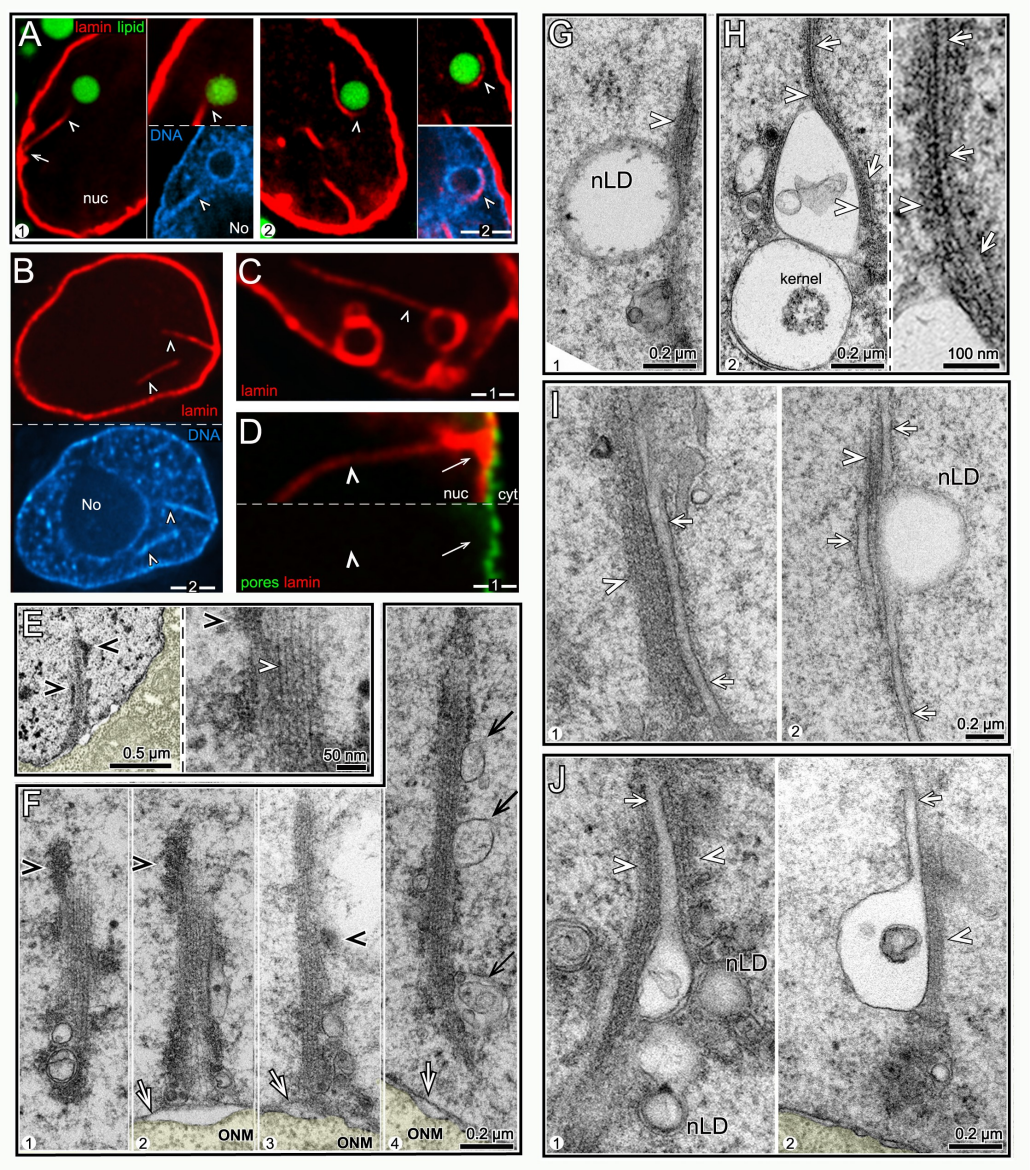
nLDs and nuclear microfilament bundles (nMFBs). (A) Examples of lamin lines (arrowheads; red, LMN-1/lamin) and nLDs (green, BODIPY) in two D1 nuclei. The lamin line in panel 1 extends between the envelope (arrows) and the nLD (top inset). The bottom inset in panel 1 shows that both the nLD and the lamin line are coated with heterochromatin. The nucleus in panel 2 has three lamin lines, one of which partially encircles the nLD; the top inset shows the continuation of the latter lamin line on a different focal plane. (B) D1 nucleus with two lamin lines (arrowheads), both coated with heterochromatin. (C) D2 nucleus with a lamin line (arrowhead) in contact with a lamin-coated vesicle. (D) Lamin line (arrowhead) in a D2 nucleus stained for nuclear pores (green, NPP-9/RanBP2). Note that the line is not surrounded by nuclear pores, suggesting that the line does not represent an invagination of the nuclear envelope. (E) Low magnification TEM of a D3 nucleus showing a linear structure extending from the nuclear membrane into the nucleoplasm. The structure is coated with clumps of electron-dense material (black arrowheads) consistent with the appearance of heterochromatin. The high magnification inset shows that the linear structure is a bundle of parallel microfilaments (nMFB; the white arrowhead indicates a single microfilament). (F) Examples of nMFBs in D1 (panel 1), D2 (panels 2,3) and D3 (panel 4) nuclei. Note that the nMFBs in panels 2 and 3 appear to connect directly to the INM (arrow). The nMFB can be coated with variable clumps of electron-dense material (black arrowheads), or be associated with membranes (black arrows in panel 4). (G) TEM of D2 nucleus showing an nMFB (arrowhead) in contact with a nLD. (H) TEM of D3 nucleus showing an nMFB (arrowhead) in contact with two vesicles and a tubule (arrow and inset). One of the vesicles is a kernel vesicle as described in the text. (I) Examples from D2 nuclei of tubules (arrows) extending parallel to nMFBs (arrowheads). (J) Examples of nMFB-associated tubules (arrows) that appear to be protrusions from membrane vesicles. Note that panel 1 also shows two nLDs that appear to be surrounded by additional membranes, as described below. Scale bars in microns as indicated.

Some of the large nLDs with heterochromatin coats contacted, or were partially wrapped by, linear structures that stained positively for lamin (Fig 3A); for convenience we refer to these nuclear structures as lamin lines. L4 nuclei typically had few or no lamin lines, but the lines were common in older nuclei (Table 2); some individual D3 nuclei contained at least 9 lines (see below). All of the lamin lines were similar in thickness, but varied markedly in length; the longest lines were about 10 μm, or about 2/3 of the nuclear diameter. In most cases, one end of a lamin line could be traced to the nuclear envelope (Fig 3A–3D). Remarkably, many of the lamin lines were surrounded by heterochromatin, particularly in older nuclei (Fig 3A and 3B). For example, heterochromatin was associated with nearly 20% of lamin lines in D2 nuclei, and 51% of the lines in D3 nuclei (n = 76, 80 lines, respectively). In addition to contacting nLDs, the lamin lines often contacted lamin-coated vesicles, or lamin sacs; some of the lamin sacs contained lipid, but other did not (Fig 3C and see below). Intranuclear lines of lamin have been described in other systems and found to be invaginations of the nuclear envelope [43]. However, the lamin lines in intestinal nuclei were not surrounded by nuclear pores, suggesting they are not simply nuclear invaginations (Fig 3D, n = 0/76 lines).

Intestinal nuclei examined by TEM often contained linear structures that closely resembled the lamin lines in size and age-dependence (Fig 3E). At high magnification, the linear structures consisted of bundled, parallel microfilaments that were the thickness expected for F-actin (about 6 nm; inset for Fig 3E). The bundles were not surrounded by a double membrane, indicating that they are not cytoplasmic microfilaments within a nuclear invagination (Fig 3F). Thus, we refer to the bundles as nMFBs (nuclear microfilament bundles). Similar to many lamin lines, the nMFBs often were associated with irregular clumps of electron-dense material resembling heterochromatin (black arrowheads in Fig 3E and 3F). The nMFBs appeared to originate/terminate at the INM (panels 2,3 in Fig 3F), and could contact nLDs (Fig 3G) or other nuclear vesicles as described below (Fig 3H).

### Some nLDs are associated with tubules in a type I nucleoplasmic reticulum

**Fig 4:**
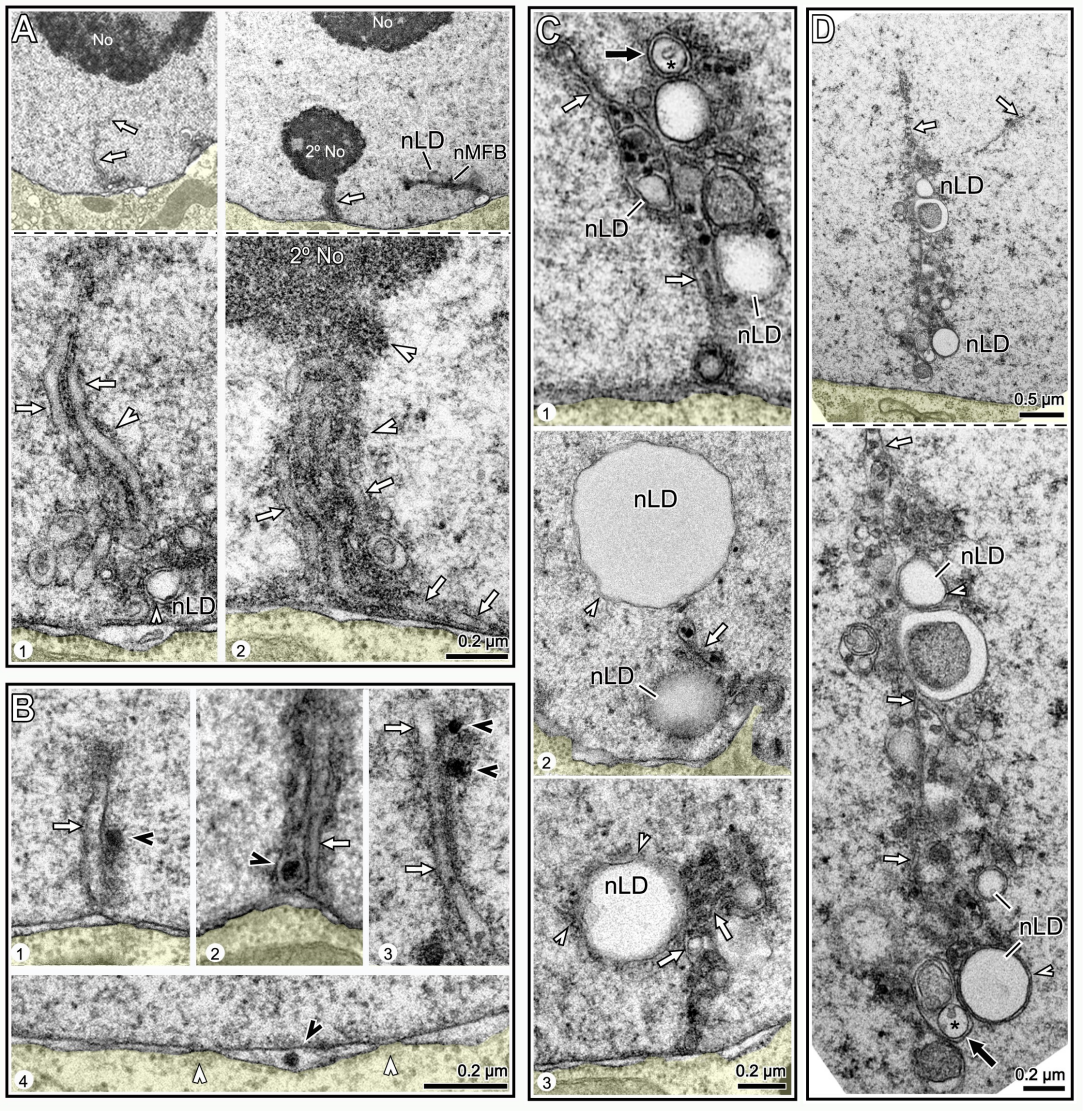
Intestinal nuclei contain a type I nucleoplasmic reticulum. (A) Examples of nuclear tubules (arrows) that appear to extend from the nuclear envelope toward either the nucleolus (panel 1), or a secondary nucleolus (panel 2). Note that these tubules are surrounded by fine granular material (white arrowheads in insets). (B) Examples of nuclear tubules (arrows) that are associated with large, electron-dense granules (black arrowheads). Panel 4 shows an electron-dense granule in the perinuclear cistern between the INM and ONM; white arrowheads indicate nuclear pores (see **S3 Fig** for additional examples). (C) Examples of nuclear tubules (white arrows) associated with nLDs, some of which contain electron-dense granules (see also S3 Fig). White arrowheads in panels 2,3 indicate membrane fragments at the periphery of some nLDs. The black arrow in panel 1 indicates a nested vesicle as described below. (D) TEM of a D3 nucleus at low (top) and high (bottom) magnification showing a linear cluster of tubules (white arrows), nLDs and vesicles. White arrowheads and black arrow as for **Fig 4C**. Scale bars in microns as indicated.

We found several examples of nMFBs that extended parallel to a single-membrane nuclear tubule, or occasionally multiple tubules (arrows, Fig 3H and 3I). The tubules were rare in L4 nuclei, but common in progressively older nuclei, where they were often associated with nLDs and small vesicles (Figs 3I and 3J and S3). Some of the tubules appeared to originate from the INM (Fig 4A), and others appeared to be extensions of, or fused with, membrane vesicles (Figs 3J and S3).

Several types of animal nuclei contain intranuclear or trans-nuclear tubules that collectively are termed a nucleoplasmic reticulum (reviewed in [44]). A type I reticulum consists of single-membrane tubules derived from the INM, and a type II nucleoplasmic reticulum consists of double-membrane tubules formed by the combined invagination of both nuclear membranes. Thus, the tubules in *C*. *elegans* intestinal nuclei appear to represent a type I nucleoplasmic reticulum. The function(s) of a type I or type II nucleoplasmic reticulum are not understood; interestingly, nLDs in liver cells can be associated with a type I reticulum [24,45]. Nuclear tubules in some cell types are associated with nucleoli, including the type I nucleoplasmic reticulum in human endometrium [46]. We found that a few intestinal nuclei contained tubules, or clusters of tubules, that appeared to extend between the envelope and the nucleolus, or a smaller, secondary nucleolus (Figs 4A and S3). The nucleolar-associated tubules were surrounded by fine, granular material that resembled the granular component of the nucleolus, but that was distinct from the electron-dense clumps associated with some nMFBs (Fig 4A). A second class of tubules were adjacent to, or enclosed, large electron-dense granules of about 30–50 nm (Figs 4B and 4C and S3). Granules with a similar appearance could also be found in the perinuclear cistern between the INM and ONM (Fig 4B/panel 4/ and–). nLDs were often in direct contact with, or in close proximity to, both classes of tubules, and many of these nLDs did not appear to be coated with heterochromatin (Fig 4C and 4D; S3 Fig). These results suggest that some of the nLDs in the interior of older nuclei (Fig 2I and 2J) might originate from membranes of type I tubules derived from the INM, rather than from the INM at the nuclear envelope.

### A subset of nLDs appears to form from inpocketings in type I tubules

**Fig 5:**
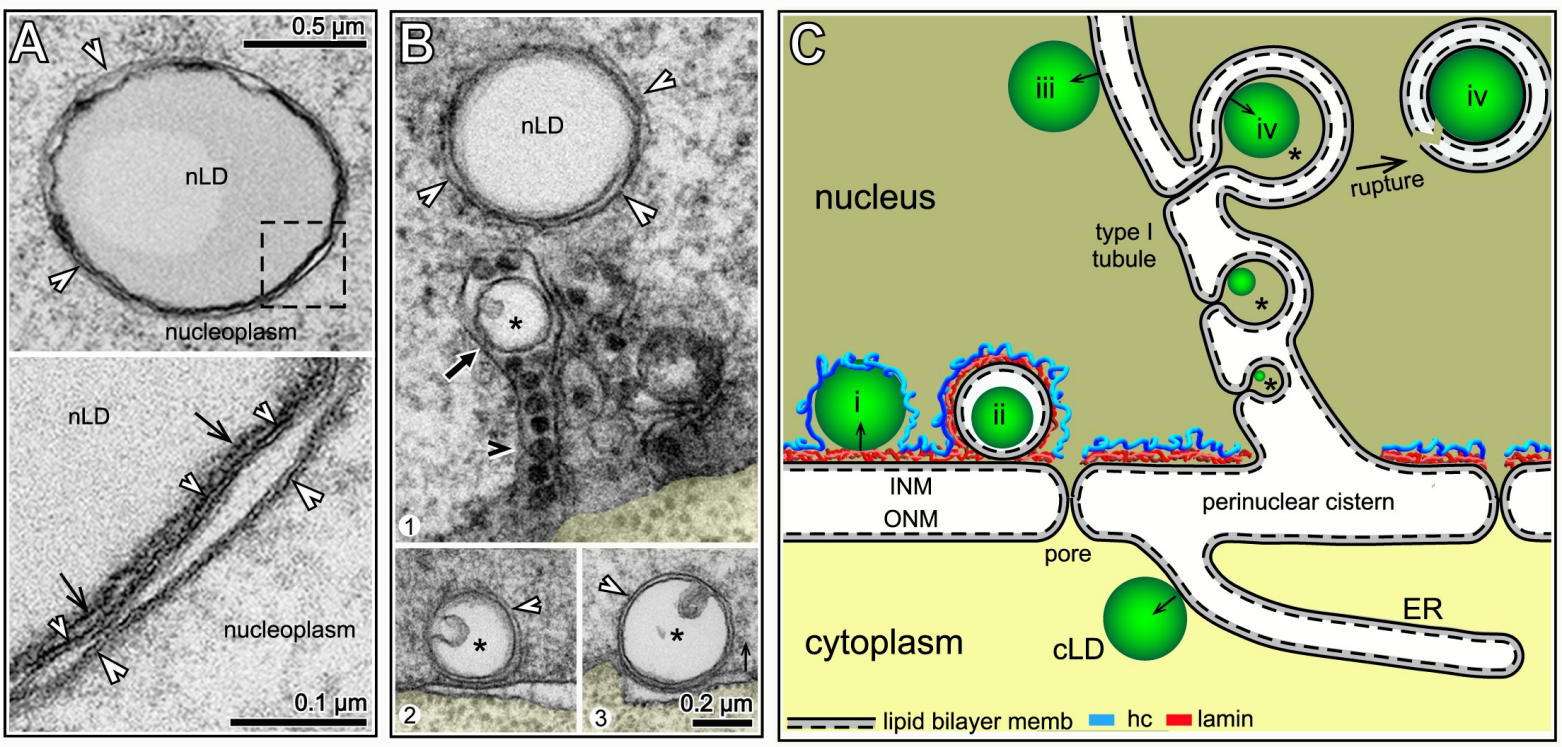
Membrane-enclosed nLDs. (A) TEM of tubule-associated nLD with extra membranes (white arrowheads) in a D2 nucleus; see also **Fig 4C and 4D**. The high magnification inset (bottom) shows that the nLD surface (black arrows) is partially surrounded by two membranes (small and large white arrowheads), both of which have the sandwich appearance of lipid bilayers. (B) Panel 1 shows a tubule-associated nLD with extra membranes (white arrowheads), and a nested vesicle (black arrow) inside a tubule; the tubule contains several electron-dense granules (black arrowhead). The nested vesicles have an internal domain (asterisk) resembling a small lipid droplet; see similar nested vesicles in **Fig 4C and 4D**. Panels 2 and 3 show structures resembling nested vesicles, but located by the nuclear envelope. (C) Summary model for the origin of the different classes of nLDs observed in this study. For comparison, a cLD is shown budding from an ER membrane; note polarity of budding with respect to the two lipid leaflets (solid and dashed lines). Class i nLDs likely form from the INM (see **Fig 2H**) and split the peripheral heterochromatin (blue) from the lamina; class ii nLDs (kernel vesicles, see text) are covered by both lamin and heterochromatin; class iii nLDs bud from INM-derived type I tubules. We propose that class iv nLDs (asterisks) form at an inpocketing of the tubule membrane. A class iv nLD eventually ruptures the tubule, and enters the nucleoplasm with variable fragments of the folded tubule membrane.

Some nLDs in rat hepatocytes expand in the luminal space of a type I tubule, then break free of the tubule membranes to enter the nucleoplasm [45]. We found that many of the nLDs near the type I tubules in intestinal nuclei were surrounded by additional membranes or membrane fragments (white arrowheads in Fig 5A and 5B, see also Fig 4C and 4D). For example, the percentages of nLDs that appeared to be associated with extra membranes were as follows: L4 (0.0%, n = 19); D1 (19.2%, n = 47); D2 (24.4%, n = 41); D3 (40%; n = 25). Surprisingly, nearly all of these nLDs were surrounded by two distinct, lipid bilayer membranes (inset, Fig 5A), rather than a single membrane. A possible origin for the double membranes was suggested by nested vesicles that occur frequently inside tubules (black arrow in Fig 5B, see also black arrows in Fig 4C and 4D), and that occur less frequently by the INM (panels 2 and 3 in Fig 5B). Most of the nested vesicles were not completely filled with lipid, but contained a smaller domain with an electron-density similar to a small lipid droplet (asterisks in Fig 5B, see also Fig 4C and 4D). We propose that a nested vesicle represents an nLD forming within an inpocketing of a tubule membrane (class iv nLD in Fig 5C). In this model, the nLD ruptures from the tubule with remnants of the folded, tubule membrane. Notably, an inpocketing model preserves the same vectorial, budding polarity of a cLD forming from the ER membrane (Fig 5C, see [2] for discussion of vectorial budding of lipid droplets).

In partial summary, one class of nLDs appears to form from the INM at the nuclear envelope, between the lamina and the peripheral heterochromatin. These nLDs expand into the interior with prominent heterochromatin coats. We propose that another class of nLDs develops from type I membrane tubules derived from the INM; because these tubules extend through the lamina and the peripheral heterochromatin, they produce nLDs without heterochromatin coats (Fig 5C).

### A third class of nLDs is surrounded by lamin

**Fig 6:**
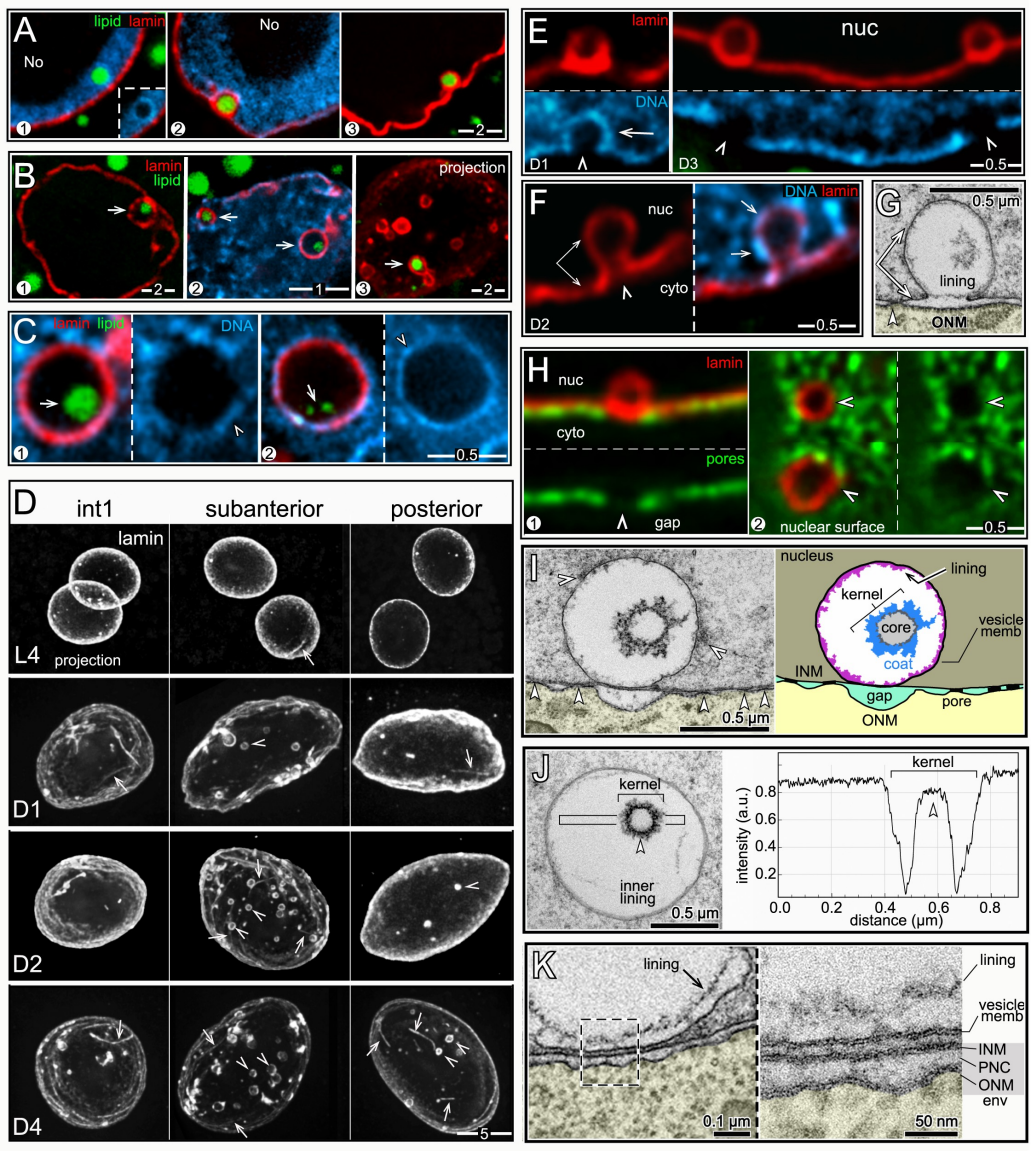
nLDs and lamin sacs. (A) Examples of nLDs with and without lamin coats (red, LMN-1/lamin) in L4 (panel 1) and D1 (panels 2,3) intestinal nuclei. (B) Examples of nLDs that are within, but that do not fill, lamin sacs (arrows). The lamin sacs are closely associated with the nuclear envelope; panels 2 and 3 are tangential planes through the surfaces of D2 and D3 nuclei, respectively. Panel 3 shows a 5 μm maximum intensity z projection to illustrate the number of lamin sacs; note that only one lamin sac appears to contain lipid (arrow). (C) High magnification views of nLDs (arrows) within lamin sacs. The small, internal lipid droplets (arrows) do not appear to be directly surrounded by heterochromatin, although the lamin sac itself has a variable coating of heterochromatin (arrowheads). (D) Intestinal nuclei stained for lamin (white, LMN-1) from different regions of the intestine and at different stages as labeled; the images are maximum intensity projections showing the entire nucleus. Lamin lines (arrows) in the projection are in the nuclear interior, but nearly all of the lamin sacs (arrowheads) are just inside the nuclear envelope. (E) Images of lamin sacs in D1 and D3 nuclei. Lamin sacs in D1 nuclei have distinct coatings of heterochromatin that appear to result from inpocketings of the peripheral heterochromatin. Lamin sacs in D3 and older nuclei typically have relatively little, if any, coating of heterochromatin. (F) D2 nucleus showing a candidate precursor of a lamin sac. The image appears to show an inpocketing of the lamina (double arrow) and peripheral heterochromatin (arrows). (G) TEM of a D2 nucleus showing a candidate precursor of a lamin sac/kernel vesicle. The INM (double arrow) appears to have separated from the ONM to form an inpocketing, and there are no nuclear pores (arrowhead) at the base of the inpocketing. The membrane inpocketing has an irregular inner lining, and clumps of material appear at the outer, nucleoplasmic surface of the inpocketing. (H) D1 intestinal nuclei showing gaps (arrowheads) in the distribution of nuclear pores (green, NPP-9/RanBP2) that are coincident with the positions of lamin sacs. Panel 1 is an optical cross-section of a nucleus, and panel 2 is a tangential plane through the surface of a nucleus. (I) TEM and diagram of a kernel vesicle, or presumptive lamin sac, at a gap between pores. The kernel vesicle appears to surround a small nLD, similar to immunostained images (**Fig 6C**). The membrane surface of a kernel vesicle can be coated with variable clumps of material (white arrowheads in left panel), and nearly always has an irregular, inner lining of material. (J) Intensity scan through a kernel vesicle, showing the increased electron density of the lipid-like core. (K) TEM comparing a kernel vesicle membrane with the adjacent nuclear membranes. The high magnification inset shows that each of these membranes has the typical sandwich appearance of a lipid bilayer. This morphology is consistent with the proposed origin of the vesicle membrane as an inpocketing of the INM (see **Fig 6G**). Scale bars as indicated in microns.

About 30% of the nLDs with heterochromatin coats in L4-D2 nuclei were also enclosed by a sac of lamin (Fig 6A and 6B). The lamin sacs could be filled entirely with lipid (Fig 6A), but often contained a much smaller lipid core, or rarely multiple lipid bodies (Fig 6B and 6C). None of the lipid bodies within lamin sacs had heterochromatin coats, although the sac itself was usually surrounded by heterochromatin (Fig 6C). By contrast with other nLDs in the nucleoplasm, the lamin sacs were nearly always adjacent to the nuclear envelope (see Legend to Fig 6D). The numbers of lamin sacs increased progressively between the D1 and D3 stages, and the sacs were most abundant in subanterior nuclei (Table 2 and arrowheads in Fig 6D). For example, about 80% of D3 subanterior nuclei had 5 or more lamin sacs, but only 10–30% of the flanking intestinal nuclei had similar numbers of sacs. The lamin sacs in older nuclei appeared similar in size and location to those in younger nuclei, but most did not appear to contain lipid (panel 3 in Fig 6B, 5/64 sacs with lipid). Thus, the sacs could either lose lipid over time, or sacs in older nuclei might form independent of lipid. Nearly all of the lamin sacs were at gaps in the peripheral heterochromatin (arrowheads in Fig 6E). The sacs in D1 nuclei typically had a distinct coat of heterochromatin, but most sacs in D3 and D4 nuclei had little or no coating (Fig 6E).

### TEM shows that lamin sacs are membrane vesicles with possible lipid cores

**Fig 7:**
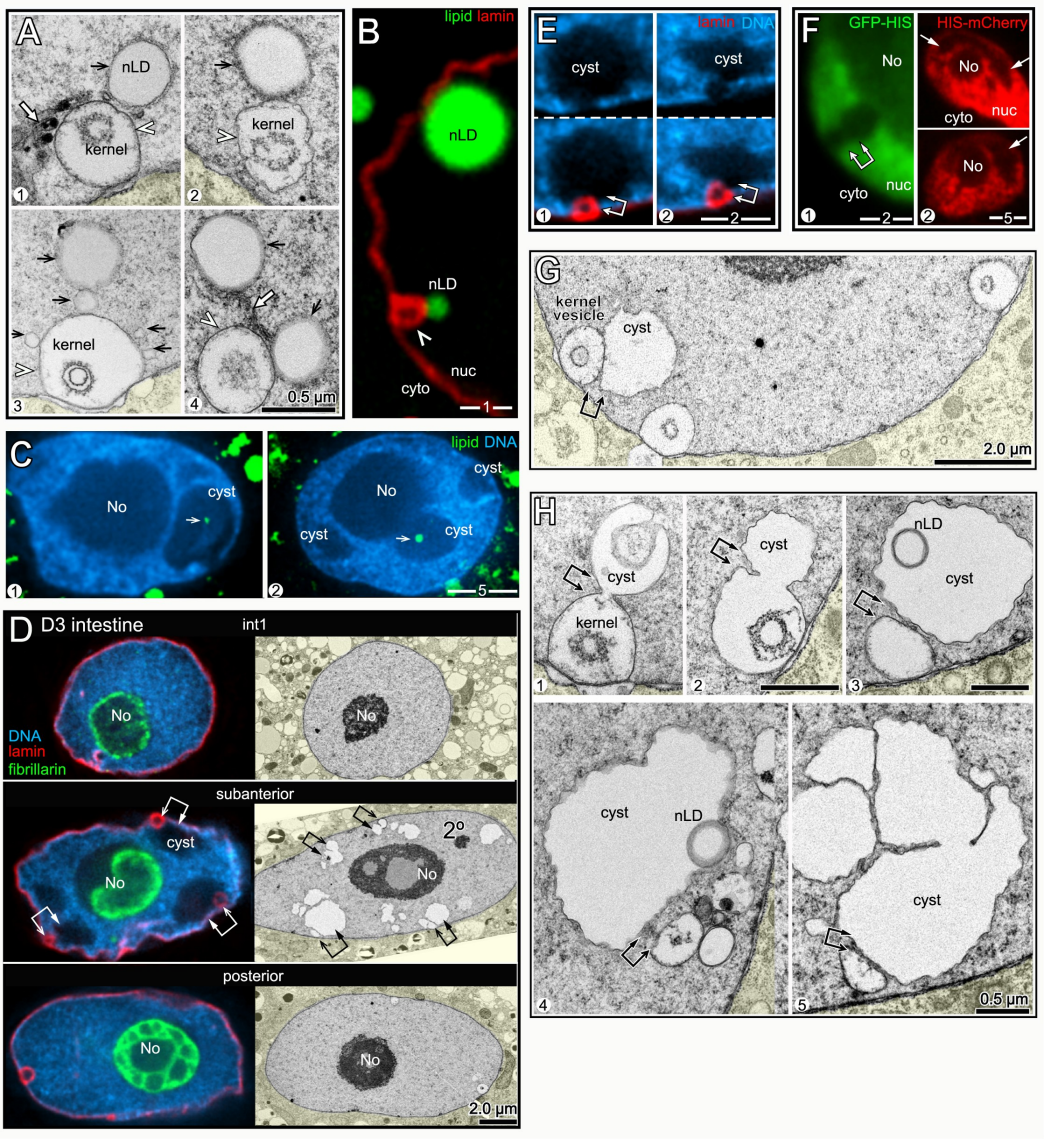
nLDs and nucleoplasmic cysts. (A) TEM images of nLDs (black arrows) at the perimeter of kernel vesicles (arrowheads). Note that there are five nLDs around the kernel vesicle in panel 3. Note also nuclear tubules in panels 1 and 4 (white arrows). (B) D2 nucleus with two nLDs, one at the perimeter of a lamin sac (arrowhead) as in **Fig 7A**. (C) Examples of D3 nuclei with small nLDs (arrows) inside large nucleoplasmic cavities or cysts. (D) Immunostained (left column) and TEM images (right column) of D3 nuclei from the indicated regions of the intestine. Large nucleoplasmic cysts are visible in the subanterior nuclei, but not in the flanking nuclei (see also **S5** and **S6 Figs**). By TEM, the cysts do not resemble secondary nucleoli (labeled in the subanterior nucleus), and do not stain for nucleolar markers (green, anti-fibrillarin). Double arrows in the subanterior nuclei indicate apparent pairings of cysts with lamin sacs/nuclear vesicles. (E) Examples of apparent pairings (double arrows) between cysts and lamin sacs in D3 nuclei. Note that lamin sacs, but not cysts, are adjacent to gaps in the peripheral heterochromatin. (F) Intestinal nuclei in live, D3 worms expressing one of two transgenic, fluorescent reporters for histones. The left panel shows GFP::HIS-2B (green; strain JM149), and the right panel shows HIS-24::mCherry (red; strain RW10062). Both reporters show histone-deficient regions in the nucleoplasm (arrows) consistent with the variable shapes and sizes of cysts or sac/cyst pairs. (G) TEM of D3 nucleus with three kernel vesicles, one of which is paired with a cyst. (H) TEM of D2 (panels 1,2) and D3 (panels 3–5) nuclei showing kernel vesicles paired with cysts. Panels 1 and 2 show kernel vesicle membranes continuous with cyst membranes. The cysts in panels 3 and 4 contain lipid droplets (compare with **Fig 7C**). The cyst in panel 5 has a cauliflower shape and appears to be fragmented.

Images of possible nascent sacs in D1 and D2 nuclei appeared to show inpocketings of both the lamina and the peripheral heterochromatin (Fig 6F). Similar inpocketings observed by TEM showed a separation of the INM from the ONM (double-headed arrow in Fig 6G). The separation between the INM and ONM would be incompatible with the structure of a nuclear pores (Fig 5C); accordingly, we found that nearly all immunostained lamin sacs were beside circular gaps in the otherwise uniform distribution of nuclear pores (Fig 6H): When the positions of lamin sacs and the gaps between pores were scored separately and then compared, 102/110 lamin sacs were at gaps, and 80/93 gaps were adjacent to lamin sacs. This nearly invariant correspondence, combined with the sizes and age distribution of the sacs, allowed us to identify fully-formed sacs as distinct membrane vesicles by TEM (Fig 6I). The vast majority of these vesicles contained a smaller inner structure we term the kernel (Figs 6I and 6J and S4). The kernel consists of a spherical, homogenous core of intermediate electron density similar to nLDs, and the core is surrounded by a variable, non-membranous coat (Fig 6J). For clarity, we refer to TEM images of the presumptive lamin sacs as kernel vesicles, and use the term lamin sacs for immunostained nuclei. Similar to lamin sacs, the kernel vesicles were spherical, about 0.5–1.0 μm in diameter, absent in L4 nuclei, juxtaposed to the nuclear envelope, and most abundant in subanterior nuclei. The kernel vesicles were surrounded by a lipid bilayer membrane (right panel, Fig 6K). The outer (nucleoplasmic) face of the vesicle membrane was often associated with variable clumps of electron-dense material, and the inner face typically had a loosely-associated lining of material (Fig 6G and 6I–6K). In addition to having possible lipid cores, a few kernel vesicles had an nLD, or occasionally multiple nLDs, at their perimeter (Fig 7A), as did immunostained lamin sacs (Fig 7B). Thus, nLDs might form infrequently from the INM-derived membrane surrounding a lamin sac/kernel vesicle.

### Nuclear damage associated with lamin sacs/kernel vesicles

A small subset of nLDs were within large, chromatin-deficient regions of the nucleoplasm that we term cysts (Fig 7C). The cysts were not present in L4 or most D1 intestines, but occurred frequently after the D2 stage where they were most abundant in the subanterior region (Fig 7D and 7E and Table 2, see also S5 and S6 Figs). The cysts did not stain with antibodies that recognize nucleoli, nor did the cysts resemble nucleoli or secondary nucleoli by TEM (Fig 7D). Structures resembling cysts were visible in live, D3 nuclei expressing either of two different fluorescent histone reporters (Fig 7F), suggesting that the cysts are not artifacts of sample preparation.

Remarkably, nearly all of the cysts appeared to be paired with a lamin sac (double arrows in Fig 7D and 7E). Similarly, TEM images showed that the cysts were paired with kernel vesicles, many of which appeared to have partially degraded cores (double arrows in Fig 7D, 7G and 7H). The cysts were enclosed by a lipid bilayer membrane, and in many images this membrane was continuous with the membrane of a kernel vesicle (panels 1–2 in Fig 7H). Some of the cysts contained what appeared to be lipid droplets (panels 3–4 in Fig 7H), consistent with staining experiments that showed lipid droplets in cysts (Fig 7C). These observations suggest that cysts form as herniations of degraded lamin sacs/kernel vesicles in older nuclei, and acquire nLDs indirectly from those lamin sacs/kernel vesicles.

### Intestinal nuclei appear to remove nLD-associated debris

**Fig 8:**
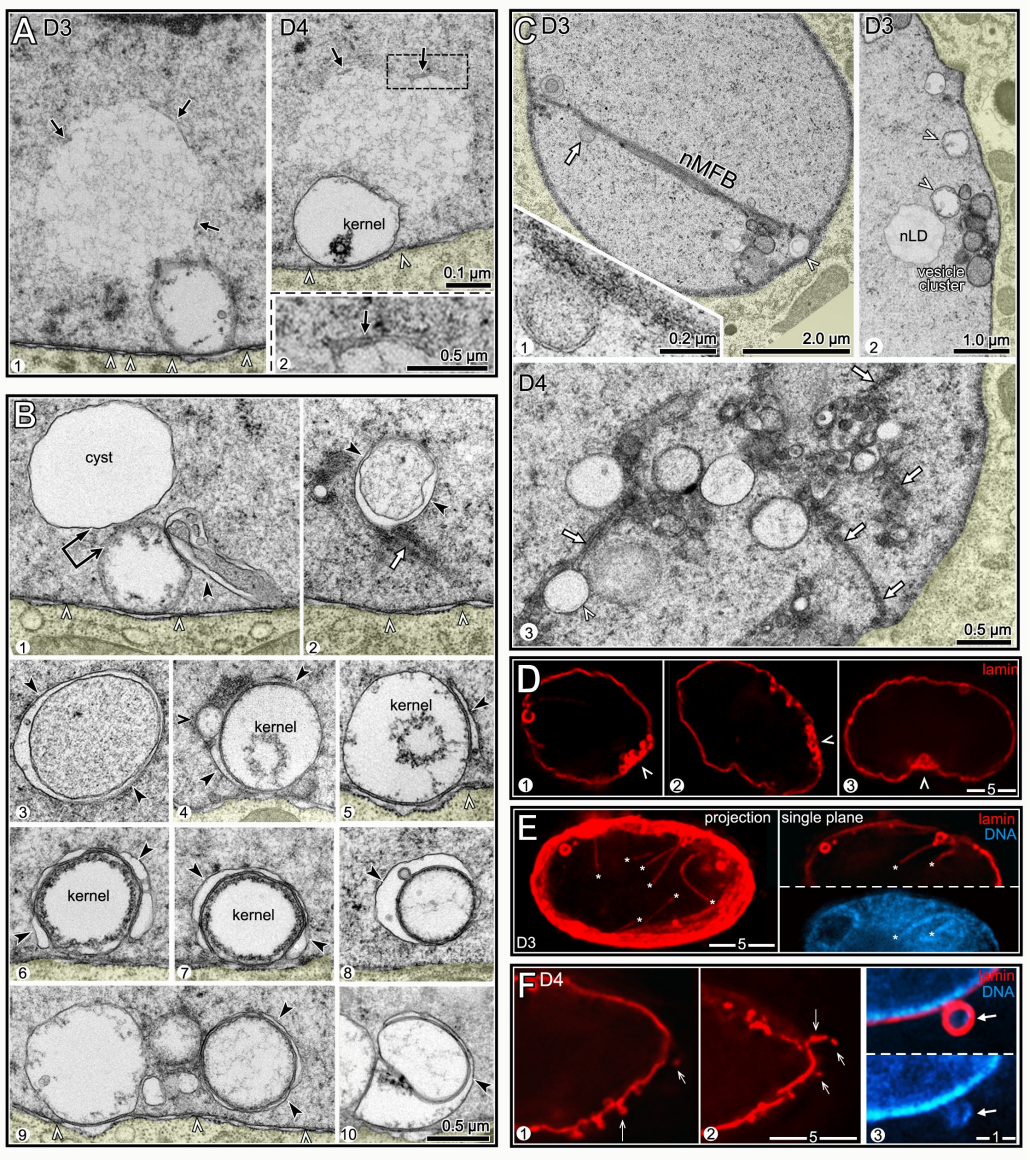
Degradation/engulfment of nuclear vesicles and nLDs. (A) TEM of D3 and D4 nuclei. Each nucleus contains a large region of atypical nucleoplasm that is partially surrounded by membrane fragments and that is adjacent to a membrane vesicle. The vesicles are identified as degraded kernel vesicles by their size, position, and by the gaps between nuclear pores (arrowheads) at the bases of the vesicles. (B) TEM images of D3 and D4 nuclei showing engulfment of various nuclear structures; the black arrowheads indicate candidate engulfing membranes. Panel 1 shows a membranous projection contacting a degraded kernel vesicle. Panels 2 and 3 show membrane-engulfed nucleoplasm; note the membrane tubule (arrow) in panel 2. Panels 4–8 show apparent engulfment of kernel vesicles or degraded kernel vesicles. Panels 9–10 show engulfed vesicles of undetermined identity. (C) TEM of D3 and D4 nuclei with vesicle clusters. Panel 1 shows a long nMFB associated with a large cluster of vesicles; the nMFB contacts what appears to be engulfed, aberrant nucleoplasm (white arrow and inset). Panels 2 and 3 show vesicle clusters that include nLDs, degraded kernel vesicles (white arrowheads), membrane tubules (white arrows), and several uncharacterized vesicles or membrane-engulfed nucleoplasm. (D) D3 nuclei with clusters of lamin sacs (red, LMN-1) at flattened or indented regions of the envelope (arrowheads). (E**)** D4 nucleus with at least 7 lamin lines (asterisks). The left panel is a 3 μm maximum intensity z-projection and the right panel is a single optical plane showing two lamin lines, both surrounded by heterochromatin. (F) D4 nucleus with protruding or detached, lamin-containing structures (arrows). Panel 3 is a higher magnification showing DAPI-staining material in one protrusion. Compare with TEM images in **S4 Fig**.

D3 and D4 nuclei, but not younger nuclei, often contained regions of aberrant nucleoplasm that were comparable in size and shape to cysts, and that were adjacent to degraded kernel vesicles (Fig 8A). However, the aberrant nucleoplasm was bordered by variable membrane fragments, rather than a continuous membrane (arrows, Fig 8A and inset). These features suggest that the aberrant nucleoplasmic regions represent lysed cysts. D3 and D4 nuclei contained several examples of what appeared to be membrane vesicles or protrusions that engulfed aberrant nucleoplasm, degraded kernel vesicles, and unidentified structures (black arrowheads in Fig 8B). Engulfed materials and vesicles were often in clusters that contained nMFBs and tubules (Fig 8C). Similarly, immunostained D3 and D4 nuclei contained clusters of nLDs, lamin sacs and other, much smaller bodies that were covered with lamin; these clusters typically occurred at concavities or flattened regions of the nucleus (arrowheads, Fig 8D). For example, 60.7% of D4 subanterior nuclei contained clusters with five or more lamin sacs (n = 56 nuclei). The percentages of D3 and D4 nuclei with at least one lamin line/nMFB were only moderately higher than for D2 nuclei (Table 2). However, individual D3 and D4 nuclei could have many more lamin lines than younger nuclei, and nearly all of the lines in older nuclei were coated with heterochromatin (Fig 8E). Finally, several D3 and D4 nuclei had variable, lamin-containing structures that projected, or were detached, from the nucleus, and a few of these structures appeared to contain DNA (panel 3 in Fig 8F). Similarly, TEM showed that D3 and D4 nuclei could have protrusions of the ONM that contained apparent remnants of membranous structures and other material (S4 Fig).

The above results suggest that hermaphrodite intestinal nuclei accumulate damage by the end of the self-fertile period, and try to repair/remove this damage. Much of this damage appears to be associated with degraded lamin sacs/kernel vesicles, at least some of which once contained lipid. Finally, the nuclear damage occurs most frequently in the subanterior region of the intestine, which experiences the largest changes in fat and has the highest frequency of nLDs.

### Male intestines have stable levels of fat, few nLDs, and little nuclear damage

**Fig 9:**
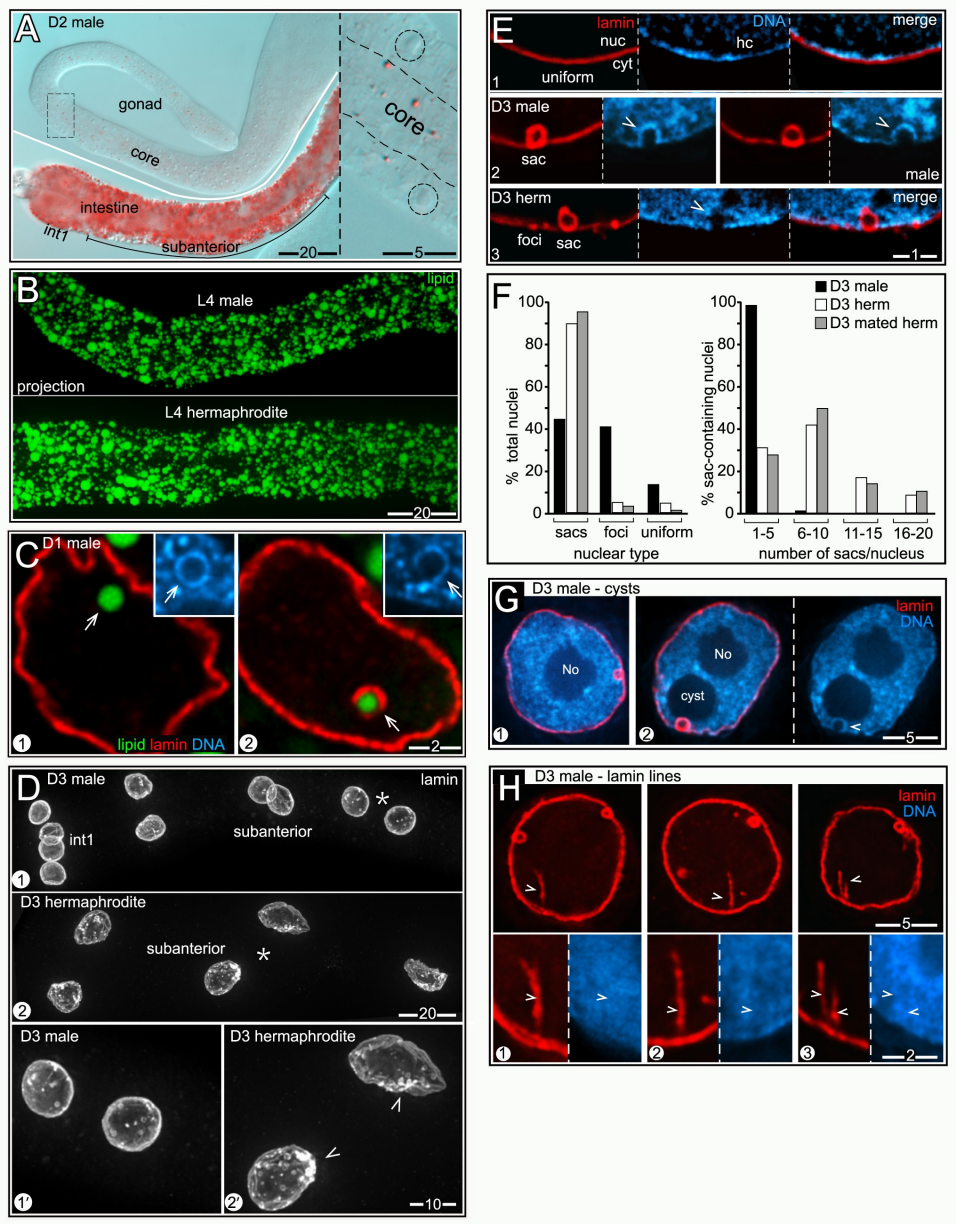
Male nuclei have few nLDs and appear healthier than hermaphrodite nuclei. (A) Fat (red, oil red O) in a D2 male intestine and gonad; the inset shows the boxed region of the gonad at higher magnification with germ nuclei outlined. (B) Comparison of fat (green, BODIPY) in L4 male and L4 hermaphrodite intestines. Images are 8 μm maximum intensity z-projections. (C) nLDs (arrows) in D1 male nuclei; both have heterochromatin coats (insets), but only one has a lamin coat. (D) Nuclei in D3 male and hermaphrodite intestines stained for lamin (white, LMN-1). Panels 1’ and 2’ show higher magnifications of the subanterior nuclei indicated by asterisks in panels 1 and 2, respectively. All images are maximum intensity z-projections representing entire nuclei. Note that the male nuclei are relatively small and round and have fewer lamin sacs. Arrowheads indicate clusters of lamin sacs in the hermaphrodite nuclei. (E) Types of nuclear lamina in D3 intestinal cells, stained for lamin (red, LMN-1). The lamina can appear uniform (panel 1), contain lamin sacs (panel 2), or have foci with or without sacs (panel 3); quantified in **Fig 9F**. Lamin sacs in D3 male nuclei nearly always have distinct heterochromatin coats (arrowheads in panel 2), similar to younger hermaphrodite nuclei at D1 and D2. However, lamin sacs in D3 hermaphrodite nuclei usually lack distinct heterochromatin coats (arrowhead in panel 3; see also **Fig 6E**). (F) Quantification of nuclear types in D3 males and hermaphrodites. The numbers of nuclei scored were as follows: D3 males (784), D3 hermaphrodites (146), mated D3 hermaphrodites (215). (G) Nucleoplasmic cysts in D3 male nuclei. Male nuclei typically lack cysts (panel 1), or have only one cyst (panel 2). When present, the cysts are always associated with lamin sacs (panel 2). The arrowhead indicates the heterochromatin coat around the sac. (H) D3 male nuclei with lamin lines (arrowheads). The high magnification insets at bottom show that the lines have little apparent association with heterochromatin, by contrast with lamin lines in D3 and D4 hermaphrodites. Percentages of nuclei with lamin lines were quantified by mixing fixed D3 male and D3 hermaphrodite intestines prior to permeabilization and immunostaining; 37.4% of the male nuclei (n = 374) had at least one lamin line compared to 70.7% (n = 215) of hermaphrodite nuclei. At younger stages, the following male nuclei had lamin lines: L4 (1.5%, n = 99), D1 (18.8%, n = 80).

Although a detailed examination of males was beyond the scope of this study, we wanted to know whether nuclei in male intestines undergo the types of changes observed in hermaphrodites. Male gonads grow considerably after the L4 stage, but have much less fat than hermaphrodite gonads (Fig 9A, compare Fig 1A). Thus, male gonads might require fewer intestinal resources than in hermaphrodites, and male intestines do not produce yolk lipoproteins. We found that L4 male intestines were slightly smaller than L4 hermaphrodite intestines, but appeared to have a similar density of cLDs (Fig 9B). By contrast with hermaphrodites, adult male intestines maintained a uniform distribution of fat from D1 to D4 (Fig 1G). nLDs occurred in male nuclei and were coated with lamin and/or heterochromatin (Fig 9C). However, nLDs were much less common than in hermaphrodites at the same stages (Table 1). None of the male nuclei had nLDs larger than about 1μm, and none of the male nuclei appeared ruptured (Table 1).

Male nuclei in general resembled younger hermaphrodite nuclei. For example, D3 male nuclei were smaller and rounder than D3 hermaphrodite nuclei (Fig 9D). The male nuclei maintained a relatively uniform lining of lamin, with fewer lamin sacs (Fig 9D and 9E, quantified in Fig 9F), and lacked the flattened or concave regions that are associated with clusters of lamin sacs in hermaphrodites (arrowheads in Fig 9D). Nearly all of the lamin sacs in D3 male nuclei had heterochromatin coats (panel 2 in Fig 9E), which are usually not apparent by the D3 stage in hermaphrodites (panel 3 in Fig 9E). Cysts were present in relatively few male nuclei, but were always paired with lamin sacs (Fig 9G). Finally, lamin lines were present in male nuclei, as in hermaphrodites (Fig 9H). However, the lamin lines in D3 males had little or no apparent association with heterochromatin (Fig 9H), by contrast with the lines in D3 hermaphrodites (Fig 8E). We conclude that male nuclei experience a high, but possibly stable, environment of cytoplasmic fat, and appear to accumulate less damage than subanterior nuclei in hermaphrodites.

### nLDs occur in adult hermaphrodite germ cells and differ from intestinal nLDs

**Fig 10:**
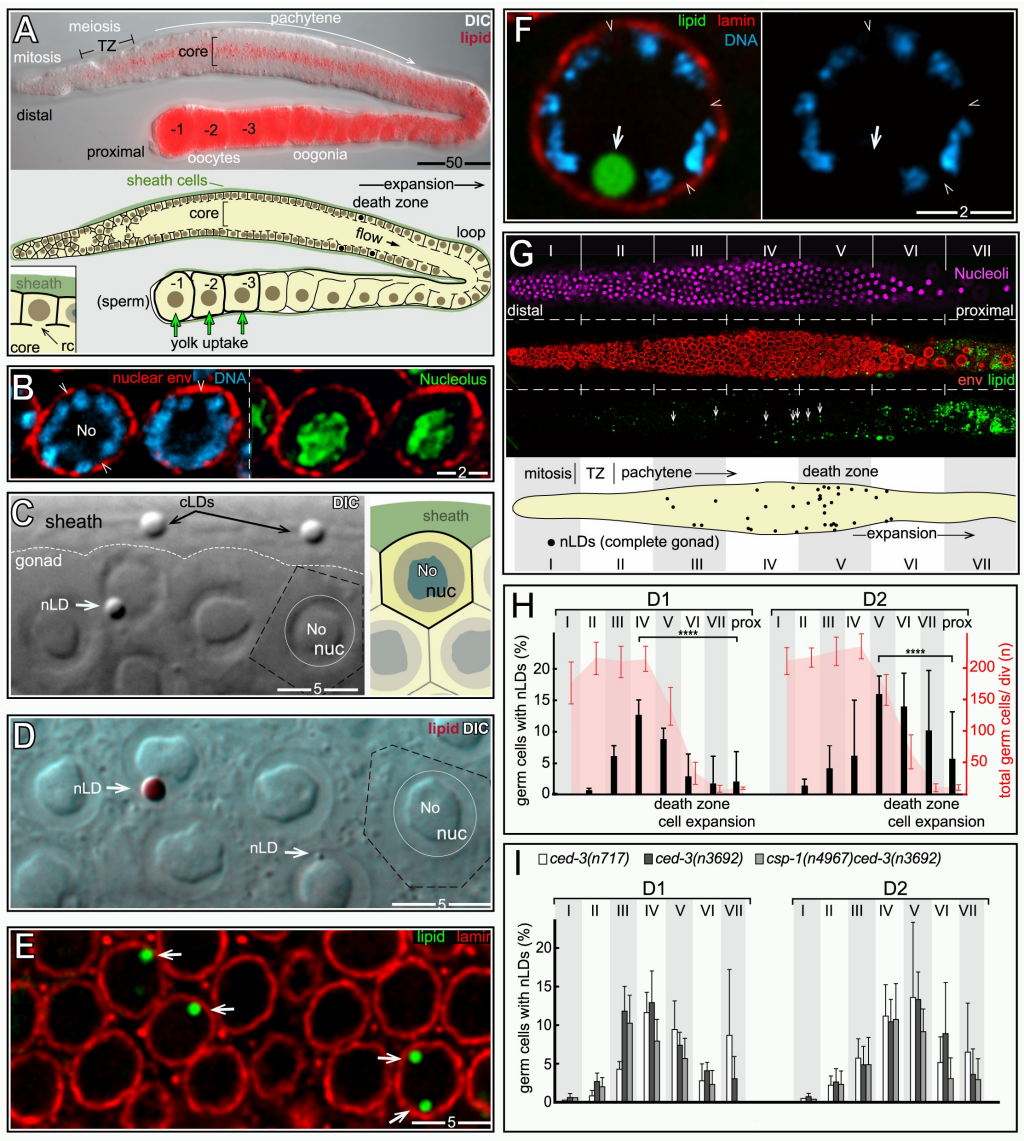
nLDs in germ cells. (A**)** D2 hermaphrodite gonad stained for lipid (oil red O) and imaged with DIC optics. Germ cells exit the mitotic region and enter meiosis in the transition zone (TZ), consisting of leptotene- and zygotene-stage nuclei. (B) Germ nuclei showing pachytene chromosomes (blue, DAPI) surrounding the large, central nucleolus (No; green, fibrillarin). Germ nuclei appear to lack a continuous lining of peripheral heterochromatin (arrowheads). Nuclear pores in germ cells are concentrated in irregular patches, resulting in non-uniform staining at the envelope. (C) DIC image of the pachytene region in a live, D1 hermaphrodite gonad. The white, dashed line indicates the boundary between the germ cells and a sheath cell. Two cLDs are visible in the sheath cell, as identified by their characteristic size, shape, and refractility. A similar body appears in one of the germ nuclei (arrow), but the germ cell cytoplasm appears otherwise devoid of cLDs. The diagram at right illustrates germ cell boundaries in a similar focal plane. (D) DIC image of gonad stained for lipid (oil red O), with arrows indicating large and small nLDs. (E) D1 pachytene germ cells stained for lipid (green, BODIPY) and for the nuclear lamina (red, LMN-1/lamin). Three germ nuclei contain nLDs (arrows), including one with two nLDs. (F) High magnification of a D1 pachytene germ nucleus with an nLD. The nLD does not appear to be coated with heterochromatin or lamin (arrow). Note that the nuclear envelope is not lined with peripheral heterochromatin (arrowheads). Compare with D1 intestinal nuclei in **Fig 2C**. (G) Example of an immunostained D1 gonad used to quantify nLD frequency. Nucleoli (magenta, fibrillarin) were used for automated nuclear counts for every 50 micron division of the gonad (I-VII). The nuclear envelope (red, NPP-9/RanBP2) and lipid (green, BODIPY) channels were used to distinguish nLDs (arrows) from cLDs. This analysis was repeated for each z-layer through the gonad; the bottom diagram is a summation of all confirmed nLDs in this gonad. (H) Combined percentages of germ cells with nLDs in D1 and D2 gonads, 10 gonads analyzed as in **Fig 10G** per stage. The earliest nLDs occur in division II, where meiotic cells enter pachytene. The large variation in nLDs near the loop of the gonad (after division 7) results from the low numbers of germ cells per division, and the rare occurrence of nLDs in this region. (I) Plot as in **Fig 10H**, but showing apoptosis-defective mutant strains as listed. Error bars indicate standard deviations for each division. Unpaired two-tailed T-tests were performed; ****P<0.0001. Scale bars in microns as indicated.

We next examined fat in germ cells. The adult hermaphrodite gonad consists of two identical halves, or arms (Fig 10A), with a combined size comparable to the intestine. However, the gonad contains nearly 2400 germ nuclei, compared to the 30–34 intestinal nuclei [47]. Germ cells divide mitotically in a niche at the distal end of the gonad, and enter meiosis as they exit the niche (Fig 10A, for general reviews see [48–50]). Meiotic germ cells have an extended pachytene stage that occupies most of the gonad, and that can be subdivided into early-, mid-, and late-pachytene stages. Pachytene cells are organized in a cylindrical array around a large, shared cytoplasmic region called the core; each cell has a small opening, the ring channel, which connects it with the core (inset, Fig 10A). Most images here show either sagittal planes through the gonad (Fig 10A), or tangential planes through the uppermost level of nuclei.

Pachytene nuclei are about 1/3 the size of adult intestinal nuclei, with a large nucleolus that occupies most of the nuclear volume (Fig 10B). The paired, highly compacted pachytene chromosomes appear to have variable contact with the nuclear envelope, but germ nuclei lack the continuous layer of peripheral heterochromatin found in intestinal nuclei (arrowheads, Fig 10B). Between the L4 and adult stages, the hermaphrodite gonad accumulates a high level of fat that is comparable to the level of fat in the intestine (Figs 1C and 10A). Some of the gonad fat is in the form of yolk lipoproteins that are synthesized and secreted by the intestine, then taken up in the gonad by receptor-mediated endocytosis [51]. Yolk uptake occurs primarily in the three most mature oocytes, and thus does not account for most of the fat in the gonad (Fig 10A). Fatty acids, or derivatives thereof, also are transferred to the gonad from somatic tissues [52]. For example, malonyl-CoA, the rate-limiting substrate for fatty acid synthesis, is transferred into germ cells through gap junctions with somatic sheath cells that cover most of the gonad (Fig 10A) [53–55]. cLDs and other cytoplasmic materials in the gonad core flow toward and into expanding oocytes at the proximal end of the gonad; core materials travel from the mid-pachytene region into an oocyte in about 45 minutes during the peak egg-laying period [56]. Thus, the high, steady-state level of gonad fat must constantly be replenished as fat-rich eggs are laid.

**Fig 11:**
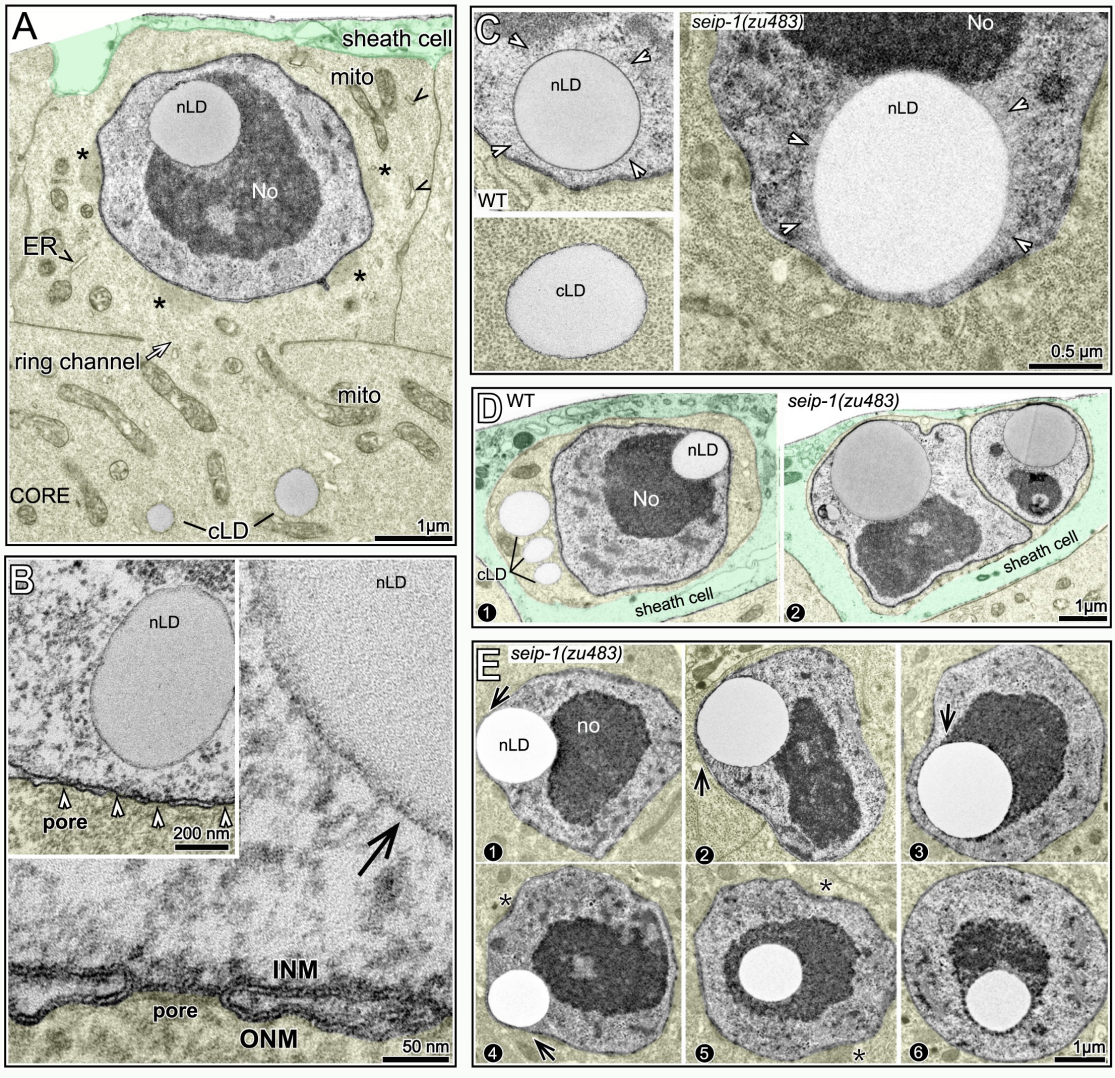
TEM of germ cell nLDs. (A) TEM of a D1 gonad showing a pachytene germ cell with an nLD; the germ cell cytoplasm is tinted yellow, except for cLDs, and a gonad sheath cell is tinted green. The nucleus is approximately spherical, except for small, flattened regions by perinuclear, germ cell-specific granules called P granules (asterisks). The germ cell cytoplasm contains mitochondria (mito) but few ER profiles (arrowheads, see also S8 Fig). For this analysis, we dissected the peak nLD zone from D1 and D2 gonads as described for **Fig 10H**. 62/347 pachytene germ cells from this zone contained nLDs; most nLDs appeared to be about 0.5 μm in diameter, but a few were over 1 μm. Of 55 non-apoptotic cells with nLDs, 51 had a single nLD, 3 had two nLDs, and 1 had three nLDs. (B) Comparison of the surface of an nLD (black arrow) with the lipid bilayer membranes of the INM and ONM in the same nucleus. Note that the nLD directly abuts nucleoplasmic materials, with no evidence of a heterochromatin coat (compare with intestinal nLDs in **Fig 2F**). (C) TEM of nLDs with bristle coats (arrowheads) in wild-type and *seip-1(zu483)* mutant germ cells. The bristle coat separates the nLD from other nucleoplasmic materials, and is not present on cLDs. (D) Images of nLDs in apoptotic germ cells in wild-type and *seip-1(zu483*) gonads. The apoptotic cells are shrunken and engulfed by phagocytic sheath cells (green tint). The wild-type cell has three cLDs, presumably because it had begun to fill with core cytoplasm before shrinkage. Note that the *seip-1* mutant cell is binucleate; binucleate cells occur in wild type gonads and are removed by apoptosis. (E) Pachytene germ nuclei with large nLDs. Note bulges (arrows in panels 1–4) where nLDs abut the envelope. Nuclei with central nLDs are spherical, expect for small indentations at the site of P granules (asterisks, see also **Fig 11A**).

We found that nLDs were present in a small subset of pachytene germ cells in all adult hermaphrodite gonads (n = 134 D1 gonads and 115 D2 gonads). nLDs in live animals resembled highly refractile spheres that were similar to cLDs, but easily distinguished from other cytoplasmic constituents (Fig 10C). The nLDs stained with the lipid dyes oil red O, BODIPY, LipidTOX, and Nile Red (Figs 10D and 10E and S7), and were entirely within the nuclear envelope by the same criteria used for intestinal nLDs (S7 Fig). By TEM, germ cell nLDs resembled cLDs in the gonad (Fig 11A). For example, the germ cell nLDs and cLDs were both surrounded by a single, poorly defined electron-dense line, by contrast with the double line or sandwich appearance of both the INM and ONM (Fig 11B). However, nLDs in germ cells and intestinal cells differed in some respects. First, none of the germ cell nLDs appeared to be coated with heterochromatin or lamin when examined by TEM (Fig 11B) or by staining (Fig 10E and 10F). Second, about half of the germ cell nLDs had a distinctive surface layer of about 100 nm that excluded other nucleoplasmic materials, and that consisted of fine, radiating fibrils or bristles (27/52 nLDs; Fig 11C). This coating was noteworthy as previous studies described a morphologically similar bristle coat on a subset of nLDs in human hepatocytes [24]. Finally, all of the smallest nLDs in germ cells were adjacent to the nuclear envelope, and no germ cell nLDs were associated with extra membranes. D1-D4 germ nuclei lacked other notable features of intestinal nuclei, including lamin lines, lamin sacs, cysts, and membrane tubules, and none of the germ nuclei appeared ruptured (n>1000 germ nuclei analyzed).

### nLDs disappear in the major apoptotic region, but independent of apoptosis

To characterize the distribution of germ cell nLDs, we stained gonads for lipid, the nuclear envelope, and for nucleoli. Optical z-stacks were acquired through the entire gonad, and scored in 50 μm divisions from distal to proximal (Fig 10G); automated counts of nucleoli were used as a proxy for the total number of germ cells, and nLDs were confirmed by analysis of the nuclear envelope. The positions of the first germ cells with nLDs varied between gonads, but the earliest examples were meiotic cells at early pachytene (division II in Fig 10H). Because nLDs form before germ cells begin to take up yolk lipoproteins (Fig 10A), we did not expect nLD formation to depend on yolk. Indeed, *rme-2(b1008)* mutants which fail to take up yolk [51] appeared to have normal numbers of nLDs (n = 24 gonads, compare Fig 10E with S7 Fig). The percentages of D1 and D2 germ cells with nLDs increased to a peak of about 13–15% during pachytene, then declined in later meiotic stages (Fig 10H).

The post-peak decline in nLDs occurs in the major apoptotic region of the gonad, where the surviving germ cells increase in size and intercalate into a single row of oocytes (Fig 10G and 10A). Because our TEM analysis showed that apoptotic cells can have nLDs (Fig 11D), we addressed whether the post-peak decline resulted from apoptosis of nLD-containing germ cells. The caspases CED-3 and CSP-1 are both expressed in the gonad, although only CED-3 is essential for germ cell apoptosis [57]. We found that the distribution profile of nLDs in *ced-3* mutants and in *csp-1;ced-3* double mutants was similar to wild type, with a distinct peak followed by a decrease in nLDs (Fig 10I). Thus, these experiments show that nLDs disappear independent of apoptosis, presumably by lipolysis. However, they do not address whether germ cells with nLDs could be preferentially targeted for apoptosis in wild-type gonads.

### nLD formation is associated with rapid oogenesis

Although nLDs were present in all adult hermaphrodite gonads, which produce oocytes, nLDs were not detected in the vast majority of L4 gonads, which produce sperm (Table 3). Similarly, nLDs were not detected in the vast majority of D1 and D2 male gonads (Table 3). To determine whether hermaphrodite spermatogenesis, which precedes oogenesis, had a role in nLD formation, we examined *fog-2(q71)* mutant hermaphrodites that produce oocytes but never produce sperm [58]. *fog-2* mutant D1 adults had about the same percentage of germ cells with nLDs as wild type (peak zone 11.3% versus 12.1%, respectively; Table 3). However, *fog-2* D2 adults had a much lower percentage of nLDs than wild type (4.6% versus 15.1%; Table 3). Signals from sperm are known to stimulate oogenesis, and would be present in D2 wild-type hermaphrodites but absent in unmated, D2 *fog-2* females [59,60]. Thus, we examined mated *fog-2* mutants at D2, and found they had a similar percentage of nLDs as wild type (18.9% versus 15.1% respectively; Table 3). Thus, nLD formation is associated with oogenesis irrespective of prior spermatogenesis. However, sperm stimulates nLD formation in older adults, likely by stimulating oogenesis. We next examined the effect of low culture temperature on nLD formation, as low temperatures slow oogenesis and development in general. Indeed, shifting the culture temperature from 20°C to 15°C significantly decreased both the numbers and sizes of germ cell nLDs in D1 and D2 gonads (S7 Fig).

**Table 3:** Germ cell nLDs.

genotype	stage	sex	gametes produced*	germ cells with nLDs in the peak zone % (n)	gonads analyzed
WT	D1	male	sp	0.05 (2182)	37
WT	D2	male	sp	0.07 (6034)	48
WT	L4	herm	sp	0.02 (5167)	34
WT	D1	herm	sp/ooc	12.1 +/- 2.0 (1432)	134
WT	D2	herm	sp/ooc	15.1 +/- 2.8 (1211)	115
WT 15°C	D1	herm	sp/ooc	1.1 +/- 0.7 (1400)	7
WT 15°C	D2	herm	sp/ooc	1.6 +/- 0.8 (1600)	8
WT 25°C	D1	herm	sp/ooc	14.2 +/- 6.7 (1400)	7
WT 25°C	D2	herm	sp/ooc	16.0 +/- 5.0 (1800)	9
*fog-2(q71)*	D1	female	ooc	11.3 +/- 2.2 (807)	8
*fog-2(q71)*	D2	female	ooc	4.6 +/- 3.3 (1273)	9
*fog-2(q71)*	D1	mated female	ooc	13.1 +/- 4.0 (1229)	9
*fog-2(q71)*	D2	mated female	ooc	18.9 +/- 9.9 (1291)	10
*seip-1(zu483)*	D1	male	sp	0 (2996)	18
*seip-1(zu483)*	D2	male	sp	0.05 (3290)	17
*seip-1(zu483)*	L4	herm	sp	0.07 (3416)	18
*seip-1(zu483)*	D1	herm	sp/ooc	45.0 +/- 6.9 (1800)	9
*seip-1(zu483)*	D2	herm	sp/ooc	57.6 +/- 6.3 (1800)	9
*seip-1(zu483) fog-2(q71)*	D1	female	ooc	27.7 +/- 6.4 (1049)	8
*seip-1(zu483) fog-2(q71)*	D2	female	ooc	6.2 +/- 5.1 (813)	7
*seip-1(zu483) fog-2(q71)*	D1	mated female	ooc	33.2 +/- 8.8 (1255)	9
*seip-1(zu483) fog-2(q71)*	D2	mated female	ooc	57.8 +/- 11.2 (1527)	11
*seip-1(zu483)* 15°C	D1	herm	sp/ooc	7.8 +/- 1.6 (1800)	9
*seip-1(zu483)* 15°C	D2	herm	sp/ooc	13.2 +/- 2.5 (1800)	9
*seip-1(zu483)* 25°C	D1	herm	sp/ooc	39.1 +/- 4.8 (1600)	8
*seip-1(zu483)* 25°C	D2	herm	sp/ooc	48.7 +/- 14.5 (1800)	9

* sp = sperm; ooc = oocytes

### nLD form in germ cells that contain few or no cLDs

As shown above, most nLDs in the adult intestine occur in the subanterior region, where cells have relatively few cLDs. Interestingly, we found that germ cells have very few, and often no cLDs (Fig 10C–10E). The hermaphrodite gonad has a very high level of fat, but that fat is concentrated in the gonad core until germ cells expand and take up core cytoplasm (Fig 10A). Thus, we wanted to determine whether nLD formation was induced by the near absence of cLDs around germ nuclei.

**Fig 12:**
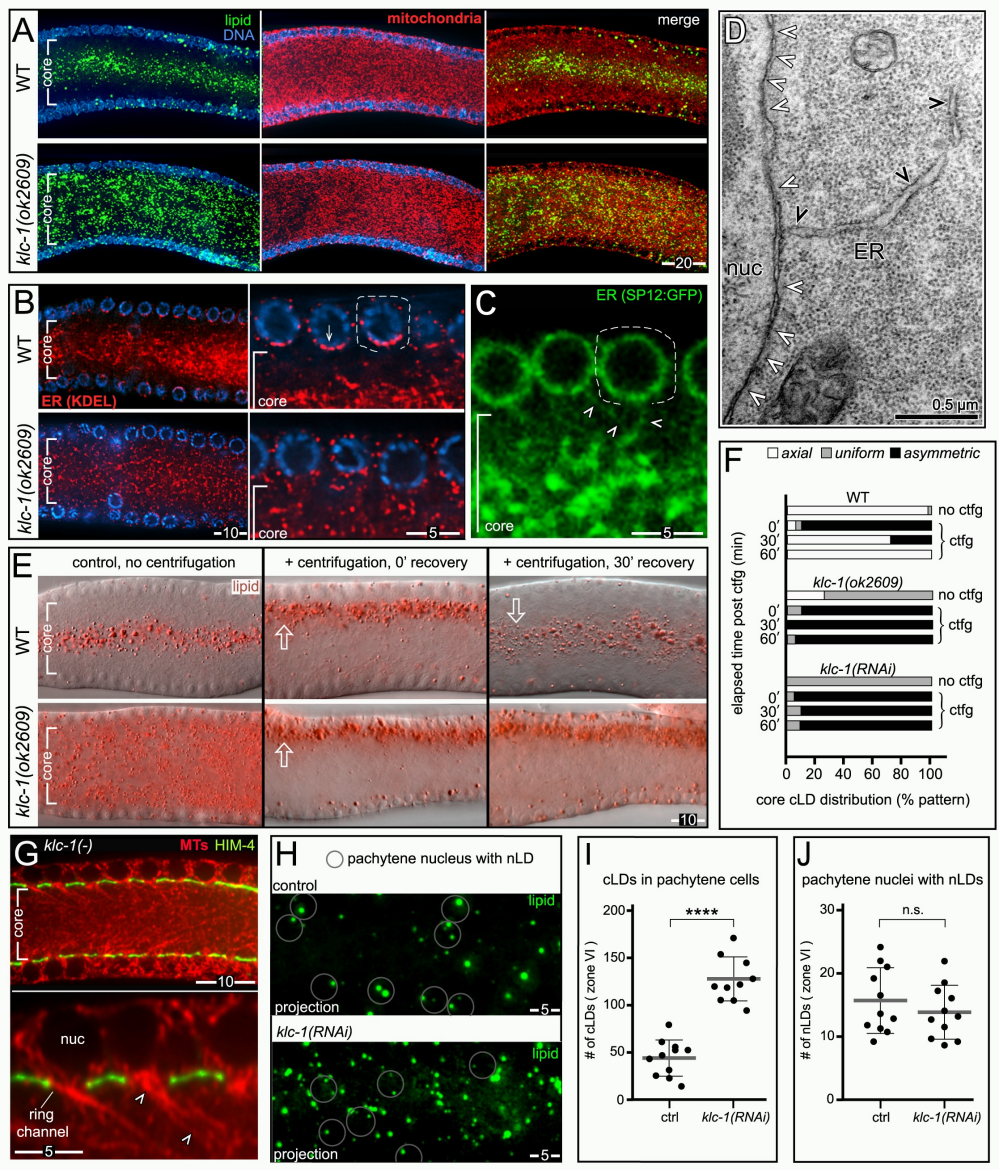
nLDs are not induced by cLD traffic away from germ cells. (A) Comparison of pachytene regions in D2 wild-type and *klc-1(ok2609)* mutant gonads stained for lipid (green, BODIPY), mitochondria (red, mitochondrial ATP synthase beta), and DNA (blue, DAPI). cLDs are concentrated along the axis of the core in the wild-type gonad, but not in *klc-1*/kinesin mutant gonads. A similar disruption of cLD distribution was observed in *klc-1(RNAi)* gonads and *unc-116*/kinesin heavy chain (RNAi) gonads (n = 22–35 gonads each), but not in RNAi experiments for the following motor proteins KLC-2 (kinesin light chain), KLP-2 (kinesin-like protein), KLP-16 (kinesin-like protein), DHC-1(dynein heavy chain), DHC-3 (dynein heavy chain), and CHE-3 (dynein heavy chain); 15–28 gonads scored for each. (B) Comparison of pachytene regions of wild-type and *klc-1(ok2609)* mutants gonads stained for the HDEL peptide (red), which is a C-terminal retrieval motif present on soluble ER-resident proteins. The high magnification insets show that most of the staining is concentrated near the base of a wild-type nucleus, facing the gonad core, but is not concentrated in *klc-1* mutant germ cells. (C) Live wild-type gonad expressing a transgenic marker for the ER (GFP fusion to the signal peptidase component SPCS-1/SP12 [112]). This generic marker for the ER shows the same concentration within the core as in Fig 12B, and further reveals fine strands of ER that run between the nuclear envelope and the core (arrowheads). (D) TEM of the nuclear envelope of a D2 germ cell. The image shows several nuclear pores (white arrowheads) and one of the infrequent connections between the envelope and the ER (black arrowheads). (E) Images of cLDs (red, oil red O) in the gonad core of centrifuged and control animals. Centrifugation floats the cLDs asymmetrically away from the axis of the core in both wild-type and *klc-1* mutant gonads, but cLDs only return to the axis in wild type. (F) Quantification of experiments shown in **Fig 12E**. (G) Image of pachytene nuclei in a *klc-1(ok2609)* mutant stained for tubulin (red, alpha-tubulin) and for the perimeter of the core (green, HIM-4/hemicentin). As for wild type, long microtubules (MTs, arrowheads) exit the pachytene cells through the ring channels and extend into the gonad core [56,63]. (H**)** Comparison of cLDs and nLDs in *klc-1(RNAi)* and control (empty RNAi vector) gonads; the gonads were stained for lipid (green, BODIPY) and for the nuclear envelope; optical z-stacks through the superficial nuclear layer were collected and analyzed. The images are maximum intensity z-projections of the uppermost nuclear level, with circles indicating the subset of nuclei with confirmed nLDs. *klc-1(RNAi)* increases the number of cLDs in the cytoplasm, but has little effect on the number of nuclei with nLDs. (I) Quantification of cLDs as in **Fig 10H**. Individual cLDs were counted manually from 3.9 μm optical z-stacks of the uppermost nuclear level. (J) Quantification of nLDs as in **Fig 10H**. Graphing was done in PRISM and the p-value calculated using the Welch’s t-test. **** P< 0.0001, n.s. not significant. Scale bars in microns as indicated.

We noticed that cLDs in the gonad core were further concentrated along the central axis of the core; the axial concentration of cLDs contrasts with mitochondria, which appear to be distributed uniformly in the core (Fig 12A). We next examined the distribution of ER by immunostaining gonads with an antibody that recognizes the C-terminal peptide HDEL: The motifs HDEL/KDEL occur in soluble proteins that function in the ER lumen (soluble ER-resident proteins), and are used to retrieve ER residents that become mislocalized into the Golgi during secretion [61]. We found that soluble ER residents in the core were concentrated toward the axis (Fig 12B), and ER residents within germ cells were concentrated near the core-facing surface of the nuclear envelope (inset, Fig 12B). A second, transgenic ER reporter for the signal peptidase SP12 showed a similar axial concentration in the core, and additional localization to fine strands of ER extending between the nucleus and the core (arrowheads, Fig 12C). By TEM, pachytene cells contained very little ER, with only a few connections between the ER and the envelope (Fig 12D). Indeed, pachytene germ cells had far less ER than any of multiple somatic cells examined by TEM, including gonad sheath cells which are adjacent to germ cells (S8 Fig). Thus, the near absence of cLDs in pachytene germ cells might result from the lack of ER, and/or trafficking of cLDs away from germ cells.

Lipid droplets have a low buoyant density that allows them to be separated readily from other organelles by centrifugation. We found that live worms that were centrifuged and then fixed and stained immediately showed a marked, asymmetrical shift of cLDs away from the core axis (Fig 12E). If the centrifuged worms were allowed to recover, most cLDs returned to the axis within about 30 minutes (Fig 12E, quantified in Fig 12F). This result suggests that cLDs traffic toward the core axis. Studies in other systems have shown that both cLDs and ER membranes can traffic on microtubules (MTs) [62], and *C*. *elegans* germ cells contain polarized MTs with plus-ends that extend through the ring channel and into the core [56,63]. Thus, we tested whether known or predicted microtubule motor proteins were required for the axial concentration of cLDs and ER. Mutations in, or RNAi depletion of, *unc-116* (kinesin heavy chain) and *klc-1* (kinesin light chain) both resulted in an abnormal, uniform distribution of both cLDs and ER (Fig 12A and 12B), and disrupted the normal, asymmetric localization of soluble ER residents within germ cells (inset, Fig 12B). *klc-1(ok2609)* null mutants appeared to have normal patterning of germ cell MTs (Fig 12G), suggesting that the cLD and ER phenotypes did not result from general cytoskeletal defects. We found that centrifugation shifted cLDs in *klc-1(ok2609)* mutant worms away from the axis, as in wild type, but that the cLDs remained shifted for a least one hour (Fig 12E, quantified in Fig 12F).

Together, these results suggest that the lack of cLDs around germ nuclei results at least in part from kinesin-mediated transport of cLDs, toward the core axis. We found that pachytene germ cells in *klc-1(RNAi)* animals had more than twice the normal number of cLDs (Fig 12H, quantified in Fig 12I). Nevertheless, the number of pachytene germ cells with nLDs did not change significantly (Fig 12H, quantified in Fig 12J). Thus, these results do not a support a hypothesis that nLD formation is driven by the local absence of cLDs.

### Identification and molecular analysis of nLD mutants

To determine whether, or how, nLDs impact the integrity of germ nuclei, we wanted to identify strains or mutants that increase the low frequency of nLDs. The N2 laboratory strain of *C*. *elegans* appears to have adapted to laboratory culture, and is distinct from all wild isolates [64]. Thus, we examined gonads in 11 diverse wild strains of *C*. *elegans*, and found that all had at least some germ cells with nLDs (S9 Fig). In most cases, the numbers and sizes of nLDs in the peak zone closely resembled the N2 strain; nLDs in the Hawaiian strain CB4856 were highly variable, but could be slightly larger and more numerous (S9 Fig). Forward genetic screens have been used previously to identify *C*. *elegans* mutants with "supersized" cLDs in the intestine, but those studies did not report nLD phenotypes. The published mutations alter peroxisomal fatty acid beta-oxidation (*maoc-1*, *dhs-28*, and *daf-22*) and the import of peroxisomal matrix enzymes (*prx-10*) [65,66]. We examined germ cell nLDs in each of these mutants, and in representative classes of additional Drop mutants (lipid droplet abnormal) whose molecular identities have not been reported (S9 Fig). The sizes and numbers of germ cell nLDs in these mutants appeared similar to wild type; some mutants had slightly fewer and/or smaller nLDs than wild type, and others had a few nLDs that were slightly larger than typical wild-type nLDs (S9 Fig). Studies in other systems have shown that seipin proteins can have important roles in nLD formation in some cell types, but not others [67]. We examined previously described *seip-1*/seipin null mutants [68,69], and found that most of the mutant gonads had no obvious nLDs, although rare nLDs could be slightly larger than wild type (S9 Fig). We conclude that nLDs are a common feature of *C*. *elegans* germ cells, but that several known mutations that affect cLDs have no major effects on germ cell nLDs.

**Fig 13:**
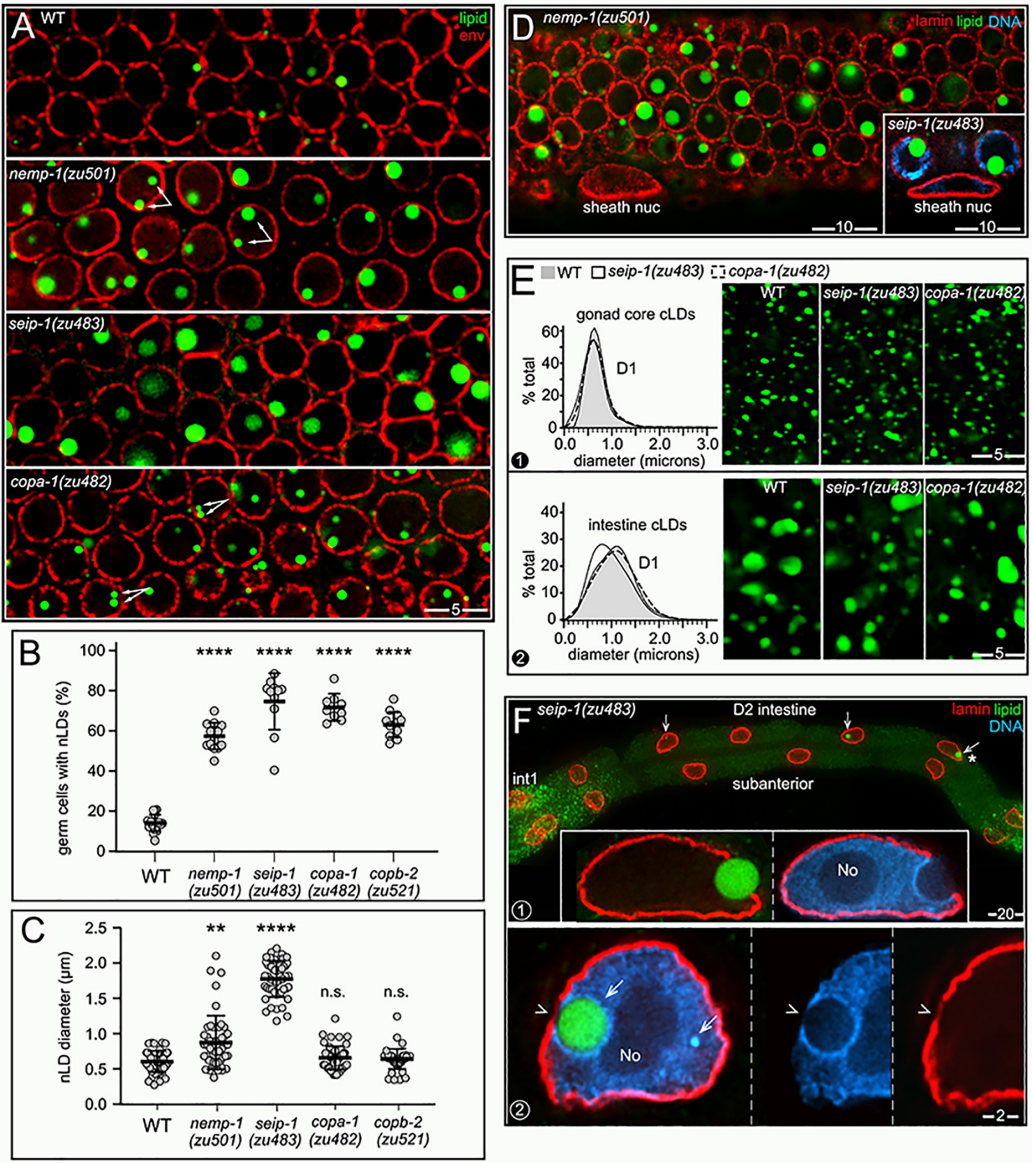
Mutants with abnormal nLD sizes or numbers. (A) Pachytene germ nuclei in wild-type and mutant gonads as indicated, showing lipid (green, BODIPY) and the nuclear envelope (red, NPP-9/RanBP2). The area shown corresponds to the peak nLD region in wild-type gonads (Fig 10H). Double arrows indicate multiple nLDs in single nuclei. Many of the mutant nuclei that appear to lack nLDs on the focal plane shown instead have nLDs visible on other focal planes. (B) Plot showing the percentage of germ cells with nLDs in the peak zone. (C) Plot comparing nLD diameters in the peak zone. (D) Images of the pachytene region of *nemp-1(zu510)* and *seip-1(zu483)* mutants, showing the absence of nLDs in gonad sheath nuclei. (E) Plot of cLDs sizes in the gonad core (panel 1) and intestine (panel 2; analyzed in the int2 cell group). The following numbers of cLDs were scored. (gonad core, intestine) = WT (4572, 5269); *seip-1(zu483)* (3316, 3379); *copa-1(zu482)* (2084, 3783). Curves were fitted using the Fit Spline/Lowess method to a plotted histogram with a bin size of 0.3 μm. (F) Panel 1 shows a D2 *seip-1(zu483)* intestine stained for lamin (red, LMN-1), lipid (green, BODIPY), and DNA (blue, DAPI). Note the relative lack of cLDs in the subanterior region, similar to D2 wild-type intestines. This is an atypical intestine where three nuclei have nLDs (arrows), including one ruptured nucleus (inset). Panel 2 shows a large nLD coated with heterochromatin but not lamin. Note that the nLD is adjacent to a lamin-deficient region of the envelope (arrowhead), as observed for a some wild-type nuclei with large nLDs. Graphing was done in PRISM; p-values were calculated using the Welch’s t-test. **** P< 0.0001, **P<0.01, n.s. not significant. Scale bars in microns as indicated.

**Fig 14:**
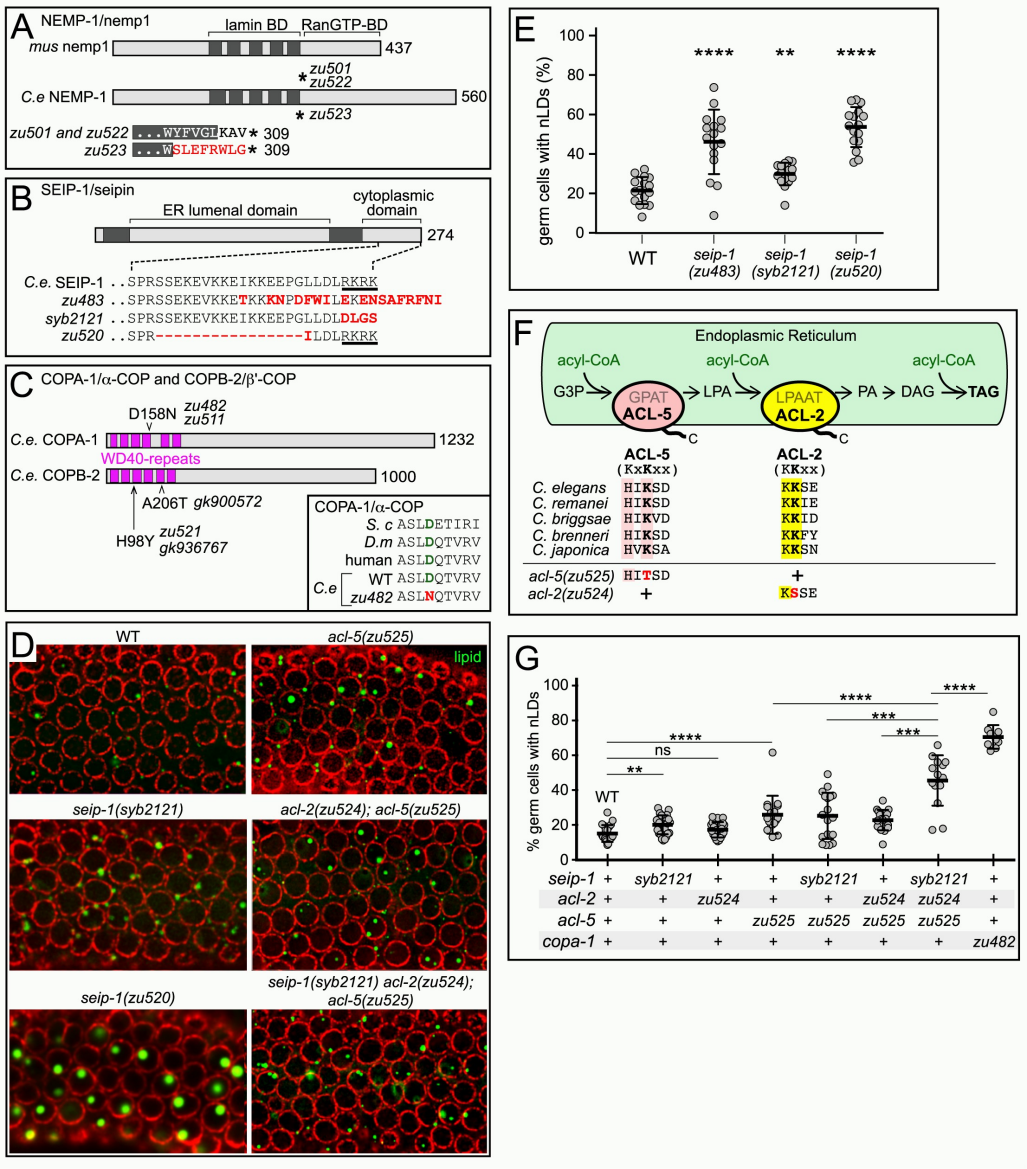
Gene products and analysis of nLD mutants. (A-C) Diagrams of wild-type and mutant protein products as listed. Dark grey boxes (NEMP-1, SEIP-1) indicate transmembrane domains, and protein sizes in amino acids are indicated to the right of each cartoon; amino acid replacements in the mutant proteins are shown in red, and stop codons are indicated by asterisks. The diagrams list both the original mutations (*zu501*, *zu483*, and *zu482*) identified in our genetic screens and mutations generated by gene editing. Both *nemp-1(zu501)* and *nemp-1(zu522)* result in identical truncated proteins, but the stop codon in *nemp-1(zu523)* results from a different frameshift that alters the intervening amino acids; BD = binding domain. *seip-1(zu483)* is a point mutation that causes a I260T substitution, followed by a 2 base pair deletion that shifts the reading frame of the C-terminus. *copa-1(zu482)* and *copa-1(zu511)* are identical mutations, but isolated in separate screens (see text). The mutations *copb-2(gk900572)* and *copb-2(gk936767)* are in strains identified by the *C*. *elegans* Million Mutation Project. (D) nLDs in the peak zones of wild-type or mutant gonads as labeled. The *seip-1(syb2121)* mutant lacking the presumptive retrieval peptide RKRK has slightly more nLDs than wild type, but the nLDs are fewer and smaller than in *seip-1(zu483)* mutants. Conversely, the *seip-1(zu520)* mutant, which retains the retrieval peptide but lacks adjacent residues, has both larger and more numerous nLDs than wild type. The *acl-5* mutant has more nLDs than wild type, and the triple *seip-1acl-2; acl-5* mutant has considerably more nLDs. (E) Plot of *seip-1* mutant germ cells with nLDs, as for **Fig 14D**. (F) Diagram showing a fat synthesis pathway involving the ER-resident membrane proteins ACL-5/GPAT and LPAAT/ACL-2. These proteins have conserved, predicted C-terminal retrieval motifs of the form KxKxx and KKxx, with the -3 lysine being the most critical residue. Gene editing was used to change the -3 residue, creating *acl-5(zu525)* and *acl-2(zu524)* mutants. G3P (glycerol-3-phosphate), GPAT (G3P acyltransferase), glycerol-3-phosphate acyltransferase) LPA (lysophosphatidic acid), LPAAT (LPA acyltransferase), PA (phosphatidic acid), DAG (diacylglycerol), TAG (triacylglycerol). (G) Plot of germ cells with nLDs, as for **Fig 14D**. Points in graphs represent individual gonads; plot shows mean and standard deviation. P value from unpaired t test with Welch’s correction**** *P*< 0.0001, ***P ≤ 0.01, **P ≤ 0.01, ns not significant.

We next used standard chemical mutagenesis to conduct a forward genetic screen for mutants with increased numbers or sizes of germ cell nLDs. Although several mutants appeared to have defects in both nLDs and cLDs, three appeared to have relatively specific nLD defects and were chosen for further analysis (Fig 13A). Molecular cloning showed that the mutated genes encode NEMP-1/NEMP1, SEIP-1/seipin, and COPA-1/α-COP (Fig 14A–14C). Each mutation results in a large increase in the number of germ cells with nLDs in the peak zone (Fig 13B); for example, nearly 80% of *seip-1(zu483)* germ cells in the peak zone have nLDs, compared to about 15% in wild type. In addition, each of the mutations greatly increases the numbers of nLDs outside the peak zone (see below). nLDs in *copa-1(zu482)* germ cells are about the same size as wild type, but nLDs in *nemp-1(zu501)* and *seip-1(zu483)* germ cells can be considerably larger (Fig 13C); the largest nLDs are about half the diameter of germ nuclei. Similar to wild type, none of the mutants had nLDs in gonad sheath cells (0/52 total; Fig 13D) or pharyngeal/valve cells (0/28 total), which are adjacent to germ cells and intestinal cells, respectively.

Each mutant appeared to have normal sizes of cLDs in the gonad core and in the intestine (Fig 13E). Surprisingly, none of the mutants appeared to increase the percentage of intestinal nuclei with nLDs, and *copa-1(zu482)* mutants appeared to have far fewer intestinal nLDs than wild type (Table 1). As in wild type, intestinal nLDs in the mutants were coated with heterochromatin, either with or without an additional coating of lamin (Fig 13F). Giant nLDs in *nemp-1(zu501)* and *seip-1(zu483)* could be found adjacent to lamin-deficient regions of the envelope, as in wild type (panel 2, Fig 13F), and nuclear ruptures were observed in *seip-1(zu483)* intestines (Table 1 and inset in panel 1, Fig 13F). Thus, these mutations appear to affect nLDs, rather than cLDs, and to predominantly affect nLDs in germ cells.

### nLD mutants are defective in proteins localized to the INM or involved in lipid synthesis

NEMP-1 is the sole *C*. *elegans* ortholog of vertebrate Nemp1 (also called TMEM194A), and is a conserved INM protein with five transmembrane domains (Fig 14A) [70–72]. The function of NEMP-1/Nemp1 is not known; it has been suggested to have roles in *Xenopus* neural development, tamoxifen resistance in human breast cancer cells, and to contribute to the stiffness of the nuclear envelope [70,72,73]. Nemp1 localizes with lamin, and appears to interact with lamin through a region spanning the five transmembrane domains (Fig 14A) [74]. The C-terminal half of Nemp1 extends into the nucleoplasm, and includes a domain that can bind RanGTP [70]. The semi-dominant *nemp-1(zu501)* mutation is predicted to encode a truncated membrane protein that lacks the RanGTP domain (Fig 14A). To confirm that the *nemp-1(zu501)* mutation was responsible for the nLD phenotype, we used Crispr-Cas9 to engineer identical (*zu522*) and similar (*zu523*) mutations, and found that both mutations resulted in apparently identical nLD phenotypes.

SEIP-1 is the sole *C*. *elegans* ortholog of vertebrate seipin, an ER-resident membrane protein that functions in lipid droplet formation (see Introduction). Seipin has a highly conserved, central domain located in the ER lumen, and a divergent C-terminal tail that extends into the cytosol (Fig 14B). Both dominant and recessive mutations in the luminal domain and the C-terminal tail are responsible for severe human diseases [75]. *seip-1(zu483)* is a semi-dominant, compound mutation affecting the cytoplasmic tail; it causes an I260T substitution, and includes an additional frameshift mutation that changes several amino acids at the C-terminus (Fig 14B).

COPA-1 is the sole *C*. *elegans* ortholog of α-COP, a COPI subunit. Recent genetic and biochemical studies have demonstrated a role for the COPI complex in regulating lipid droplets (see Introduction). However, the best known function of COPI vesicles is in the retrograde transport of ER-resident proteins from the Golgi to the ER (reviewed in [76]). ER-resident membrane proteins that are missorted into the secretory pathway, or that are temporarily shuttled to the Golgi for posttranslational modifications, can be returned to the ER through retrieval motifs recognized by α-COP and a related COPI subunit called β’-COP. The best characterized retrieval motifs for ER-resident membrane proteins are C-terminal dibasic peptides that are both necessary and sufficient for retrieval; the peptide motifs include KKxx, RKxx, and KxKxx, with the -3 lysine being the most critical residue. Structural studies have shown that an N-terminal WD40-repeat region in both α-COP and β’-COP forms a beta-propeller domain with a central binding pocket for the retrieval peptide [77,78]. The semi-dominant *copa-1(zu482)* mutation substitutes asparagine for a highly conserved, acidic residue in the binding pocket for the dibasic retrieval peptide (Fig 14C). Although retrieval sequences have not been analyzed in *C*. *elegans*, we found that several known and predicted ER-resident membrane proteins in *C*. *elegans* have conserved C-terminal sequences that match the KKxx, KxKxx or variant motifs (S10 Fig).

### The nLD phenotype of COPI mutants results from a retrieval defect

We wanted to determine whether the *copa-1* mutation was affecting nLDs by a retrieval defect or through the other roles ascribed for COPI components in lipid droplets (see Introduction). Our genetic screens identified three additional, independent mutants with excess nLD phenotypes similar to *copa-1(zu482)*. For example, the sizes and temperature dependence of nLDs in these mutants were similar to *copa-1(zu482)*, but different from the *nemp-1* and *seip-1* mutants (S7 Fig). Because the COPI subunits α-COP and β’-COP have similar roles in binding retrieval peptides, we sequenced both *copa-1*/α-COP and *copb-2/*β’-COP in each mutant. None of the additional mutants had a mutation in *copb-2*, but one had the identical mutation as *copa-1(zu482)* (Fig 13C). We next asked whether *copb-2* mutants with nLD phenotypes might have been recovered in the *C*. *elegans* "million mutation" project [79]. This study generated 2000 heavily mutagenized but viable strains that were grown clonally, sequenced, and then archived as a library of frozen stocks. Database searches showed that the library contained multiple strains with missense mutations in the WD40 repeat domains of either *copa-1* or *copb-2* [80]. None of strains with *copa-1* mutations had nLD phenotypes, but two of 7 strains with *copb-2* mutations (*gk900572* and *gk936767*) had nLD phenotypes similar to *copa-1(zu482)*. Both the *copb-2(gk900572)* and *copb-2(gk936767)* mutations altered amino acids close to critical residues in the predicted binding pocket for the retrieval motif (S10 Fig). Because the library strains have an average of about 400 mutations, we outcrossed each strain eight times to wild type by scoring for the nLD phenotype. DNA sequencing showed that both of the outcrossed lines retained their respective *copb-2* mutations, indicating close linkage with the nLD phenotype. Finally, we used Crispr-Cas9 editing to generate a *copb-2(zu521)* mutation identical to *copb-2(gk936767)*, and found that it resulted in a similar, semi-dominant nLD phenotype (Fig 13B and 13C).

### Multiple ER-residents involved in fat regulation likely contribute to the nLD phenotype of COPI mutants

The above results support a hypothesis that the nLD phenotypes of COPI mutants result from the failure to retrieve one or more ER-resident membrane protein(s). Interestingly, SEIP-1 is an ER-resident membrane protein, and the cytoplasmic domain of SEIP-1 shows little conservation in *Caenorhabditis* other than the C-terminal RKRK peptide (S10 Fig). RKRK is a functional retrieval peptide in other systems [81] and is absent in the *seip-1(zu483)* mutant (Fig 14B). We found that *copa-1(zu482)*; *seip-1(tm4221 null)* double mutants showed a marked reduction, or complete absence, of germ cell nLDs, suggesting that SEIP-1 function contributes to the excess nLD phenotype of *copa-1(zu482)* single mutants (S9 Fig). We next used Crispr-Cas9 gene editing to change the presumptive retrieval peptide in SEIP-1 from RKRK to DLGS (Fig 14B). Although the resulting *seip-1(syb2121)* mutant had an excess nLD phenotype in germ cells, the phenotype was variable and minor compared with that of *seip-1(zu483)* or either COPI mutant (Fig 14D and 14E). We next used Crispr-Cas9 to engineer a complementary *seip-1(zu520)* mutation that retained the C-terminal RKRK peptide, but deleted several adjacent residues (Fig 14B). The *seip-1(zu520)* mutant had a strong, excess nLD phenotype resembling that of *seip-1(zu483)* (Fig 14D and 14E). Moreover, the nLDs in *seip-1(zu520)* were abnormally large (Fig 14D), with sizes similar to nLDs in *seip-1(zu483)* mutants, but much larger than nLDs in wild-type, *seip-1(syb2121)*, *copa-1(zu482)*, or *copb-2(zu521)* mutants. These results show that defects in the presumptive retrieval peptide of SEIP-1 contributes to the excess nLD phenotype of *seip-1(zu483)* mutants, but is not the sole or principal cause of the phenotype.

To identify different, or additional, ER-resident membrane proteins that might contribute to the excess nLD phenotype of the COPI mutants, we searched a *C*. *elegans* database of predicted membrane proteins to find ER-resident proteins that (1) contained a C-terminal dibasic motif of the form KKxx or KxKxx, and (2) functioned in fat (triacylglyercol) synthesis [82]. This search identified ACL-2 and ACL-5 as potential candidates (Fig 14F). The major pathway that converts glycerol-3-phosphate to triacylglycerol involves the sequential addition of acyl groups from acyl-CoA to the glycerol backbone. The first acyl group is added by GPAT acyltransferases that are rate limiting for fat synthesis, and the second group is added by LPAAT acyltransferases. *C*. *elegans* has two non-mitochondrial GPATs, and two LPAATs [83]. However, only ACL-5/GPAT and ACL-2/LPAAT have candidate C-terminal retrieval peptides (Fig 14F). Both motifs are conserved in ACL-5 and ACL-2 proteins in *Caenorhabditis* species, suggesting that they have functional significance (Fig 14F). Thus, we used Crispr-Cas9 editing to change the critical -3 lysine in the dibasic motifs to either threonine [*acl-5(zu525)*] or to serine [*acl-2(zu524)*] (Fig 14F). The *acl-5(zu525)*, but not *acl-2(zu524)*, mutation caused a clear nLD phenotype in germ cells (Fig 14D, quantified in Fig 14G). Moreover, triple mutant *seip-1(syb2121) acl-2(zu524); acl-5(zu525)* animals had a much stronger nLD phenotype than the single or double mutants. These results support a hypothesis that germ cell nLDs can result from a defect in the COPI-mediated retrieval of ER-resident membrane proteins involved in fat synthesis. However, an important but unresolved issue is how COPI vesicles and retrieval peptides, which are thought to function in retrograde transport from the Golgi to the ER, prevent the accumulation of ER-residents in germ nuclei (see Discussion).

### *seip-1* mutant nLDs resemble wild type nLDs except for size and number

We chose *seip-1(zu483)* mutants for a detailed characterization of nLDs and their effects on development because the *nemp-1* and *copa-1* mutants had additional phenotypes that were unlikely to be related directly to nLDs (see analysis in S11 Fig). For example, *copa-1(zu482)* males appeared to have a moderate mating or infertility defect, although nLDs are rare in males (S11 Fig). We found that nLDs in *seip-1* mutant germ cells closely resembled those in wild-type germ cells, except for their size. The nLDs lacked any evidence of a heterochromatin coat, but many had "bristle coats" similar to some wild-type nLDs (Fig 11C). Germ nuclei with nLDs typically had only one nLD in *seip-1(zu483)* mutants (97%, n = 681), as observed for wild type but in contrast to *copa-1(zu482)* mutants (Fig 13A). Single nLDs could suggest that formation is triggered by a unique event in meiotic progression, or that an nLD occupies an exclusive site on the nuclear envelope. In our TEM analysis we looked for, but did not observe, unique features of the envelope near the early nLDs, including the position of the centrosome or a connection with the ER. Moreover, *seip-1* mutant germ nuclei lacked the nuclear vesicles, membrane tubules, nMFBs, and cysts observed in wild-type intestinal nuclei.

**Fig 15:**
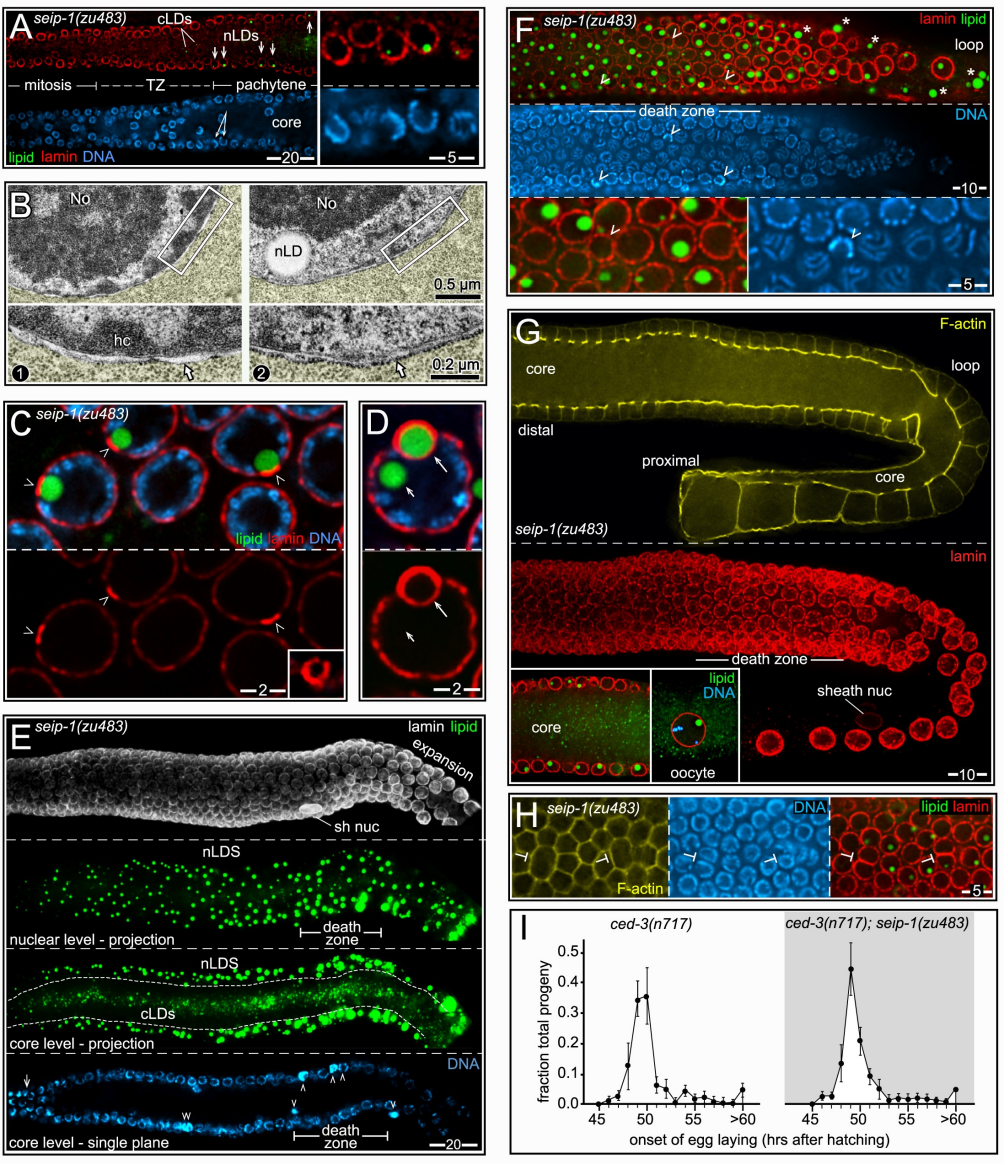
Characterization of nLDs in *seip-1(zu483)* gonads. (A) Mitotic to early pachytene region of a *seip-1(zu483)* gonad, showing the first appearance of nLDs (arrows and inset) in cells passing through the transition zone (TZ, see **Fig 10A**). (B) TEM of *seip-1(zu483)* germ cells. The left panel shows an interphase nucleus from the mitotic zone, which lacks nLDs. The right panel shows an early pachytene-stage nucleus with an nLD. The early pachytene nucleus has relatively little peripheral heterochromatin (hc), and far more ribosomes on the ONM (arrows). (C) Early pachytene region of *seip-1(zu483)* gonad showing lamin concentrated in patches at the base of each nLD. The inset is an orthogonal view of a single lamin patch, showing the asymmetric, horseshoe shape. (D) *seip-1(zu483)* germ cell showing a rare example of an nLD that appears to be coated with lamin (long arrow). The germ cell is also atypical in containing a second nLD (short arrow). (E) Image of the nLD-containing area of a *seip-1(zu483)* D2 gonad, from early pachytene to the loop region. The gonad is stained for lamin (white, LMN-1/lamin), lipid (green, BODIPY), and DNA (blue, DAPI). The projections are of 6 μm z-stacks. Essentially all lipid droplets visible in the nuclear level are nLDs, while the core projection shows cLDs in the core and nLDs in the flanking, peripheral nuclei. Note that the core cLDs are much smaller than the nLDs. The bottom panel shows nuclei (DAPI, blue) in a single focal plane through the middle of the gonad; the arrow at far left indicates transition zone nuclei as in **Fig 15A**. Arrowheads point to apoptotic nuclei with compacted DNA, and the double arrowhead indicates a binucleate apoptotic cell (see also **Fig 11D**). Note that large numbers of germ cells retain nLDs at they pass through the major death zone. (F) Comparison of nuclear sizes in the mid-pachytene to loop region of a D1 *seip-1(zu483)* gonad; staining shown for lamin (red, LMN-1), lipid (green, BODIPY), and DNA (blue, DAPI). The image is a single optical plane through the top nuclear level. Most nuclei increase in size uniformly from distal to proximal, but three nuclei (arrowheads) are noticeably smaller than their neighbors; the DNA panel shows that these nuclei are apoptotic with compacted chromatin. The large, lipid-containing objects that are not nuclear (asterisks) are within phagocytic sheath cells that engulf apoptotic germ cells (see **S11 Fig** for additional details). (G) Comparison of germ cell sizes in a *seip-1(zu483)* gonad; cell boundaries are outlined by F-actin (yellow, phalloidin), and nuclei are stained for lamin (red, LMN-1). Note the uniform increase in cell sizes as cells move distal to proximal; the insets show nLDs from two regions, including an nLD in an oocyte nucleus. (H) Binucleate cells in the early pachytene region of a *seip-1(zu483)* gonad; cell boundaries are indicated by staining for F-actin (yellow, phalloidin). The gonad contains low numbers of binucleate germ cells (T-bars), similar to wild type. Binucleate cells first appear in about the same region as nLDs, and undergo apoptosis as they move further proximal. When present, nLDs were usually found in both nuclei (23/28 binucleate cells), but could instead be in only one nucleus (5/28 binucleate cells). (I) Plot comparing the rate of larval development in apoptosis-defective *ced-3(n717)* mutants (n = 1581 larvae) with *ced-3n717);seip-1(zu483)* double mutants (n = 1445 larvae). Synchronous newly hatched larvae were allowed to develop and scored when they produced their first eggs. Most animals in both populations reached adulthood and began producing eggs at t = 49–50 hrs, but a small fraction of animals had not produced eggs by t = 60 hrs.

nLDs in *seip-1* mutant gonads were not detected in the mitotic zone (n = 63 gonads) but were present in early pachytene cells (Fig 15A). This localization agrees with the earliest, albeit rare, examples of nLDs in wild type (Fig 10H). In comparing cells in the two regions by TEM, we noticed that interphase nuclei in the mitotic zone had abundant peripheral heterochromatin, similar to intestinal nuclei, but that early pachytene nuclei had very little peripheral heterochromatin (Fig 15B). In addition, early pachytene nuclei showed a marked increase in the numbers of ribosomes on the ONM (Fig 15B), suggesting that nLD formation might be linked to an increase in ONM-associated protein synthesis. Lamin appeared to be concentrated at the base of each of the nLDs in early pachytene nuclei (arrowheads in Fig 15C), and persisted through much of the pachytene stage. A similar concentration of lamin was not visible in any germ cells in the mitotic zone or in intestinal cells, and only very rare germ cell nLDs were completely surrounded by lamin (Fig 15D). Most of the nLDs in *seip-1(zu483)* germ cells remained adjacent to the envelope throughout pachytene, and the largest envelope-associated nLDs were associated with prominent nuclear bulges (arrows, Fig 11E). A subset of nLDs appeared to move away from the envelope and into the nucleolus, and those nuclei were spherical (panels 5, 6 in Fig 11E).

nLDs in *seip-1* mutant germ cells increased progressively in size during pachytene, similar to wild type (Fig 15E and 15F). The size of a nLD relative to the nuclear diameter in *seip-1(zu483)* germ cells could be as large or larger than in wild-type intestinal cells, but we did not find any examples of ruptured germ nuclei (n>2000 germ cells). Similar to wild type, the percentage of *seip-1* mutant nuclei with nLDs appeared to decline after the death zone (Fig 15E and 15F). However, some nLDs in *seip-1* mutant gonads persisted in enlarged oogonia, and a few mature, cellularized oocytes had nLDs (inset, Fig 15G).

### *seip-1* mutant gonads appear normal and produce healthy progeny

*seip-1(zu483)* gonads closely resembled wild type in general morphology, with most germ nuclei and germ cells increasing in size uniformly during meiotic progression (Fig 15F and 15G); the few, atypically small germ nuclei/cells were apoptotic (arrowheads, Fig 15F). *seip-1* mutants appeared to have approximately normal numbers of both mononucleate and binucleate apoptotic cells (Fig 11D), with a frequency of nLDs in apoptotic cells that was comparable to surrounding, non-apoptotic cells (Fig 15H). To address whether the presence of nLDs was deleterious for progeny, we compared egg viability in apoptosis-defective *ced-3(n717)* mutant animals with *ced-3(n717); seip-1(zu483)* double mutants. Nearly all of the eggs from both mutant strains hatched (99.0 +/- 0.8%, n = 2541 eggs versus 98.9 +/- 0.7%, n = 1930 eggs, respectively). To compare the rate of development of the resulting progeny, we synchronized newly hatched larvae from both mutants, then measured the elapsed time before the resulting adults produced their first eggs. Both strains showed very similar developmental profiles, with most progeny growing to egg-producing adults by 48–51 hours (Fig 15I). Together, these results suggest that a large fraction of the volume of a meiotic nucleus can be filled with lipid without triggering apoptosis, and that those germ cells produce viable gametes and healthy progeny. By contrast, nLDs in somatic intestinal cells appear to be associated with multiple forms of nuclear damage.

## Discussion

We found that nLDs occur in *C*. *elegans* intestinal cells and germ cells. All of the wild strains of *C*. *elegans* examined in the present study had nLDs; these strains were isolated from diverse geographical locations, suggesting that nLDs are a widespread feature of *C*. *elegans* biology. Our analysis focused on animals grown under standard laboratory conditions at 20°C and fed a diet of *E*. *coli* strain OP50. We found that the number of germ cell nLDs is markedly reduced in animals cultured at lower temperature, and wild *C*. *elegans* strains likely have a range of preferred growth temperatures. *E*. *coli* has been the standard diet for decades of research on *C*. *elegans*, but diets of different bacteria, and even different strains of *E*. *coli*, are known to alter fat storage and metabolism [84]. Moreover, recent studies have shown that a diet of OP50 provides only a low level of vitamin B12 relative to other bacterial diets [85]. Although nLDs can occur in animals and humans, studies on cultured cells have generally used media supplemented with fatty acids. Here, we examined nLDs in unmated, well-fed animals that naturally undergo changes in intestinal fat during egg production. Much larger changes in intestinal fat occur during starvation, or when the normal egg-laying period is extended by mating. Imminent starvation is an integral feature of the natural biology of *C*. *elegans*, driven by the organism’s short generation time, self-fertilization, and large brood size, and males arise spontaneously in normal hermaphrodite populations from X chromosome nondisjunction [86].

Our results support a hypothesis that nLD formation is linked to general fat synthesis, rather than the level of stored fat or specific developmental events. nLDs were found in tissues synthesizing fat, either in preparation for export to the gonad (L4 hermaphrodite intestine), export to eggs (adult hermaphrodite gonad), or to replace depleted fat (subanterior region of adult intestine). The frequency of nLDs in adult hermaphrodite gonads correlated with changes in the rate of oogenesis, either by shifting the culture temperature (S7 Fig), or by mating *fog-2* mutants (Table 1 and S7 Fig). nLDs occurred infrequently in male intestines that have high, apparently stable, levels of stored fat (Fig 9B), and nLDs were not found in the high-fat sheath cells of *seip-1(zu483)* mutants (Fig 13D); the mutant sheath cells likely accumulate lipid from phagocytosis of apoptotic germ cells with nLDs, rather than synthesizing fat *de novo* (Figs 15F and S11). Although nLDs are found in all hermaphrodite gonads and are rare or absent in male gonads, both hermaphrodite and male germ cells contain few or no cLDs (Figs 9A and 10D); the hermaphrodite cLDs appear to be trafficked away from germ cells and toward the core (Fig 12).

If nLDs have a beneficial role in development, one possibility is for the rapid expansion of nuclei in late oogenesis. In systems such as yeast, nuclear size increases gradually with cell growth [87]. By contrast, oogonia nuclei in *C*. *elegans* expand very rapidly as cells fill with pre-made core cytoplasm (Fig 10A; [56]). nLDs reach their maximum size just before the expansion zone, but decrease in size during nuclear expansion. Because there is no obvious decrease in the sizes of cLDs at this stage, we speculate that nuclear-specific lipases might be activated during nuclear expansion. *seip-1(zu483)* germ nuclei that lack nLDs can be adjacent to nuclei with giant nLDs (Fig 15F), but we did not detect discontinuities in the sizes of expanding, non-apoptotic nuclei (Fig 15F and 15G). This result argues that nuclear expansion does not require nLD storage of lipid, at least under standard feeding conditions, but leaves open the possibility that nLDs might have a role in nutrient-poor environments.

### nLDs and nuclear damage in intestinal cells

By contrast with the gonad, the adult intestine lacks an apoptosis pathway and stem-cell niche to remove and replace damaged cells. Thus, nuclear damage must be repaired to maintain intestinal function. Our results show that some intestinal nuclei are damaged in adults during the self-fertile reproductive period, and suggest that at least some of this damage is repaired. Our strongest case for a direct role of nLDs in driving nuclear damage is the nLD-associated rupture of intestinal nuclei in L4 and D1 hermaphrodites. Recent studies in multiple systems have shown that nuclear ruptures occur both in vitro and in vivo, and can be repaired (reviewed in [88]). Although most nuclear ruptures are healed within minutes, others can take hours to seal; remarkably, even the latter examples do not necessarily trigger cell death. We found that some giant nLDs in intestinal cell contact what appear to be lamin-deficient regions of the nuclear lamina, suggesting that these sites might be prone to rupture (panel 2, Fig 13F and S2 Fig). Nuclear ruptures are associated with multiple human laminopathies, and can occur in normal cells that experience mechanical stress, as from migration in confined environments [89,90]. *C*. *elegans* moves by sinusoidal undulations that cause significant tissue deformation [91]. Thus, rupture might result from locomotion-dependent stresses on nuclei that are already compromised by giant nLDs. This hypothesis could be tested in future studies on *C*. *elegans* mutants that are viable, but nearly paralyzed.

**Fig 16:**
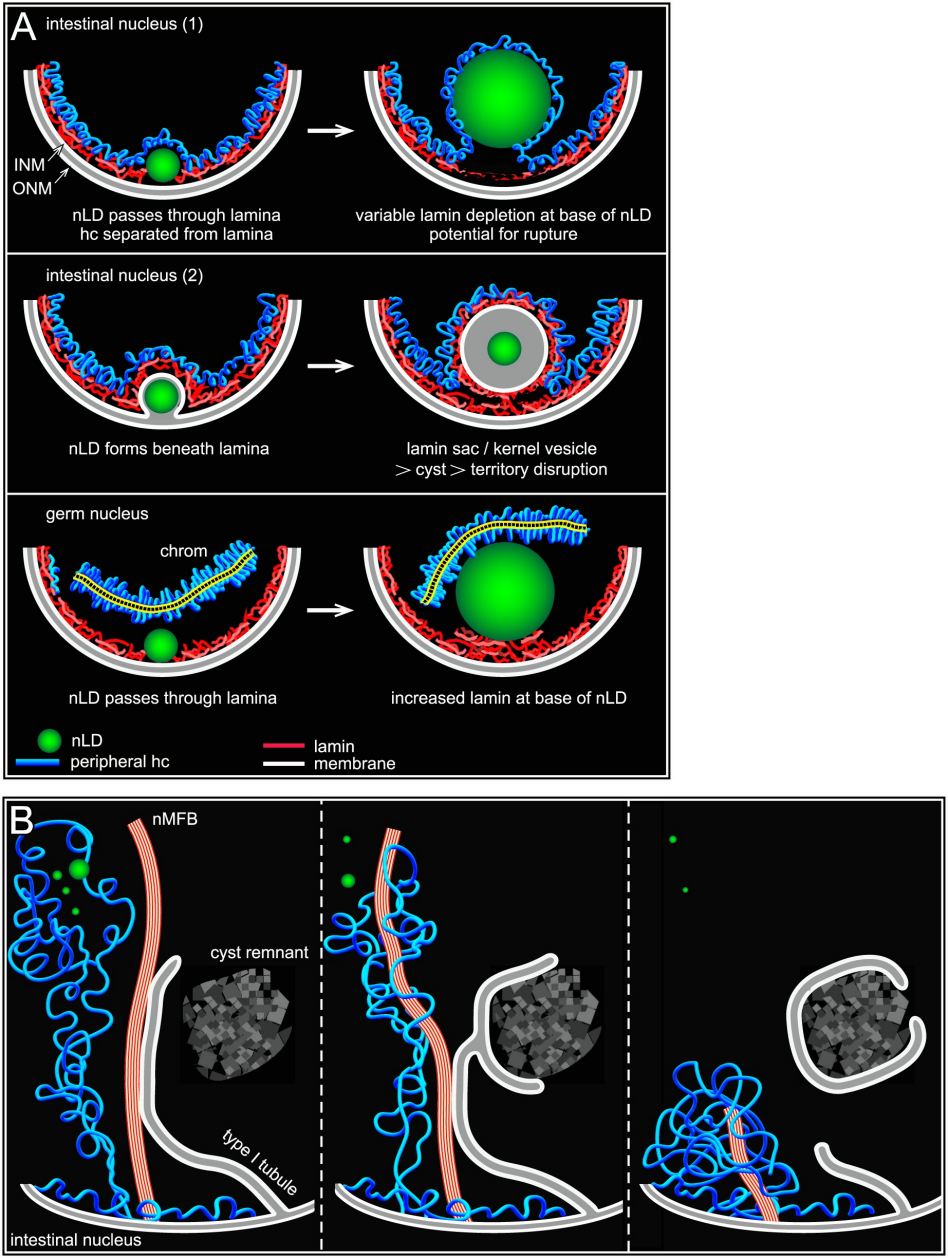
nLD-associated nuclear damage and repair. (A) Summary cartoon comparing damage-associated nLDs in intestinal nuclei with nLDs in germ nuclei, which do not appear to be deleterious. (B) Speculative model for repair of nLD-associated damage in intestinal nuclei. Lipolysis of a heterochromatin-coated nLD leaves inappropriate heterochromatin in the nuclear interior; nMFBs can bind to and return the heterochromatin to the periphery. nLDs contained in lamin sacs/kernel vesicles can form cysts and lyse, leaving debris and damaged nucleoplasm. Tubules in the type I nucleoplasmic reticulum grow into the nuclear interior, possibly by association with nMFBs, and engulf damaged materials for removal.

A second form of nLD-driven damage in intestinal nuclei might result from peripheral heterochromatin that is stripped from the lamina and displaced into the nuclear interior. The loss of heterochromatin and attendant derepression of silenced genes has long been proposed as a contributing factor in aging, and populations of aging *C*. *elegans*, *Drosophila* and humans have all been shown to lose peripheral heterochromatin (reviewed in [92]). Our results strongly suggest that heterochromatin is lost from the periphery of hermaphrodite intestinal nuclei during the self-fertile period (Fig 6E), but we did not address whether silenced genes were derepressed by these events. For example, lamin sacs that form by the envelope have prominent coatings of heterochromatin in D1 hermaphrodites, but not in D3 hermaphrodites (Figs 6E and 9E). Several observations support a hypothesis that nLD formation drives a loss of peripheral heterochromatin and, importantly, might separate chromatin from potential regulatory proteins associated with the lamina. First, nearly all of the nLDs with a prominent coat of heterochromatin are near regions of the envelope that appear to be relatively deficient in peripheral heterochromatin (Fig 2C). Second, the smallest, presumably nascent, nLDs that are adjacent to the INM appear to have an asymmetric, partial coating of heterochromatin, consistent with an inpocketing of peripheral heterochromatin (Fig 2H). Although some nLDs are also surrounded by lamin, and might recruit heterochromatin secondarily through interactions with lamin, many nLDs have no detectable lamin coating (Figs 3A and 6A, panel 1). Third, the giant nLDs at nuclear ruptures invariably had a hemispherical coat of heterochromatin, rather than a complete coat (Fig 2D). A hemispherical coating might not be expected if the coat was a random, aggregate of nucleoplasmic heterochromatin, but could occur if the coat originated from a site-specific inpocketing of peripheral heterochromatin (Fig 16A). Finally, most of the small nLDs in the interior of D2 nuclei lacked heterochromatin coats (Fig 2J); many of these nLDs likely originate from INM-derived type I tubules that penetrate the nuclear lamina, rather than forming at the INM beneath the lamina (Fig 5C).

A third possible mode of nLD-driven nuclear damage is the disruption of three-dimensional chromosome territories by nucleoplasmic cysts. DNA FISH experiments have shown that chromosomes in *C*. *elegans* intestinal nuclei occupy non-overlapping territories, as found in other animals [93]. Although chromosomal territories are evident in D1 intestines, they are disrupted or disappear by the D10 stage; the events driving territory disintegration are not known. We found that D3 and D4 nuclei can contain multiple, large cysts, and we imagine that cyst formation/removal might profoundly alter the relative positions of neighboring chromatin. The cysts appear to arise as herniations of lamin sacs/kernel vesicles (Fig 7E and 7H), and the cysts can contain small nLDs that likely originate from the sac (Fig 7C and 7H). Thus, we propose that nLDs forming at the INM have two main developmental trajectories that lead to nuclear damage (Fig 16A). If a developing nLD penetrates through the lamina, it separates the peripheral heterochromatin from the lamina and potential lamina-associated regulatory proteins; continued growth of these nLDs creates an additional threat of nuclear rupture. If instead the nLD doesn’t penetrate the lamina, it remains confined in a lamin sac which has the potential to degrade with age and form a cyst.

Although many lamin sacs contain lipid in D1 nuclei, most sacs in older nuclei do not. The lipid contents could be too small to detect by light microscopy, extracted during sample preparation, or be lost in vivo as sacs age and deteriorate; indeed, our images of kernel vesicles appear to show an age-dependent deterioration. However, it also is possible that sacs forming in later nuclei never contained lipid, and instead were triggered by other events such as membrane damage. A previous *C*. *elegans* study showed that multiple somatic nuclei, including intestinal nuclei, accumulated "dots" of intranuclear lamin during aging [94]. Moreover, the images of dots presented in that study for adults at D14 (present timescale, Fig 1A) resemble the lamin sacs reported here for young adults. Thus, it would be interesting to determine whether nLDs continue to form in nuclei after the D4 stage.

### Damage repair and debris removal in intestinal nuclei

Our results suggest that intestinal nuclei might have systems to dispose of aberrant nucleoplasm and degraded vesicles associated with nLDs, and to restore displaced heterochromatin to the periphery (Fig 16B). We found that a few D2 nuclei, and several D3 and D4 nuclei, contain large patches of aberrant nucleoplasm that appear to be remnants of lysed cysts (Fig 8A). This material and degraded kernel vesicles both appear to be engulfed by membranes (Fig 8B), then clustered and possibly removed (Figs 8C–8F and S4). Targeted removal and degradation of nuclear material, or nucleophagy, has been described in many animals cells, and is associated with various stress conditions, disease states, and aging (reviewed in [95]). Mechanisms of nucleophagy are poorly understood, but have been best studied in yeast where a cytoplasmic protrusion from the ONM is surrounded and removed by a lytic vacuole for degradation. Similarly, we found protrusions of the ONM that appeared to contain membranous debris in our TEM analysis of D4 nuclei (S4 Fig).

We propose that nuclear bundles of microfilaments, or nMFBs, play a role in repairing damaged intestinal nuclei. The nMFBs contact nLDs and other vesicles, and occur frequently within vesicle clusters. Some, but not all, nMFBs appear to become heavily coated with condensed chromatin (Fig 3B and 3F), particularly in older hermaphrodites (Fig 8E). Despite the controversial history of nuclear actin, recent studies have demonstrated convincingly that both G-actin and F-actin occur in many nuclei, and have suggested roles for nuclear actin that include gene repositioning, transcription, and DNA repair [96–99]. Here, we propose that nMFBs function essentially as trolling lines within the large intestinal nuclei, scavenging inappropriate materials from the nuclear interior and moving them to the periphery for removal (Fig 16B). Because actin can bind lamin, and lamin can interact with heterochromatin [100,101], nMFBs likely have the potential to bind heterochromatin, or heterochromatin-coated nLDs and lamin sacs. Thus, nMFB motility or collapse might return/cluster any bound cargo to the nuclear periphery (Fig 16B).

The nMFBs might also have an indirect role in nuclear repair by directing the growth of tubules in the type I nucleoplasmic reticulum; such tubules could be the source of the membranes that enclose aberrant nucleoplasm and vesicles in the nuclear interior (Figs 8B and 16B). Tubules in a type II nucleoplasmic reticulum are invaginations of both nuclear membranes, and enclose a lumen that is continuous with the cytoplasm; thus, type II tubules have the potential to be shaped by cytoplasmic microfilaments and microtubules. However, tubules in a type I reticulum, as we describe for *C*. *elegans* intestinal nuclei, are not connected with the cytoplasm. Here, we found that the type I tubules in intestinal nuclei frequently contact, and extend parallel to, nMFBs (Fig 3I). Moreover, unidentified vesicles that contact nMFBs extend long protrusions parallel to nMFBs, which might contribute to tubule growth (Fig 3J).

### nLDs in intestinal cells and aging

If nLDs contribute to nuclear damage in intestinal cells, they have the potential to impact aging. Several studies have implicated the intestine, the largest organ and major metabolic tissue in *C*. *elegans*, as having a critical role in determining lifespan. The intestine undergoes considerable deterioration as animals age, including the loss of nearly one third of intestinal nuclei [102]. Moreover, the final stage of aging, death, is associated with a rapid, cascading necrosis of the intestine [103]. Interestingly, previous studies noted that nuclear defects in old adults appeared most severe in the anterior half of the intestine [102], where we see the highest concentration of nLDs and nuclear defects in young adults. We found that all intestinal cells appear to initiate large changes in metabolism at the start of the self-fertile period, including large decreases in yolk granules and stored glycogen (S1 Fig). However, only the subanterior cells appear to undergo large changes in fat, as stored fat is severely depleted and later replenished (Fig 1H).

While *C*. *elegans* lifespan appears to be decreased by intestinal defects, lifespan can be extended by altering gene expression specifically in the intestine, and doubled by downregulation of an insulin-like signaling pathway [104]. Lifespan extension is mediated by the FOXO transcription factor DAF-16, and the essential focus of DAF-16 activity is in the intestine [105]. The idea that events in early adulthood contribute to later aging is supported by a recent, detailed examination of several senescence-associated pathologies In *C*. *elegans* [106]. Although the incidence of the various pathologies peaked in adult populations at D10-D12 (present timescale), the first cases occurred much earlier, near the end of the self-fertile period. Moreover, the same study showed that the rewiring of intestinal metabolism to support yolk production was a driver of senescence. In future studies, identifying genes that differentially regulate fat mobilization in the subanterior region of the intestine might provide important tools to dissect the roles of the intestine in aging. For example, expression studies have shown that several microRNAs have region-specific expression in the anterior or posterior of the intestine, although the functional significance of this asymmetry is not known [107].

### Germ cells appear to tolerate nLDs

In marked contrast to the evident damage in intestinal nuclei during the self-fertile reproductive period, germ cell nuclei appear relatively unchanged during these stages. At least 10–15% of wild-type germ cells contain nLDs before becoming oocytes, as do the vast majority of germ cells in *seip-1(zu483)* mutants (Figs 13B and 15E). The relative sizes of nLDs are often much larger in germ cells than in intestinal cells, and the largest nLDs in *seip-1* and *nemp-1* mutant germ cells are nearly half the nuclear diameter. Nevertheless, nLD-containing germ cells were not noticeably enriched among the total population of apoptotic cells (Fig 15F and 15H). Moreover, nearly all germ cells appear to produce normal embryos when the apoptosis pathway is blocked in *ced-3* mutants and in *ced-3;seip-1* double mutants (Fig 15I). These results suggest that nLDs can occupy a large volume of a germ cell nucleus without compromising nuclear functions.

Why might nLDs damage intestinal nuclei but not germ nuclei? Germ cell nLDs occur primarily in the pachytene stage of meiotic prophase, when the paired chromosomes are highly active but structurally compact. Pachytene germ cells have little apparent peripheral heterochromatin (Fig 15B), and germ cell nLDs never have heterochromatin coats (Fig 10F). Loops of transcriptionally active chromatin are thought to radiate from the synaptonemal complex between the paired pachytene chromosomes, as in other systems, but little is known about the size or three-dimensional organization of the loops. However, any chromosome territories in *C*. *elegans* germ cells would likely be limited by nucleoli, which occupy most of the nuclear volume (Fig 10B).

Lamin sacs occur frequently in intestinal nuclei (Fig 6D), but are very rare in germ cells. Our results suggest that lamin sacs in intestinal nuclei arise by the combined inpocketing of the INM, the lamina, and the peripheral heterochromatin (Fig 6F and 6G). By contrast, nLDs forming in germ cells appear to slip through the lamina, and possibly trigger formation of a lamin patch (Fig 15C). We speculate that the lamin patch might represent a germ cell-specific repair of the nuclear lamina; for example, lamin becomes enriched at "scars" that form at sites of nuclear rupture in human cells [108].

### COPI and germ cell nLDs

Because the goal of this study was to examine the effect of nLDs on nuclear integrity, we did not attempt to develop a genetic pathway for nLD formation. Nevertheless, the finding that nLD phenotypes result from mutations in an INM-localized protein and a known regulator of lipid droplets conforms with the general understanding of how and where nLDs form. By contrast, the apparent role of COPI in nLD formation was surprising. Some COPI components appear to have a role in regulating fat synthesis, such that their depletion increases cLD sizes [16,17]. However, COPI vesicles are best known for their role in retrieving mislocalized ER-resident proteins, and our results suggest that nLD phenotypes result from retrieval defects. We found that semidominant mutations in two COPI subunits, COPA-1/α-COP and COPB-2/β’-COP, markedly increase the number of nLDs in germ cells, but not in intestinal cells. α-COP and β’-COP are related subunits that function specifically in binding the retrieval motifs of ER-resident membrane proteins, and the mutations described here are near critical residues in the binding pockets (S10 Fig). Because ER stress can contribute to nLD formation in hepatocytes [45], one possibility that we cannot exclude is that the COPI mutations influence nLD formation indirectly, through ER stress. However, we found that single amino acid substitutions in the predicted retrieval motifs of ER-resident membrane proteins involved in fat synthesis also caused nLD phenotypes, either individually or in combination (Fig 14G). Thus, we favor a hypothesis that the COPI mutants have nLD phenotypes because enzymes involved in fat synthesis are mislocalized to the INM.

The broad outlines of the pathways that membrane proteins take to the INM are understood. Two conserved systems exist for inserting nascent polypeptides into membranes, and those complexes are localized to the ER membranes and to the ONM [109]. Membrane proteins inserted into the ONM have the potential to diffuse laterally in the membrane, through nuclear pores and onto the INM (Fig 5C). Once on the INM, proteins can be retained selectively by interactions with nuclear components such as lamin and chromatin. Pachytene germ cells present an interesting variation from more typical cells, as the nucleus is largely segregated from most common cytoplasmic constituents: The cytoplasm around a pachytene nucleus contains very little ER or membrane vesicles, essentially no cLDs, and by TEM only rarely appears to contain Golgi (S8 Fig). Instead, these organelles are concentrated in the gonad core, at least in part by the kinesin-mediated trafficking reported here (Fig 12). Conversely, pachytene cells contain a remarkably dense concentration of ribosomes, both in the cytoplasm (Fig 12D) and on the ONM (panel 2, Fig 15B). These combined features suggest that newly exported mRNAs in germ cells are likely to encounter ribosomes, and begin translation, before reaching the gonad core. Thus, nascent membrane proteins might insert into the ONM before encountering an ER membrane. If so, we speculate that there might be a variant COPI pathway that removes ER-resident membrane proteins from the ONM, allowing these proteins additional opportunities to reach the abundant ER in the gonad core. The relative lack of ER in pachytene germ cells might also be expected to increase the numbers of soluble ER-residents that are inserted into the perinuclear cistern of the nuclear envelope, rather than into the lumen of the ER. Interestingly, an antibody that recognizes the HDEL retrieval motif for soluble ER-residents stained germ cells asymmetrically, near the core-facing surfaces of nuclei (inset, Fig 12B). This asymmetry required kinesin, and supports a hypothesis that germ cells have mechanisms to deliver both membrane and soluble ER-residents to the main body of ER in the gonad core.

In summary, our study suggests that nLDs are a widespread, important feature of nematode biology that might impact both aging and survival. Previous forward genetic and RNAi screens in *C*. *elegans* and *Drosophila* studies have identified hundreds of genes involved in lipid storage and metabolism; these genes have diverse functions, including roles in feeding, energy utilization, TAG synthesis, and lipolysis [110]. We showed here that the *C*. *elegans* gonad provides an excellent system to rapidly identify and study genes with relatively specific roles in nLDs. By contrast with the simplicity of germ cells, intestinal nLDs occur within, or induce, a complex and remarkable environment of nuclear tubules and microfilaments that warrants further study. For example, nMFBs might have general roles in nuclear maintenance, and only recognize nLDs as one of multiple objects to be removed. Similarly, the nuclear tubules might have primary functions related to nucleoli, megaRNPs, or repair, and only inadvertently provide membrane platforms for nLD growth.

## Methods

### Worm stains and culture conditions

General nematode culture was as described [113]; all strains were derived from the wild-type Bristol strain N2 and grown at 20°C unless stated otherwise. *E*. *coli* strain OP50 was used for all experiments. Plate media was made using Bactopeptone (Difco) and Bacto-agar (Difco) as described [113]. After pouring, plates were allowed to dry at room temperature for 2 days, then seeded with a fresh, overnight culture of OP50. Plates were kept in the dark at room temperature, and used 2–5 days after seeding. Worm cultures were grown for a minimum of two generations without starving before analysis, at which time 30–50 mid L4 worms were collected onto 6 cm plates and grown to the desired stage; with these culture conditions we observed lifespan curves and maximum lifespans (23 days from L4) that were consistent with published reports [114]. In the course of this study we observed some phenotypic variation between our N2 cultures and those obtained from other laboratories using differently sourced media, although the basis for the variation was not determined. The variation included apparent mitotic defects in the distal gonad, larger and more variable nLDs, and adult intestines that appeared to lack many of the normal postembryonic nuclear divisions.

The mutant alleles, strains, and fluorescent reporters used in this study were as follows and obtained from the *Caenorhabditis elegans* Genetics Center unless noted otherwise:

- **alleles: LG I:** *fog-1(q253ts)*. **LG II:** *csp-1(n4967)*, *daf-22(m130)*, *maoc-1(hj13)*. **LG IV:** *ced-3(n717)*, *ced-3(n3692)*, *klc-1(ok2609)*. **LG X:** *dhs-28(hj8)*, *sams-1(ok3033)*.

The lipid droplet mutants *drop-1(ss9)II*, *drop-4(ssd206)*, *drop-7(ssd75)*, *drop-8(ssd89)*, *and prx-10 (ssd68)* were a gift from Shaobing Zhang, *seip-1(tm4221null)* was a gift from Shohei Mitani. *seip-1(syb2121)* was purchased from SunyBiotech.

- **strains: JM149** (GFP:HIS2B; gift from Jim McGhee), **RW10062** (HIS-24:-mCherry), **VC41030** (contains *copb-2(gk936767)*), **VC40959** (contains *copb-2(gk900572)*. The following *C*. *elegans* wild strains were used: **CB4856**, **CX11314**, **DL238**, **ED3017**, **EG4725**, **LC34**, **JT11398**, **JU258**, **JU775**. The transgenic strain expressing SPCS-1/SP12:GFP was a gift from Anne Spang.

### Antibodies

The following antibodies were used: ATP synthase beta (ab14730 Abcam), anti-fibrillarin (38F3, Abcam), anti-HDEL (2E7, Sigma), anti-HIM-4 [115] (gift from Bruce Vogel), anti-lamin (LMN-1) [27] (gift from Yosef Gruenbaum), anti-NPP-9 [116], anti-α-Tubulin (YOL3/4 Abcam).

### Immunostaining

All dissections were done as described [38]. Briefly, dissections were done on glass microscope slides that were coated with tape to provide a hydrophobic, soft surface. Invisible tape (single layer, matte finish) was applied to a glass microscope slide, then the slide was heated to 300°C for about 10 mins; the edges of the tape should appear slightly melted. The slides were wiped briefly with 95% EtOH, then dH20, before use. A drop of 30–50 μl of M9 buffer was placed on the taped slide, and 20–30 worms were picked from a culture plate directly into the drop. The worms were agitated briefly to remove any residual bacteria, and washed in two changes of M9 buffer. The M9 buffer was removed and replaced with 30 μl of gonad buffer [48% Leibovitz L-15 (GIBCO), 9.7% Fetal Calf Serum (GIBCO), 1% sucrose, 2 mM MgCl2; adjustments to the osmolality were necessary with different stocks of Fetal Calf Serum, and determined by examining live gonads under a compound microscope for shrinkage or swelling]. Worms were dissected at the posterior bulb of the pharynx to expose the anterior half of the intestine, and at the rectum to expose the posterior half of the intestine; both modes of dissection were used for gonad analysis. Following dissection, the 30 μl of gonad buffer was mixed with an additional 30 μl of 2X fix [5% formaldehyde (Sigma), 25 mM HEPES (7.4), 40 mM NaCl, 5 mM KCL, 2 mM MgCl2]. Tissues were fixed for 15–25 mins, rinsed in phosphate-buffered saline (PBS) (137 mM NaCl, 2.7 mM KCl, 10 mM Na2HPO4, 1.8 mM KH2PO4 at pH 7.4), permeabilized with 0.3% Triton-X100 in PBS for 10 mins at room temperature, then rinsed with several changes of PBS. For experiments that compared two sets of animals, each set was dissected separately to leave an identifying body part attached to the tissue of interest. After fixation, both sets were mixed for permeabilization and subsequent processing. Tissues were transferred in PBS to teflon-coated, multi-well test slides (Tekdon) for immunostaining. Staining in primary antibody was overnight at 4°C, staining in secondary antibody was for 1 hr at room temperature. For mounting, coverslips were supported with precision-sized glass beads (Whitehouse Scientific) that were slightly smaller than the diameter of the tissue of interest; for example, 22 μm and 28 μm beads were used form most D1 and D2 adult gonads, respectively.

### RNAi

Worms were exposed to gene-specific dsRNA by feeding as described [117]. Worms were placed on the RNAi plates as L4 larvae and scored as D2 adults; the empty feeding vector L4440 was used for control experiments.

### Lipid staining

For fluorescent staining, tissues were fixed in formaldehyde and permeabilized with detergent as for immunostaining. Tissues were stained in 1 μg/ml BODIPY 493/503, LipidTOX red (1:200 dilution; Invitrogen), or Nile red (1 μg/ml; Sigma), for 30–60 mins at room temperature. For Oil Red O staining, intestines or gonads were fixed in formaldehyde as above for 45 mins, then 2.5% glutaraldehyde/PBS for an additional 45 mins. The additional glutaraldehyde fixation was used to prevent artificial and variable fusion between lipid droplets that was observed in stained tissues fixed only with formaldehyde. Tissues were rinsed several times in dH20, then stained with a fresh solution of 60% Oil-Red-O prepared as described [118]. Images were acquired with a DeltaVision microscope and processed using deconvolution software (Applied Precision). Images were exported to Adobe Photoshop for contrast/brightness adjustments, reorientation and cropping. Other images were acquired with a spinning disk confocal microscope [Hamamatsu C9100 camera on a Nikon TE-2000 inverted microscope equipped with a Yokogawa CSU-10 spinning disk; image acquisition was with Volocity 5.3.3 (Improvision) software]. Orthogonal projections of optical Z-stacks were generated and analyzed either with Volocity software or with ImageJ.

For quantification of cLD sizes in the gonad core and intestine, 15 μm optical z-stacks (0.2 μm sectioning thickness) were acquired at 100X. The region analyzed in the gonad core corresponded to the peak zone of nLDs, and the int2 cells were analyzed in the intestine. The z-stacks were deconvolved with AutoquantX3 software, then imported into Imaris 8.0 for analysis.

### Electron microscopy

Intestines or gonads dissected as above were transferred to fixative [2.2% glutaraldehyde, 0.9% paraformaldehyde, 50mM cacodylate (pH 7.2), 90mM sucrose, 0.9 mM MgCl2]; most of the fixative was removed immediately and replaced with fresh fixative; tissues were fixed for a total of 3 hours at room temperature, with an additional change of fixative at 1.5 hours. Fixed tissues were rinsed briefly in 1% sucrose in 50mM cacodylate (pH 7.2), then postfixed on ice for 30 mins in a solution of 1% osmium (EM Sciences), 50mM cacodylate (pH 7.2), and 0.8% potassium ferricyanide. Tissues were washed twice briefly at room temperature in 1% sucrose in 50mM cacodylate (pH 7.2), then washed in three changes of 50mM cacodylate (pH 7.2) for 5 min each. The tissues were then treated with 0.2% tannic acid (Malinkrodt) in 50 mM cacodylate (pH 7.2) for 15 mins at room temperature, then rinsed several times in H20. Unnecessary body fragments were removed at this stage, and the final, fixed tissues were encased in agar as follows. The tissues were transferred in minimal liquid to a 9 cm petri plate containing 6 mls of a solidified solution of 1% Bacto-agar (Difco) in H20. The tissues were clustered into a compact monolayer using a fine, drawn-out capillary glass or an eyelash affixed to a toothpick, with care taken not to break the agar surface. A cluster containing 20–30 half-intestines, or similar numbers of gonad half-arms, was prepared for each stage examined. The clustered tissues were linked together by carefully adding a small drop of 0.15% poly-L-lysine (Sigma P8920) near the cluster, allowing the drop to spread gently into the cluster, and then immediately removing all excess liquid. A small drop of glutaraldehyde fixative [2.2% glutaraldehyde, 0.9% paraformaldehyde, 50mM cacodylate (pH 7.2), 90mM sucrose, 0.9 mM MgCl2] was added to the clustered tissues for 15 mins. To cover the clustered tissues with agar, a solution of 1% Bacto-agar/H20 was backfilled into a drawn out, sealed pasteur pipet and allowed to cool as much as possible while remaining molten. The sealed tip of the pipette was broken to allow the passage of small drops of agar, and one drop was placed directly over the tissue cluster. The encased tissue was allowed to harden for at least 30 mins at 4°C. A minimal block of tissue was excised and immersed briefly in H20, then transferred to a watch glass containing 2% aqueous uranyl acetate (Ted Pella) for 2 hrs at room temperature. The block was rinsed in three changes of ddH20, 10 mins each, then dehydrated in a graded acetone series and embedded in Spurr’s resin (Electron Microscopy Sciences) following standard procedures [119]. Specimen blocks were sectioned by collecting 6–10 serial thin sections (70nm each), discarding the next 0.5 um, then repeating the sequence. Sections were collected on Formvar/carbon-coated 200 mesh grids (Ted Pella, Inc). For staining, grids were floated individually, section side down, on single drops of freshly-prepared Reynold’s lead citrate staining solution (Ted Pella, Inc.) for 2 min at room temperature. Grids were washed thoroughly with ddH2O and blotted on filter paper, then air dried. Specimens were examined with a JEM-1400 transmission electron microscope operated at 120kV and photographed with a Gatan Rio 4k x 4k camera.

### Centrifugation

Live worms suspended in 300 μl of M9 buffer were placed in 3.5 ml polycarbonate centrifuge tubes (Beckman Coulter 349623) in a swinging bucket rotor pre-cooled to 12°C (MLS-50, Beckman Coulter). The worms were centrifuged for 50 mins at 12°C in an Optima Max-XP Ultracentrifuge (Beckman Coulter). The centrifugation speed was increased gradually as follows to maintain worm viability: 500g, 1000g, 2000g, 3000g, 4000g, and 5000g for 10 mins each. The worms were recovered and transferred to seeded plates.

### Mutant isolation and cloning

Worms were mutagenized with 50 mM ethyl methanesulphonate (EMS) as described [113]. A total of about 15,000 F2 progeny of the mutagenized worms were screened in four separate experiments. Live worms were clustered on microscope slides and screened visually for nLD phenotypes; anesthesia was not necessary or used as the clustered worms rapidly became hypoxic and stopped moving. Worms were examined initially at 40X magnification on a Zeiss compound microscope equipped with DIC optics, then candidates with possible nLD phenotypes were examined further at higher magnification and recovered. The *zu482*, *zu483*, *and zu501* mutants were each outcrossed 8X or 10X (*zu501*), then mapped by standard two-factor and three-factor crosses. *zu482* was mapped further using chromosomal deficiencies, and shown to be close to the *pop-*1 gene on LGI in a non-overlapping region between tDf4 and tDf3. Whole-genome sequencing of *zu482* showed this region contained a unique missense mutation in the *copa-1* gene. The identification of *copa-1(zu482)* as the mutation responsible for the nLD phenotype was further validated by the finding that an independently isolated strain, *copa-1(zu511)*, had the identical mutation and resulted in an indistinguishable nLD phenotype. To address whether *zu511* might be a contaminant from the *zu482* stock, we used the genome sequence of *zu482* to identify a non-coding DNA polymorphism about 17kb from *zu482* (TCTTC deletion, position 2878435). The corresponding region in the *zu511* strain was amplified by PCR and sequenced, and did not contain the polymorphism. *zu483* mapped between -0.5 and -0.7 on LGI, close to the *dpy-11* gene. A small chromosomal deficiency, sDf26, failed to complement *zu48*3, and the deficiency endpoints indicated that *zu483* was between positions 5579194–6318973. This region contained two candidate genes encoding the lipid regulator SEIP-1/seipin and the map kinase-associated membrane protein JAMP-1. Both genes were amplified by PCR and sequenced, and the only mutation was in *seip-1*. Crispr-Cas9 gene editing was used to generate the *seip-1(zu520)* mutation, which has an nLD phenotype closely resembling *zu483*. The *zu501* mutant was mapped very close to the *unc-45* gene on LGIII. Whole-genome sequencing showed that the *zu501* strain had a nonsense mutation in the *nemp-1* gene, about 19 kb from *unc-45*, but that the nearest additional coding mutations were 384 kb and 668 kb from *unc-45*. We used Crispr-Cas9 gene editing to reproduce the *zu501* mutation in otherwise wild-type animals, and found that the resulting *nemp-1(zu522)* animals appeared identical to *nemp-1(zu501)*.

### Larval growth rate

L4 adults from wild-type and mutant strains were collected and allowed to develop for 40 hours at 20°C. At that time, eggs were collected in M9 buffer and allowed to hatch for two hours. Multiple pools of about 150 of the hatched, synchronous L1 larvae were collected onto separate test plates for the t = 0 hr timepoint. At 45 hours and 1 hour intervals thereafter, adults with eggs were counted and removed.

### Genome editing

Crispr-Cas9 genome editing was as described [120].

### Genome sequencing

Genomic DNA was prepared (Truseq DNA kit) and fragmented using a Covaris LE220 focused-ultrasonicator (Covaris, Inc., Woburn, MA, USA). Sequencing libraries were prepared with KAPA Hyper Prep Kit (Roche, Basel, Switzerland). Libraries were then barcoded, pooled, and sequenced on an Illumina MiSeq with a paired-end 150 bp read configuration, generating 6.2–6.7M read pairs for each sample. On-instrument secondary analysis was performed with MiSeq Reporter Software v2.5.1 (Illumina, Inc.) using base calls and quality scores generated by Real-time Analysis (RTA) v1.18.54 (Illumina, Inc.). BWA mem 0.7.12 [121] was used to align reads for each sample to the *C*. *elegans* reference genome WBcel235. The resulting alignments were converted to coordinate sorted BAM format with SAMtools 1.10, and evaluated for PCR duplicates using Picard 2.7.1 [http://broadinstitute.github.io/picard]. GATK 3.7 [122] was used to further refine alignments and to call variants with the HaplotypeCaller. SnpEff 4.3t [123] was used to annotate variants according to WBcel235 reference data (snpEff_v4_3_WBcel235.86). Filtering with bcftools 1.8 [http://www.htslib.org/] selected high quality calls (QUAL > 100), homozygous alternate in one of the two samples, and at least five reads supporting each variant.

## Supporting information

S1 Fig Stage-dependent changes in intestinal cytoplasm. (A-C) TEM of intestinal cell cytoplasm in L4, D2, and D3 hermaphrodites; cLDs are tinted green. L4 cells have large numbers of cLDs, yolk granules (large, electron-dense bodies) and islands of glycogen (magenta); the inset shows the distinctive shapes of glycogen rosettes (arrowhead). By the D2 stage, all cells have lost large amounts of yolk granules and glycogen, but subanterior cells show the greatest loss of cLDs. Note the increase in gut granules, which can be distinguished from yolk granules by their distinctive staining and morphology. mito = mitochondria.
(TIF)

S2 Fig nLD identification in intestinal cells. (A) The image shows the surface of an intestinal nucleus stained for lipid (green, BODIPY) and DAPI. The lipid droplet is a cLD that is partially embedded in the nuclear envelope. The inset shows that even large cLDs such as this do not create artificial, ring-like patterns in the peripheral heterochromatin. Thus, the presence of a heterochromatin ring or coat can be used to distinguish an nLD from an envelope-embedded cLD, even without immunostaining for the envelope. See **S7 Fig** for an example of a germ nucleus with an envelope-embedded cLD. (B) Examples of small nLDs with heterochromatin coats in intestinal cells that were stained for lipid (green, BODIPY) and DNA (DAPI staining shown in red for contrast), but that were not detergent permeabilized; the insets show the heterochromatin rings (white) separately. Note that multiple nLDs are visible on different focal planes of single nuclei. A comparable frequency of nLDs was observed in our TEM analysis (compare panel 3 in **Fig 2F**), but is higher than observed in immunostaining experiments with detergent permeabilization. Moreover, some nLDs as in panel 2 were smaller than any nLDs detected in detergent-permeabilized cells. (C) Examples of large nLDs with heterochromatin coats. Note neighboring regions of the envelope that appear deficient in heterochromatin (arrowheads). (D) D2 nucleus with an apparent lamin-deficient region at the base (arrowhead) of a large nLD. The overexposed lamin channel (inset) shows that the lamina appears to be continuous. (E) TEM images of D1 and D2 nuclei showing variation in the shapes and staining patterns of some nLDs; similar variation also was observed for cLDs. Note that each of the nLDs has an electron-dense coat.
(TIF)

S3 Fig TEM of nLDs and type I tubules in the nucleoplasmic reticulum of intestinal nuclei. (A) Examples of type I nuclear tubules (arrows) that appear to extend between the envelope and the nucleolus. Note the cysts and kernel vesicles (black arrowheads) in the nuclei. (B) Images showing the varied shapes of nuclear tubules, including zig-zag patterns (panels 5–8). Panels 2 and 4 show electron-dense granules in the perinuclear cistern (black arrowheads). Panels 9–11 show examples of tubules (arrows) associated with nLDs; note the apparent membrane fragments at the perimeter of the nLDs (white arrowheads).
(TIF)

S4 Fig TEM of kernel vesicles and presumptive degraded vesicles. (A) Variation in the appearance of kernel vesicles in D1 and D2 nuclei. Note the absence of nuclear pores (white arrowheads) at the base of each kernel vesicle. (B) Examples of membrane-enclosed, degraded material at or near the envelope of D4 nuclei. Panels 1 and 2 show protrusions (arrowheads) of the ONM that appear to contain membrane fragments and other debris. Note that the protrusion in panel 2 is adjacent to multiple nuclear tubules (arrow). Panel 3 shows a D4 nucleus adjacent to a large, membrane-enclosed vesicle (arrow), that appears to be filled with debris (inset).
(TIF)

S5 Fig TEM of L4-D2 intestinal nuclei. Representative images of L4, D1, and D2 nuclei from the int1, subanterior, and posterior regions of the intestine as indicated. The L4 and D1 nuclei generally have relatively clear nucleoplasm, although a few D1 nuclei have kernel vesicles (arrowheads and inset). More kernel vesicles are apparent in D2 nuclei, along with tubules (tub), nuclear microfilament bundles (nMFBs) and a few cysts. A secondary nucleolus is visible in one of the D2 subanterior nuclei.
(TIF)

S6 Fig TEM of D3-D4 intestinal nuclei. Representative images of D3 and D4 nuclei from the regions of the intestine indicated, labeling as for **S5 Fig**. Note that several D3 subanterior nuclei have clumps of electron-dense material in the nucleoplasm that are not generally present in int1 nuclei or most posterior nuclei, and are not present in L4-D2 nuclei (**S5 Fig**).
(TIF)

S7 Fig Basic characterization of germ cell nLDs. (A) These experiments address the specificity of the lipid stains for germ cell nLDs. Studies on somatic fat in live *C. elegans* found some commonly used lipid dyes stained lysosome-related organelles in addition to lipid droplets [124]. Panel 1 shows that nLDs visible by DIC in fixed tissues also stain with BODIPY, panel 2 shows that BODIPY-stained nLDs also stain with LipidTOX, and panel 3 shows that *glo-4* (ok623) mutants contain BODIPY and Nile Red-stained nLDs; the *glo-4* mutants lack cytoplasmic lysosome-related organelles that can stain with lipid dyes. n = 18–25 gonads for each experiment. (B) Panel 1 shows an example of an envelope-embedded cLD in a germ nucleus. These are rare, likely from the lack of cLDs in most germ cells, but need to be distinguished from nLDs. Panel 2 shows an example of orthogonal planes used to verify an nLD (asterisk) is entirely within the envelope. (C) This experiment addresses whether yolk is a determinant of nLD formation. The image shows a *rme-1(b1002)* mutant gonad stained for the envelope (red, NPP-9/RanBP2), lipid (green, BODIPY), and F-actin (magenta, phalloidin). These mutants are unable to take up yolk lipoproteins, but appear to have normal numbers of nLDs (arrowheads) in the peak zone (panel 2; n = 24 gonads). The *rme-2* mutants often have what appear to be giant cLDs around germ nuclei (arrows in panel 3). However, inspection of the germ cell membranes showed that this lipid is between, but outside of, germ cells. We presume this material consists of yolk lipoproteins that accumulate in the body cavity in *rme-2* mutants. (D) Calibration of temperature-equivalent worm ages based on egg production. The plot shows that 20°C animals analyzed as D1 adults (24 hours post-L4) have produced an average of 50 eggs at the point of analysis. Thus, we compared 20°C D1 adults with 15°C adults that have produced similar numbers of eggs, which the graph shows occurs at 44 hours post-L4. Similarly, we compared 20°C D1 adults with 25°C adults that are 22 hrs post-L4. For the 20°C D2 comparison (48 hrs post-L4), we used 15°C adults that are 84 hrs post-L4, and 25°C adults that are 36 hrs post-L4; the 25°C time point was adjusted to allow for the cessation of egg laying. (E) Plots comparing the temperature dependence of nLDs in wild type, *seip-1*, and *copa-1* mutants, as described in the text and calibrated as above. Horizontal brackets/asterisks compare D1 and D2 wild-type adults at different temperatures. Vertical, grey asterisks compare the mutants with wild type at the same stage/temperature. The following numbers of nLDs were scored at (20°C, 15°C, 25°C): WT (59,29,64), *seip-1* (112, 129, 128); *copa-1*(196,129,137). (F) Plots comparing the temperature dependence of nLD size in wild type, *seip-1*, and *copa-1* mutants, as described in the text. Vertical, grey asterisks compare mutants with wild type at the same stage/temperature. Plot shows mean and standard deviation. P value from unpaired t test with Welch’s correction **** P< 0.0001, ***P ≤ 0.01, **P ≤ 0.01, n.s. not significant.
(TIF)

S8 Fig Germ cells have relatively little ER and rarely contain Golgi. (A) The left column shows TEM images of a pharyngeal cell, a gonad sheath cell, a germ cell, and an intestinal cell, all at the same magnification. The black outlines in the diagrams at right indicate the nuclear membranes and ER membranes. Note that germ cells have little ER compared to any of the somatic cell types, and that the nuclear envelope is by far, the major ER subdomain in germ cells. (B) Examples of rare, presumptive Golgi stacks in germ cells; only two such examples were found in over 2000 germ cells examined by TEM. Pg = P granule, mito = mitochondria.
(TIF)

S9 Fig nLDs are a common feature in germ cells of *C. elegans* wild strains and mutants. (A) DIC images of gonads in *C. elegans* wild strains or mutants as indicated; the region shown corresponds to the peak zone of nLDs in wild-type gonads. (B) Qualitative, 6-bin scale (top) comparing the nLDs and cLDs in N2 wild-type hermaphrodites with those in the strains and mutants listed. N2 is the laboratory strain of *C. elegans*; other wild strains are indicated in blue. Mutants isolated in previous studies but not analyzed for nLDs are indicated in green and referenced in the text. 20–30 live, anaesthetized animals were scored for each strain. Asterisks indicate wild strains or mutants where individual germ nuclei appeared to contain multiple nLDs more often than observed in N2 wild-type hermaphrodites. var = variable. (C) Gonad from a *seip-1(tm4221)* null mutant stained for lipid (green, BODIPY) and lamin (red, LMN-1). The image at left is a 5 μm maximum intensity z-projection through the top nuclear level; the few lipid droplets visible are all cLDs; compare with similar optical plane through a *seip-1(zu483)* gonad in Fig 15H. The panel at right is a 5 μm maximum intensity z-projection through the gonad core, showing relatively abundant cLDs. Scale bar in microns, as labeled.
(TIF)

S10 Fig The dibasic retrieval motif and COPI proteins in *C. elegans*. (A) The dibasic retrieval motif has not been studied in *C. elegans*, but several proteins appear to contain the motif. Shown here are predicted ER-resident membrane proteins in *C. elegans* (see text) with C-terminal peptides that (1) are conserved in other *Caenorhabditis* species, and (2) conform with the dibasic peptide retrieval motifs KKxx and RKxx. The proteins SPCS-1, TRAP-1, TRAP-2, TRAM-1, and NRA-4 have been confirmed as ER proteins [125,126]. Y37E11AM.3 is an ortholog of human FVT-1/KDSR (3-ketodihydrosphingosine reductase), which has been localized to the ER [127]. DPY-11 has been localized in *C. elegans* to subcellular membranous organelles consistent with the ER [128], and the human ortholog, TMX, has been localized to the ER [129]. (B) Alignment of the ER-resident membrane protein SEIP-1/seipin in *C. elegans* and other *Caenorhabditis* species. The gray bars indicate predicted membrane-spanning domains. Note that the cytoplasmic domain, after the second membrane-spanning domain, shows little conservation other than the C-terminus, an RKxx-type retrieval motif. (C) N-terminal half of COPA-1/α-COP and COPB-2/β’-COP in *C. elegans* aligned with the homologous human and *S. pombe* sequences; WD40 repeats are indicated in magenta. Residues that form the binding pocket for dibasic retrieval peptides in ER-resident membrane proteins are indicated by black circles, and two critical acidic residues are indicated by arrows [data from [77]]. The *copa-1* and *copb-2* mutations described in this paper are indicated, with amino acid substitutions shown in red. The mutation *copa-1(zu511)* was isolated in a different screen than *copa-1(zu582)*, but causes the same amino acid substitution. Sequence data compiled from Wormbase WS270 (https://wormbase.org).
(TIF)

S11 Fig Phenotypic analysis of nLD mutants. (A) *nemp-1(zu501)*. *nemp-1* null mutants were described recently by others and shown to cause variable defects in fertility, brood size, and egg viability [72]. The semi-dominant *nemp-1(zu501)* mutant produces some dead eggs, but is homozygous viable and appears superficially healthy with normal body morphology and growth rates. *nemp-1(zu501)* intestines generally resembled wild type; for example, cLDs appeared normal and some nuclei contained nLDs (Table 1). At the D2 stage, fat was depleted predominantly from the subanterior region, and int1 nuclei at the D2 stage (panel 1; arrowheads indicate lamin lines) appeared rounder and "healthier" than subanterior nuclei (panel 2; n = 28 intestines). However, several subanterior nuclei (panel 2) contained far greater numbers of lamin sacs than observed in wild type, suggesting either a defect in sac removal or in events that trigger sac formation. *nemp-1* gonads superficially resembled wild type, except for the enlarged nLDs (panel 3; n = 28 gonads). Germ nuclei appeared to increase in size uniformly as they progressed through pachytene, the gonads had small numbers of binucleate (T-bar) and mononucleate apoptotic cells, and no nLDs were present in sheath cell nuclei (sh nuc). D2 gonads could accumulate variable, and sometimes large, amounts of intercellular lipid (presumably yolk lipoproteins) between germ cells, as observed with *rme-2(b1002)* mutants (see S7 Fig). However, 7/28 *nemp-1(zu501)* gonads had excessive numbers of binucleate cells (panel 4); nLDs could be present in one, both, or neither of the germ nuclei (inset). In addition, several *nemp-1(zu501)* gonads had a few germ nuclei with either larger, or fewer, numbers of chromosomes than wild type, suggesting a defect in mitosis. Because nLDs are not found in mitotic cells, and most nLDs form after binucleate cells develop [38], we consider it unlikely that nLDs cause the mitotic and binucleate cell phenotypes in *nemp-1(zu501)* mutants. (B) *copa-1(zu482)*. *copa-1* null mutants are inviable [130], and *copa-1(RNAi)* causes a reduction in germ cell numbers [131]. The semidominant *copa-1(zu482)* mutant is homozygous viable and appears morphological normal and healthy. Fat appeared to be depleted preferentially from the subanterior region of D2 intestines, as in wild type, but intestinal cells appeared to contain slightly higher levels of fat than wild type. We examined 272 D1, and 411 D2, intestinal nuclei in *copa-1(zu482)* mutant adults. Despite having a large increase in nLDs in germ cells, the mutant intestinal nuclei appeared to have markedly fewer nLDs than wild type (Table 1). Moreover, the rare nLDs observed were very small, and no nuclear ruptures were observed. *copa-1(zu482)* mutants appeared to have relatively few lamin sacs in intestinal nuclei: Panels 1 and 2 show maximum intensity z-projections of the int1 nuclei and some subanterior nuclei; the arrowhead indicates a lamin line. Most D1 and D2 *copa-1(zu482)* nuclei lacked cysts, consistent with the hypothesis that cysts originate from lamin sacs. *copa-1(zu482)* male germ cells generally lacked nLDs, similar to wild type (see S9 Fig), but appeared less effective at mating than either wild-type males or *seip-1(zu483)* mutant males. For this experiment, single D1 wild-type or mutant males were mated to a single L4 wild-type hermaphrodite for 48 hrs and then removed. Mating efficiency was scored by the presence of male cross-progeny. Wild type and *seip-1(zu483)* males were equally efficient at mating (68.0% vs 70.7%; n = 50,59 crosses), but only 41.2% of *copa-1(zu482)* males produced cross-progeny (n = 63 crosses). By TEM, most nLDs in *copa-1(zu482)* germ cells resembled wild type; for example, all of the earliest nLDs were adjacent to the envelope (panel 3, n = 78 nLDs), and 14/48 nLDs had bristle coats. However, *copa-1(zu482)* germ cells often contained more than one nLD, as observed in staining experiments. Some nLDs had hourglass shapes (inset, pane 3), which were never observed in wild type germ nuclei, and were not observed in a much larger sample set of *seip-1* mutant nuclei. (C) *seip-1(zu483)*. This figure addresses the identity of giant, non-nuclear lipid droplets that occur frequently in *seip-1(zu483)* gonads (arrowheads in panels 1 and 2), but that are not seen in wild type. These lipid droplets superficially resembled the large intercellular deposits of lipid observed in *nemp-1(zu501)* and *rme-2(b1002)* gonads. However, 3D tracing of phalloidin-stained membranes (panels 1, 2) and TEM (panel 3) showed that the lipid bodies in the *seip-1* mutants were within gonad sheath cells (outlined or tinted green in panels 1–3; gc = germ cell). The nucleus in the engulfed, apoptotic germ cell in panel 3 has partially fragmented, but can be identified by the large nucleolus (No). Sheath cells are much larger than germ cells, and can engulf several apoptotic germ cells simultaneously (bracket in panel 2). Thus, *seip-1(zu483)* sheath cells might acquire abnormal amounts of lipid indirectly, from engulfed apoptotic cells with large nLDs.
(TIF)
