# Supplementary figures and images for: Nuclear lipid droplets and nuclear damage in Caenorhabditis elegans
Source: PLoS Genet. 2021 Jun 16;17(6):e1009602. doi: 10.1371/journal.pgen.1009602 (PMC8208577; doi:10.1371/journal.pgen.1009602)

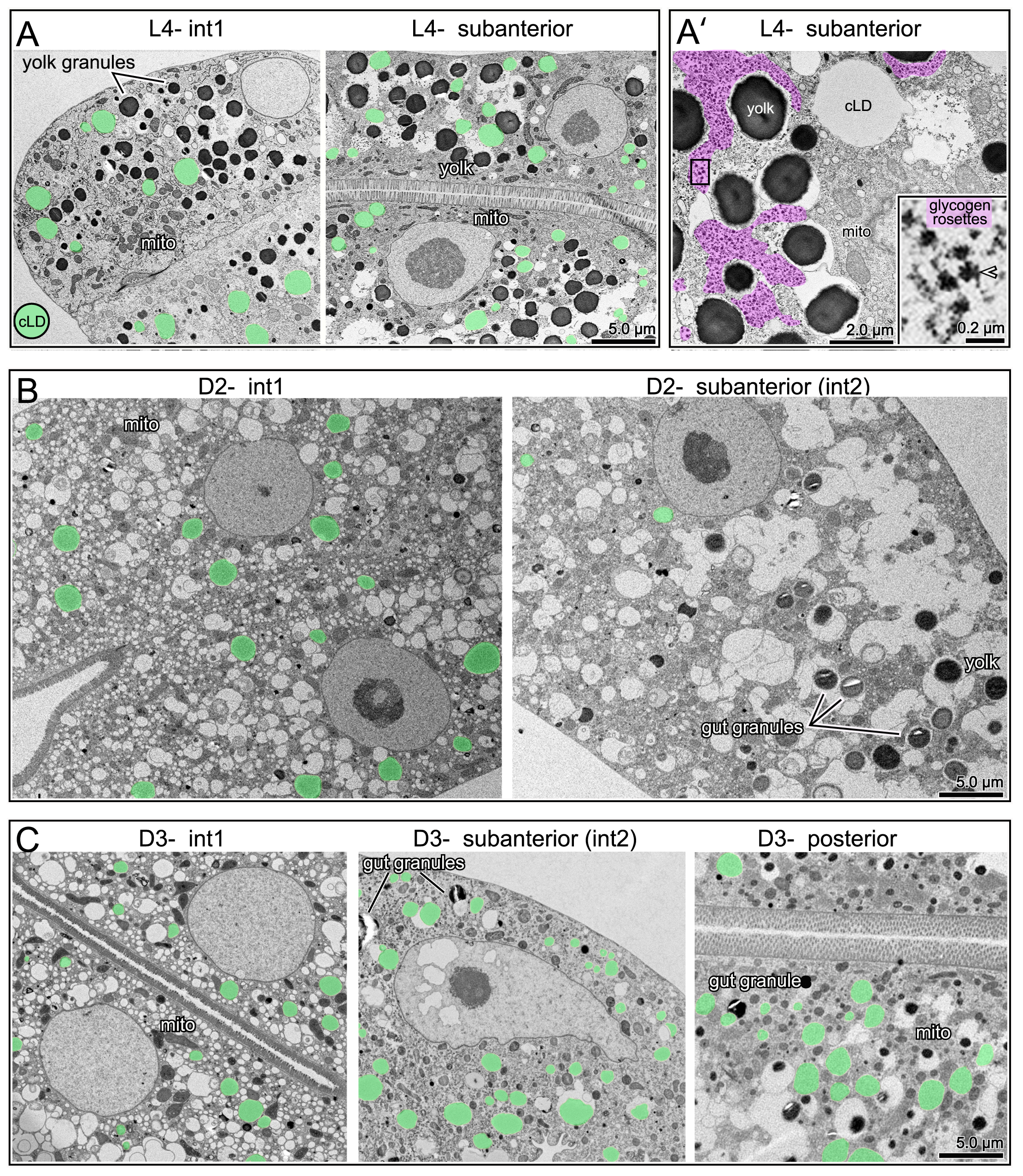

Supplement: S1 Fig — (A-C) TEM of intestinal cell cytoplasm in L4, D2, and D3 hermaphrodites; cLDs are tinted green. L4 cells have large numbers of cLDs, yolk granules (large, electron-dense bodies) and islands of glycogen (magenta); the inset shows the distinctive shapes of glycogen rosettes (arrowhead). By the D2 stage, all cells have lost large amounts of yolk granules and glycogen, but subanterior cells show the greatest loss of cLDs. Note the increase in gut granules, which can be distinguished from yolk granules by their distinctive staining and morphology. mito = mitochondria. (TIF) [file pgen.1009602.s001.tif]

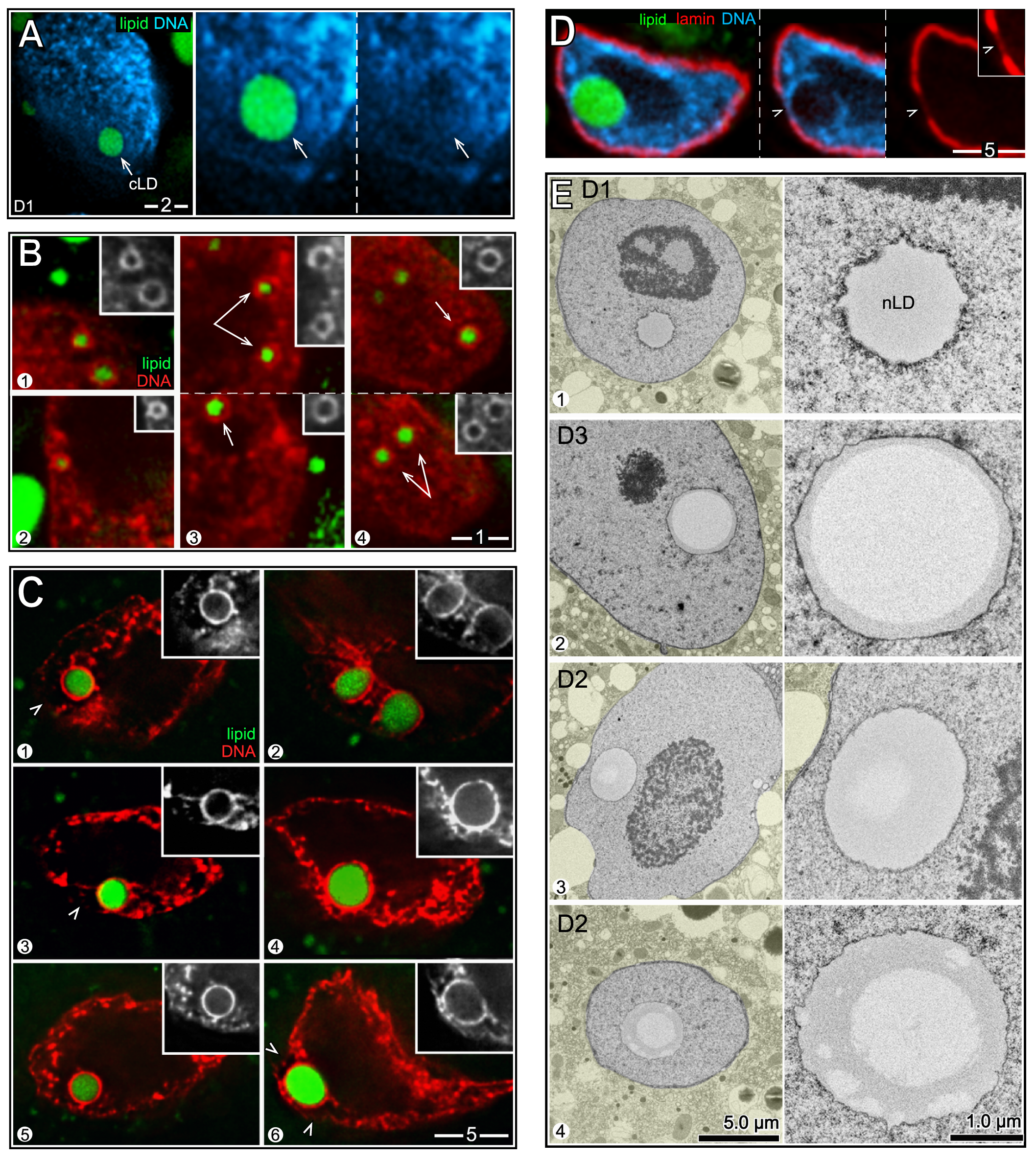

Supplement: S2 Fig — (A) The image shows the surface of an intestinal nucleus stained for lipid (green, BODIPY) and DAPI. The lipid droplet is a cLD that is partially embedded in the nuclear envelope. The inset shows that even large cLDs such as this do not create artificial, ring-like patterns in the peripheral heterochromatin. Thus, the presence of a heterochromatin ring or coat can be used to distinguish an nLD from an envelope-embedded cLD, even without immunostaining for the envelope. See S7 Fig for an example of a germ nucleus with an envelope-embedded cLD. (B) Examples of small nLDs with heterochromatin coats in intestinal cells that were stained for lipid (green, BODIPY) and DNA (DAPI staining shown in red for contrast), but that were not detergent permeabilized; the insets show the heterochromatin rings (white) separately. Note that multiple nLDs are visible on different focal planes of single nuclei. A comparable frequency of nLDs was observed in our TEM analysis (compare panel 3 in Fig 2F), but is higher than observed in immunostaining experiments with detergent permeabilization. Moreover, some nLDs as in panel 2 were smaller than any nLDs detected in detergent-permeabilized cells. (C) Examples of large nLDs with heterochromatin coats. Note neighboring regions of the envelope that appear deficient in heterochromatin (arrowheads). (D) D2 nucleus with an apparent lamin-deficient region at the base (arrowhead) of a large nLD. The overexposed lamin channel (inset) shows that the lamina appears to be continuous. (E) TEM images of D1 and D2 nuclei showing variation in the shapes and staining patterns of some nLDs; similar variation also was observed for cLDs. Note that each of the nLDs has an electron-dense coat. (TIF) [file pgen.1009602.s002.tif]

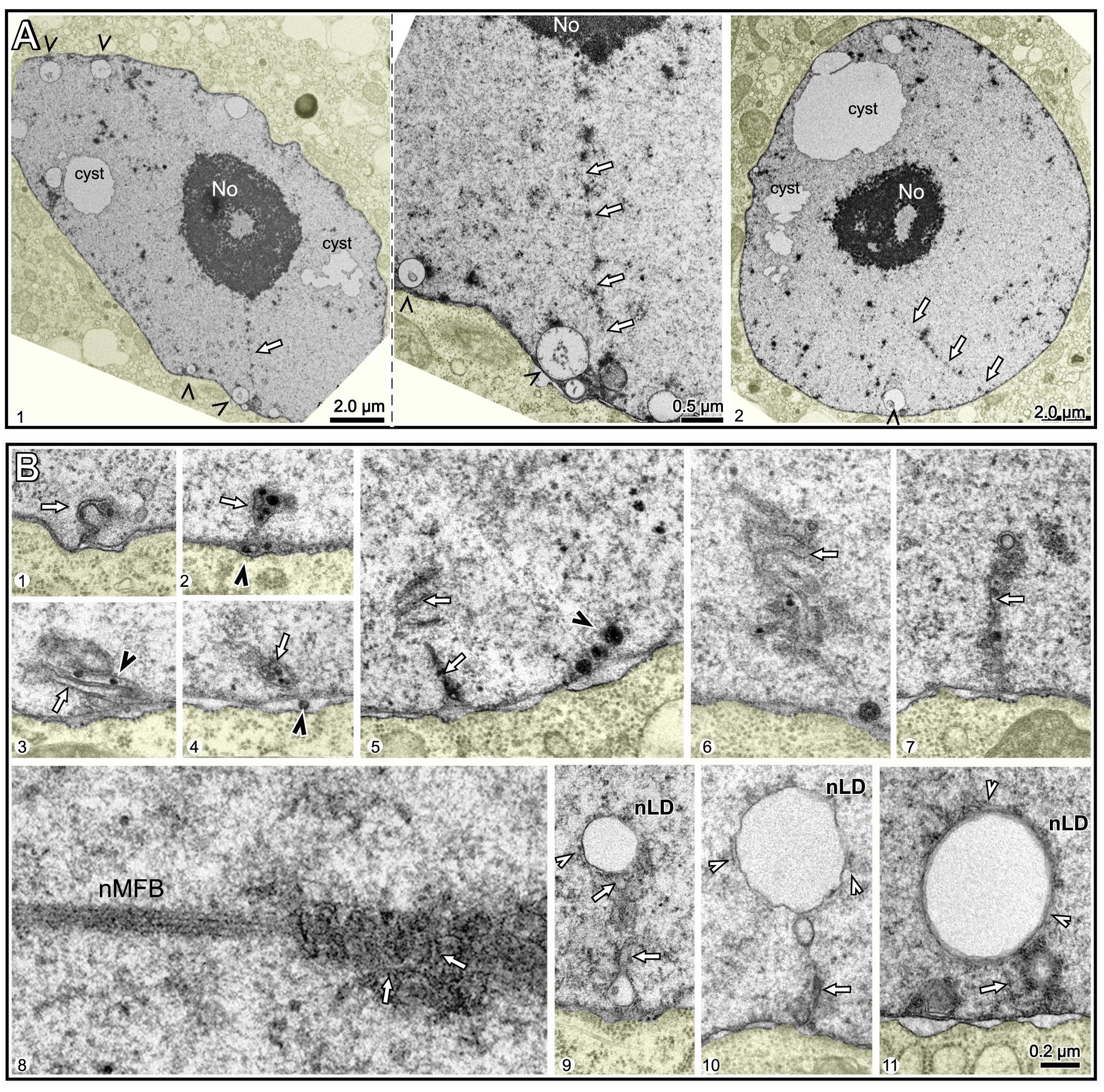

Supplement: S3 Fig — (A) Examples of type I nuclear tubules (arrows) that appear to extend between the envelope and the nucleolus. Note the cysts and kernel vesicles (black arrowheads) in the nuclei. (B) Images showing the varied shapes of nuclear tubules, including zig-zag patterns (panels 5–8). Panels 2 and 4 show electron-dense granules in the perinuclear cistern (black arrowheads). Panels 9–11 show examples of tubules (arrows) associated with nLDs; note the apparent membrane fragments at the perimeter of the nLDs (white arrowheads). (TIF) [file pgen.1009602.s003.tif]

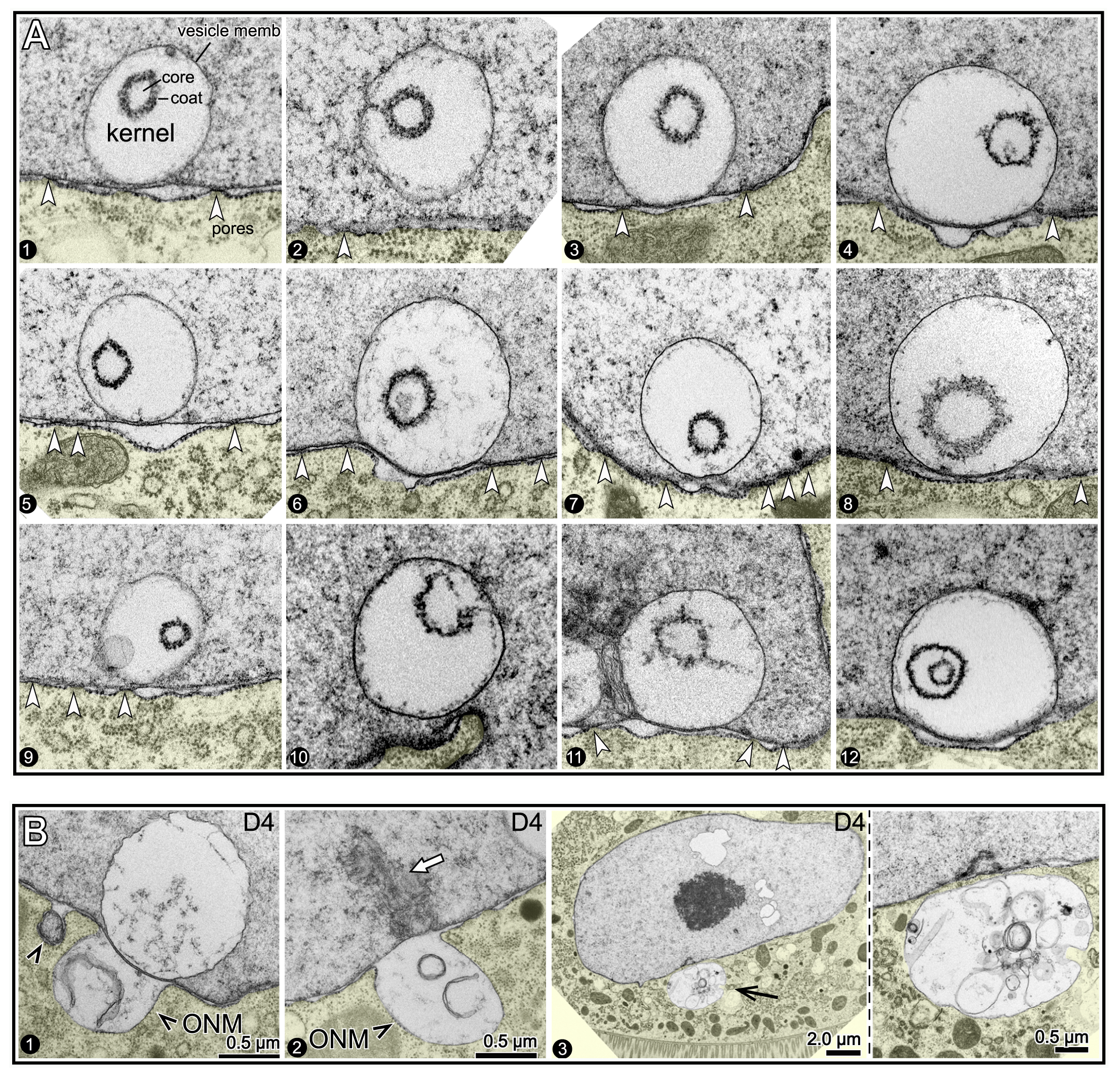

Supplement: S4 Fig — (A) Variation in the appearance of kernel vesicles in D1 and D2 nuclei. Note the absence of nuclear pores (white arrowheads) at the base of each kernel vesicle. (B) Examples of membrane-enclosed, degraded material at or near the envelope of D4 nuclei. Panels 1 and 2 show protrusions (arrowheads) of the ONM that appear to contain membrane fragments and other debris. Note that the protrusion in panel 2 is adjacent to multiple nuclear tubules (arrow). Panel 3 shows a D4 nucleus adjacent to a large, membrane-enclosed vesicle (arrow), that appears to be filled with debris (inset). (TIF) [file pgen.1009602.s004.tif]

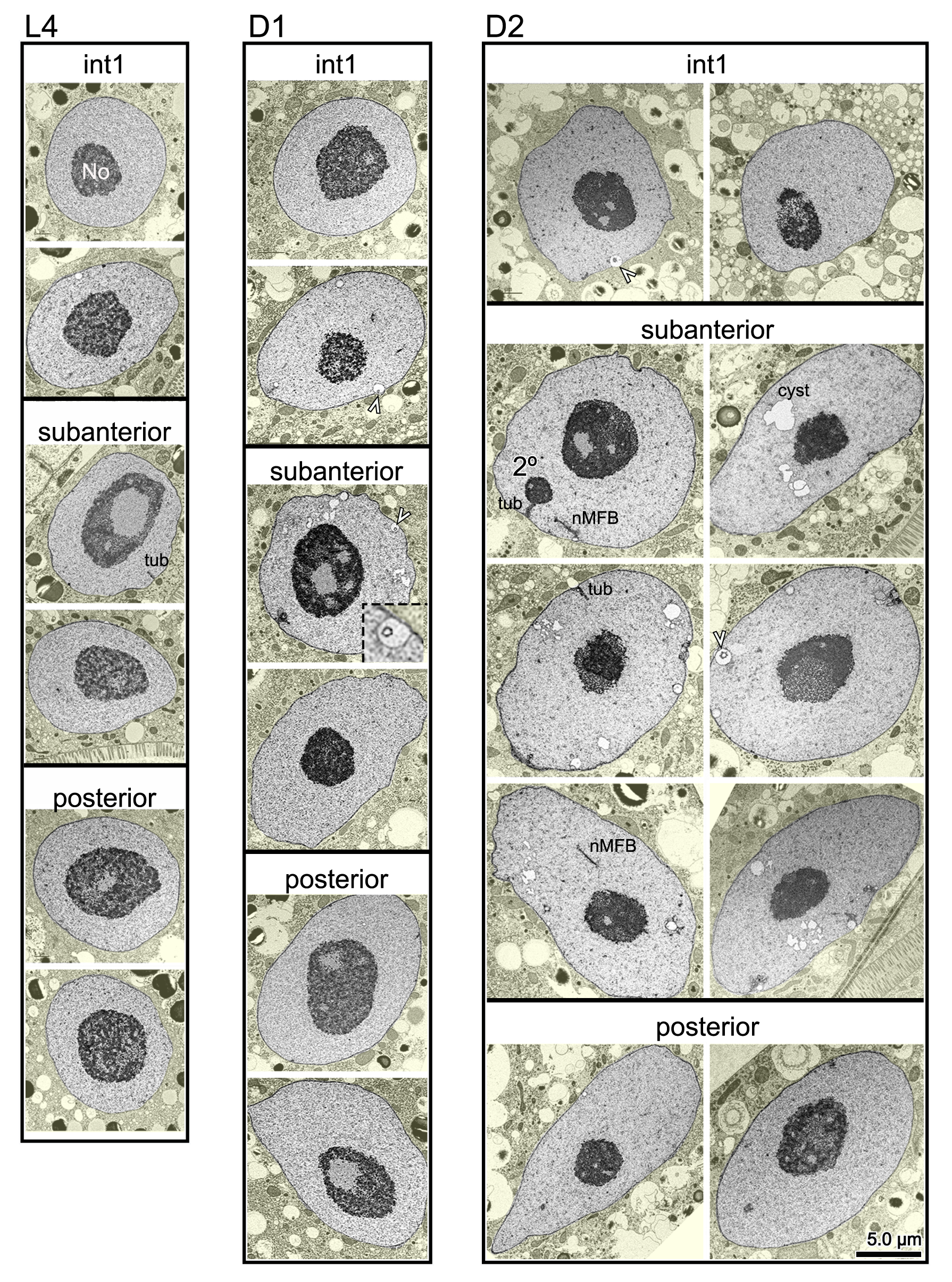

Supplement: S5 Fig — Representative images of L4, D1, and D2 nuclei from the int1, subanterior, and posterior regions of the intestine as indicated. The L4 and D1 nuclei generally have relatively clear nucleoplasm, although a few D1 nuclei have kernel vesicles (arrowheads and inset). More kernel vesicles are apparent in D2 nuclei, along with tubules (tub), nuclear microfilament bundles (nMFBs) and a few cysts. A secondary nucleolus is visible in one of the D2 subanterior nuclei. (TIF) [file pgen.1009602.s005.tif]

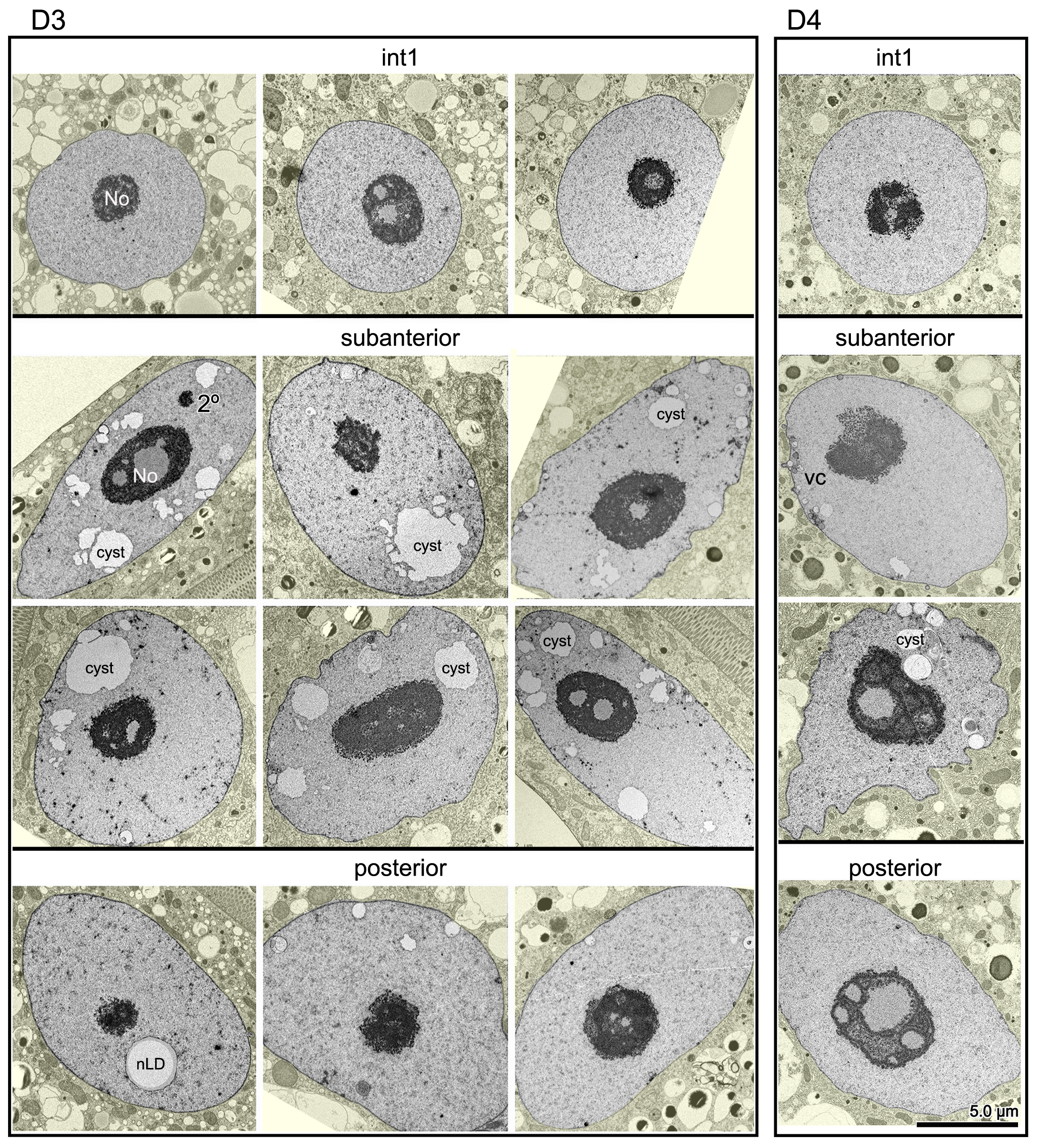

Supplement: S6 Fig — Representative images of D3 and D4 nuclei from the regions of the intestine indicated, labeling as for S5 Fig. Note that several D3 subanterior nuclei have clumps of electron-dense material in the nucleoplasm that are not generally present in int1 nuclei or most posterior nuclei, and are not present in L4-D2 nuclei (S5 Fig). (TIF) [file pgen.1009602.s006.tif]

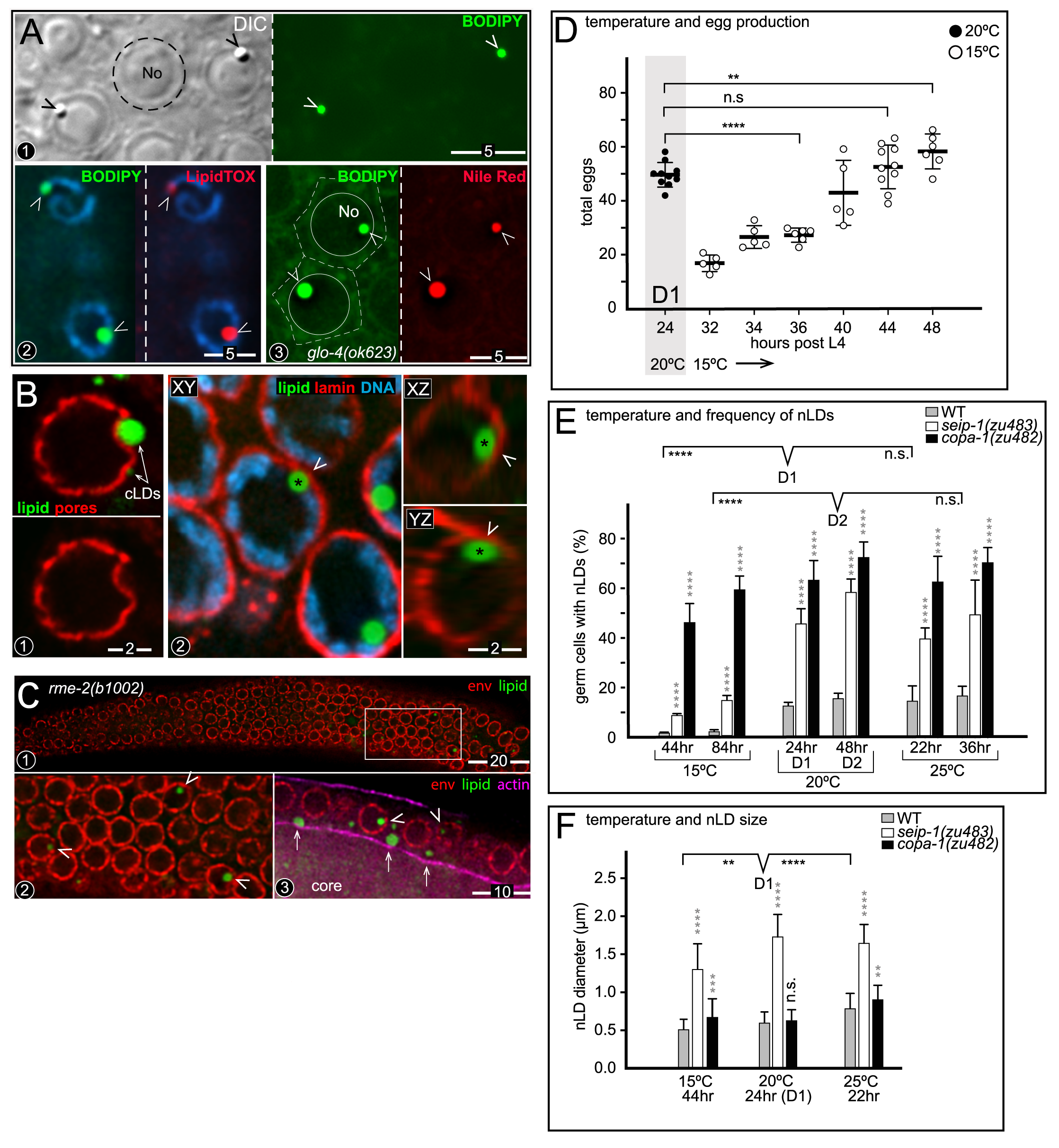

Supplement: S7 Fig — (A) These experiments address the specificity of the lipid stains for germ cell nLDs. Studies on somatic fat in live C. elegans found some commonly used lipid dyes stained lysosome-related organelles in addition to lipid droplets [124]. Panel 1 shows that nLDs visible by DIC in fixed tissues also stain with BODIPY, panel 2 shows that BODIPY-stained nLDs also stain with LipidTOX, and panel 3 shows that glo-4 (ok623) mutants contain BODIPY and Nile Red-stained nLDs; the glo-4 mutants lack cytoplasmic lysosome-related organelles that can stain with lipid dyes. n = 18–25 gonads for each experiment. (B) Panel 1 shows an example of an envelope-embedded cLD in a germ nucleus. These are rare, likely from the lack of cLDs in most germ cells, but need to be distinguished from nLDs. Panel 2 shows an example of orthogonal planes used to verify an nLD (asterisk) is entirely within the envelope. (C) This experiment addresses whether yolk is a determinant of nLD formation. The image shows a rme-1(b1002) mutant gonad stained for the envelope (red, NPP-9/RanBP2), lipid (green, BODIPY), and F-actin (magenta, phalloidin). These mutants are unable to take up yolk lipoproteins, but appear to have normal numbers of nLDs (arrowheads) in the peak zone (panel 2; n = 24 gonads). The rme-2 mutants often have what appear to be giant cLDs around germ nuclei (arrows in panel 3). However, inspection of the germ cell membranes showed that this lipid is between, but outside of, germ cells. We presume this material consists of yolk lipoproteins that accumulate in the body cavity in rme-2 mutants. (D) Calibration of temperature-equivalent worm ages based on egg production. The plot shows that 20°C animals analyzed as D1 adults (24 hours post-L4) have produced an average of 50 eggs at the point of analysis. Thus, we compared 20°C D1 adults with 15°C adults that have produced similar numbers of eggs, which the graph shows occurs at 44 hours post-L4. Similarly, we compared 20°C D1 adults with 25°C adult [file pgen.1009602.s007.tif]

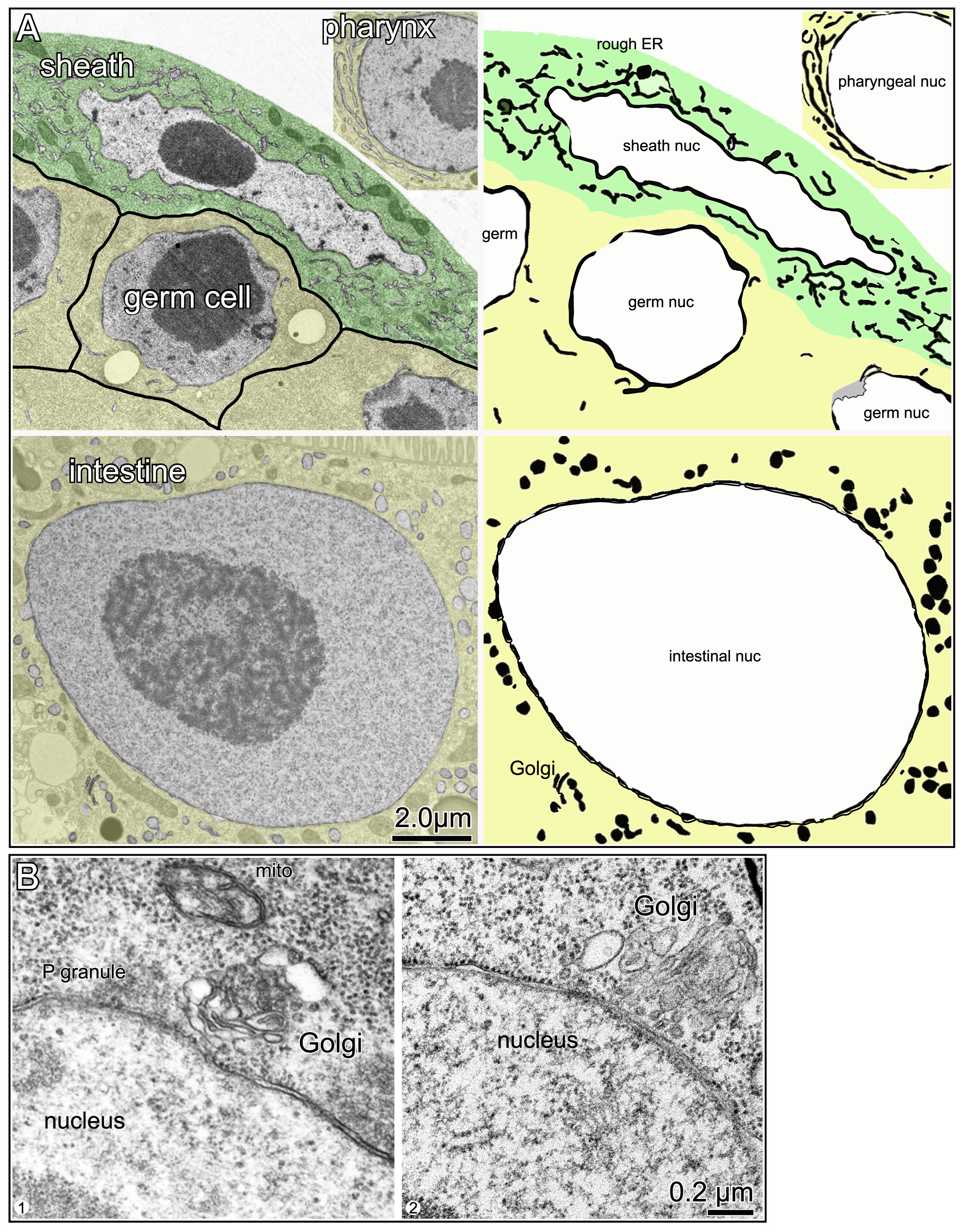

Supplement: S8 Fig — (A) The left column shows TEM images of a pharyngeal cell, a gonad sheath cell, a germ cell, and an intestinal cell, all at the same magnification. The black outlines in the diagrams at right indicate the nuclear membranes and ER membranes. Note that germ cells have little ER compared to any of the somatic cell types, and that the nuclear envelope is by far, the major ER subdomain in germ cells. (B) Examples of rare, presumptive Golgi stacks in germ cells; only two such examples were found in over 2000 germ cells examined by TEM. Pg = P granule, mito = mitochondria. (TIF) [file pgen.1009602.s008.tif]

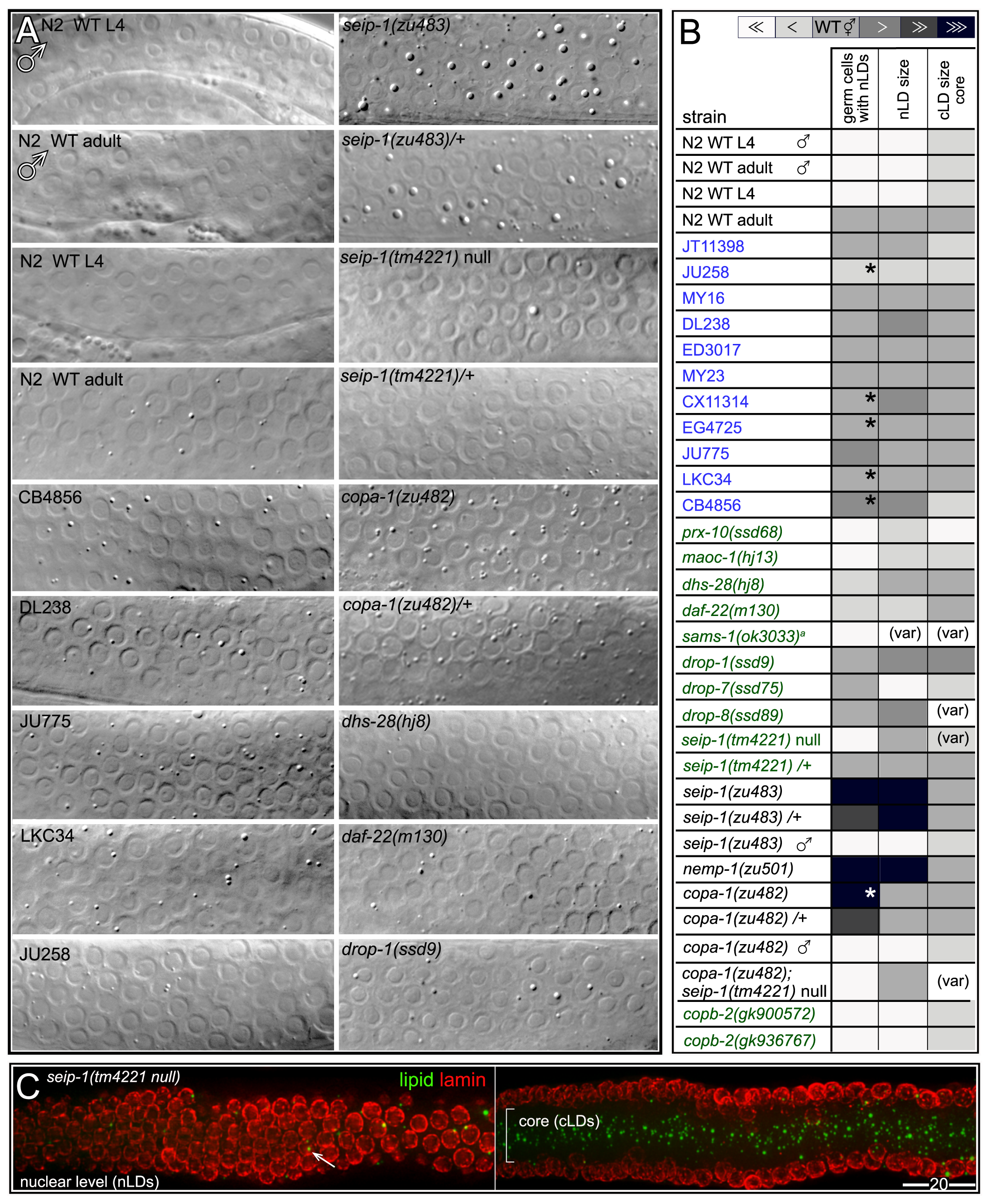

Supplement: S9 Fig — (A) DIC images of gonads in C. elegans wild strains or mutants as indicated; the region shown corresponds to the peak zone of nLDs in wild-type gonads. (B) Qualitative, 6-bin scale (top) comparing the nLDs and cLDs in N2 wild-type hermaphrodites with those in the strains and mutants listed. N2 is the laboratory strain of C. elegans; other wild strains are indicated in blue. Mutants isolated in previous studies but not analyzed for nLDs are indicated in green and referenced in the text. 20–30 live, anaesthetized animals were scored for each strain. Asterisks indicate wild strains or mutants where individual germ nuclei appeared to contain multiple nLDs more often than observed in N2 wild-type hermaphrodites. var = variable. (C) Gonad from a seip-1(tm4221) null mutant stained for lipid (green, BODIPY) and lamin (red, LMN-1). The image at left is a 5 μm maximum intensity z-projection through the top nuclear level; the few lipid droplets visible are all cLDs; compare with similar optical plane through a seip-1(zu483) gonad in Fig 15H. The panel at right is a 5 μm maximum intensity z-projection through the gonad core, showing relatively abundant cLDs. Scale bar in microns, as labeled. (TIF) [file pgen.1009602.s009.tif]

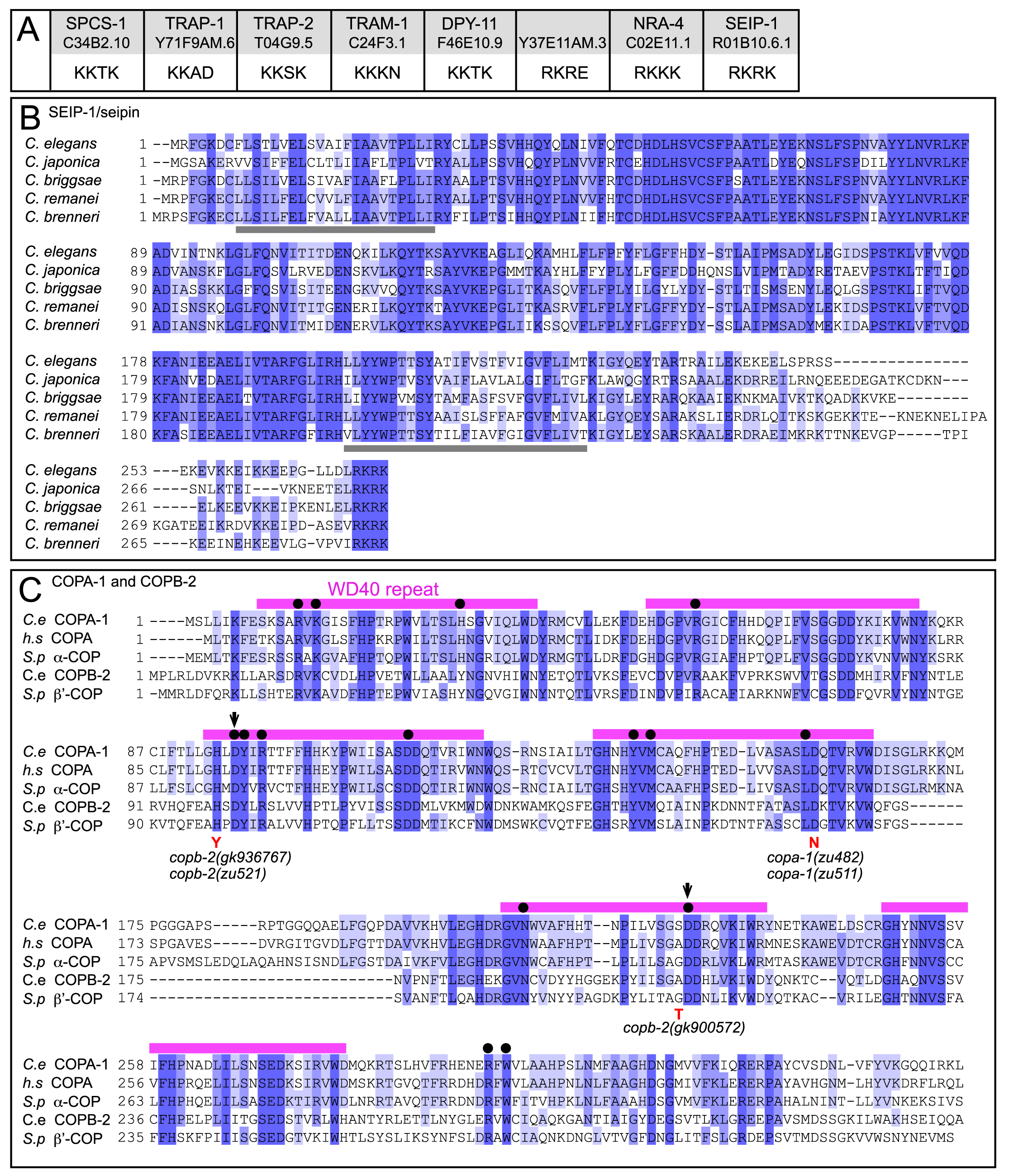

Supplement: S10 Fig — (A) The dibasic retrieval motif has not been studied in C. elegans, but several proteins appear to contain the motif. Shown here are predicted ER-resident membrane proteins in C. elegans (see text) with C-terminal peptides that (1) are conserved in other Caenorhabditis species, and (2) conform with the dibasic peptide retrieval motifs KKxx and RKxx. The proteins SPCS-1, TRAP-1, TRAP-2, TRAM-1, and NRA-4 have been confirmed as ER proteins [125,126]. Y37E11AM.3 is an ortholog of human FVT-1/KDSR (3-ketodihydrosphingosine reductase), which has been localized to the ER [127]. DPY-11 has been localized in C. elegans to subcellular membranous organelles consistent with the ER [128], and the human ortholog, TMX, has been localized to the ER [129]. (B) Alignment of the ER-resident membrane protein SEIP-1/seipin in C. elegans and other Caenorhabditis species. The gray bars indicate predicted membrane-spanning domains. Note that the cytoplasmic domain, after the second membrane-spanning domain, shows little conservation other than the C-terminus, an RKxx-type retrieval motif. (C) N-terminal half of COPA-1/α-COP and COPB-2/β’-COP in C. elegans aligned with the homologous human and S. pombe sequences; WD40 repeats are indicated in magenta. Residues that form the binding pocket for dibasic retrieval peptides in ER-resident membrane proteins are indicated by black circles, and two critical acidic residues are indicated by arrows [data from [77]]. The copa-1 and copb-2 mutations described in this paper are indicated, with amino acid substitutions shown in red. The mutation copa-1(zu511) was isolated in a different screen than copa-1(zu582), but causes the same amino acid substitution. Sequence data compiled from Wormbase WS270 (https://wormbase.org). (TIF) [file pgen.1009602.s010.tif]

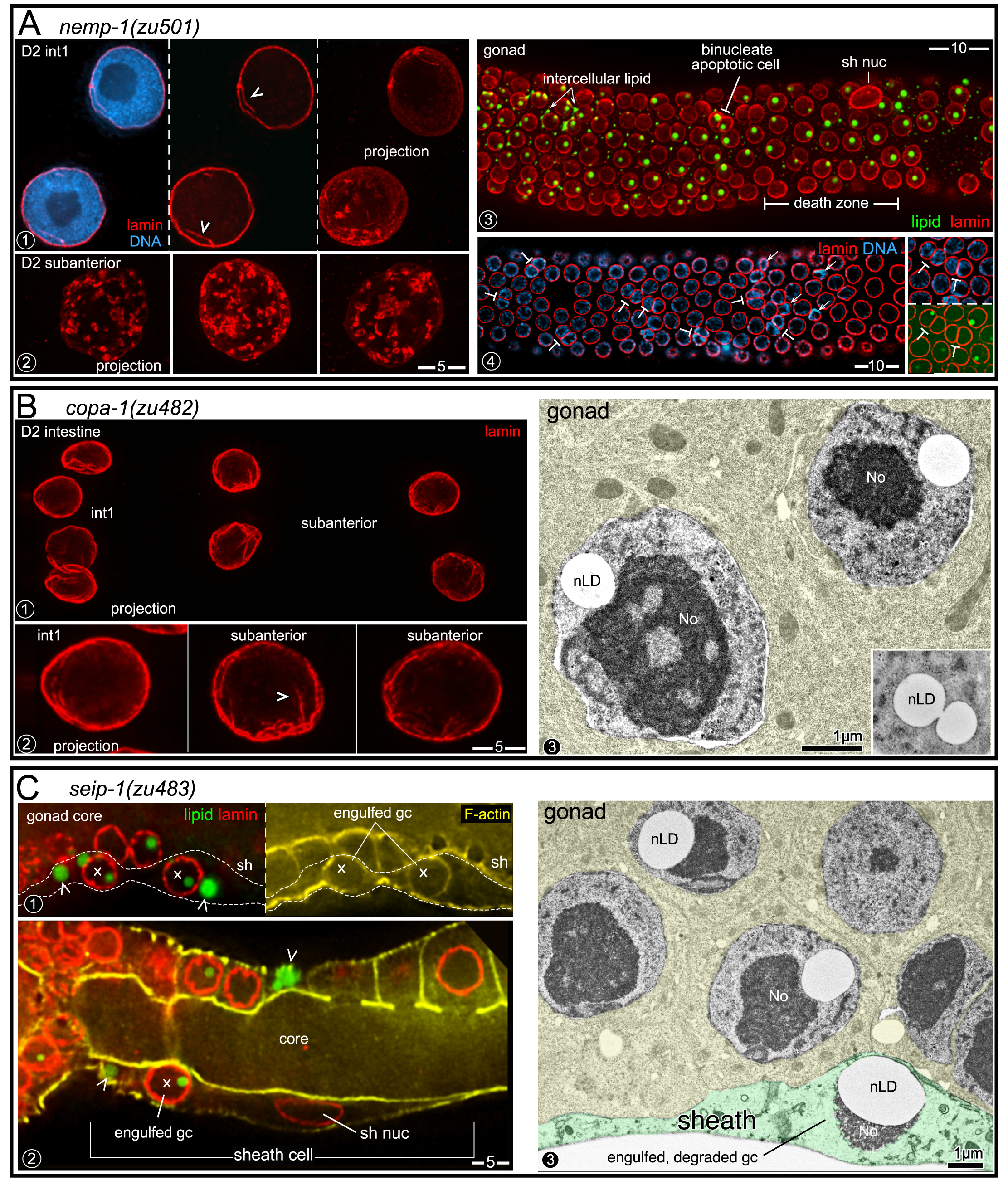

Supplement: S11 Fig — (A) nemp-1(zu501). nemp-1 null mutants were described recently by others and shown to cause variable defects in fertility, brood size, and egg viability [72]. The semi-dominant nemp-1(zu501) mutant produces some dead eggs, but is homozygous viable and appears superficially healthy with normal body morphology and growth rates. nemp-1(zu501) intestines generally resembled wild type; for example, cLDs appeared normal and some nuclei contained nLDs (Table 1). At the D2 stage, fat was depleted predominantly from the subanterior region, and int1 nuclei at the D2 stage (panel 1; arrowheads indicate lamin lines) appeared rounder and "healthier" than subanterior nuclei (panel 2; n = 28 intestines). However, several subanterior nuclei (panel 2) contained far greater numbers of lamin sacs than observed in wild type, suggesting either a defect in sac removal or in events that trigger sac formation. nemp-1 gonads superficially resembled wild type, except for the enlarged nLDs (panel 3; n = 28 gonads). Germ nuclei appeared to increase in size uniformly as they progressed through pachytene, the gonads had small numbers of binucleate (T-bar) and mononucleate apoptotic cells, and no nLDs were present in sheath cell nuclei (sh nuc). D2 gonads could accumulate variable, and sometimes large, amounts of intercellular lipid (presumably yolk lipoproteins) between germ cells, as observed with rme-2(b1002) mutants (see S7 Fig). However, 7/28 nemp-1(zu501) gonads had excessive numbers of binucleate cells (panel 4); nLDs could be present in one, both, or neither of the germ nuclei (inset). In addition, several nemp-1(zu501) gonads had a few germ nuclei with either larger, or fewer, numbers of chromosomes than wild type, suggesting a defect in mitosis. Because nLDs are not found in mitotic cells, and most nLDs form after binucleate cells develop [38], we consider it unlikely that nLDs cause the mitotic and binucleate cell phenotypes in nemp-1(zu501) mutants. (B) copa-1(zu482). copa-1 null muta [file pgen.1009602.s011.tif]
